# c-Src-induced vascular malformations require localised matrix degradation at focal adhesions

**DOI:** 10.1242/jcs.262101

**Published:** 2024-07-10

**Authors:** Patricia Essebier, Mikaela Keyser, Teodor Yordanov, Brittany Hill, Alexander Yu, Ivar Noordstra, Alpha S. Yap, Samantha J. Stehbens, Anne K. Lagendijk, Lilian Schimmel, Emma J. Gordon

**Affiliations:** ^1^ Centre for Cell Biology of Chronic Disease, Institute for Molecular Bioscience, The University of Queensland, St. Lucia, Brisbane, Queensland, Australia 4072; ^2^ Australian Institute for Bioengineering and Nanotechnology, The University of Queensland, St. Lucia, Brisbane, Queensland, Australia 4072

**Keywords:** Angiogenesis, c-Src, Extracellular matrix, Focal adhesions

## Abstract

Endothelial cells lining the blood vessel wall communicate intricately with the surrounding extracellular matrix, translating mechanical cues into biochemical signals. Moreover, vessels require the capability to enzymatically degrade the matrix surrounding them, to facilitate vascular expansion. c-Src plays a key role in blood vessel growth, with its loss in the endothelium reducing vessel sprouting and focal adhesion signalling. Here, we show that constitutive activation of c-Src in endothelial cells results in rapid vascular expansion, operating independently of growth factor stimulation or fluid shear stress forces. This is driven by an increase in focal adhesion signalling and size, with enhancement of localised secretion of matrix metalloproteinases responsible for extracellular matrix remodelling. Inhibition of matrix metalloproteinase activity results in a robust rescue of the vascular expansion elicited by heightened c-Src activity. This supports the premise that moderating focal adhesion-related events and matrix degradation can counteract abnormal vascular expansion, with implications for pathologies driven by unusual vascular morphologies.

## Teaser

**Highlighted Article:** Constitutively active c-Src (Y527F) in 3D vascular models induces a ballooning phenotype due to local secretion of MMPs at focal adhesions in endothelial cells.

## INTRODUCTION

The blood vascular network is largely formed by sprouting angiogenesis and is required to supply oxygen and nutrients to all tissues in the body (Potente et al., 2011). Controlled sprouting and patterning of the vasculature is essential during embryonic development, with aberrant angiogenesis also occurring later in life in response to tissue injury or in disease. The binding of vascular endothelial growth factors (VEGFs) to vascular endothelial growth factor receptors (VEGFRs) is considered to be largely responsible for initiating sprouting (Simons et al., 2016), driving downstream signalling to induce endothelial cell (EC) identity, migration and proliferation. Sprouting angiogenesis and vascular integrity is mediated by endothelial cell–cell contacts, which in turn are controlled by the junctional localisation of vascular endothelial-cadherin (VE-cadherin; also known as CDH5) (Giannotta et al., 2013; Szymborska and Gerhardt, 2017). Angiogenesis and cell–cell adhesion are also intricately associated with cell–matrix adhesions, with increasing evidence that if adhesions are disrupted, ECs display altered VE-cadherin localisation, sprouting and permeability (Chau et al., 2022; Pulous et al., 2019; Wang et al., 2019; Wu et al., 2015b; Yamamoto et al., 2015).

Cell anchoring to the extracellular matrix (ECM) is primarily mediated through focal adhesions (FAs), which are large dynamic multi-protein complexes (Mavrakis and Juanes, 2023). Matrix-bound transmembrane integrin receptors interact with intracellular signalling molecules, including focal adhesion kinase (FAK; also known as PTK2), c-Src and paxillin. Within FAs, the mechano-effector proteins vinculin and talin 1 directly interact with the actin cytoskeleton, undergoing conformational changes in response to tension via a process known as mechanotransduction (Goult et al., 2021; 2018). There is increasing evidence that FAs and cell–cell adhesions share significant crosstalk via actin scaffolding processes (Mui et al., 2016; Weber et al., 2011).

It is well appreciated that the non-receptor Src family kinases play crucial roles in both cell–cell adhesion and cell–matrix adhesion, with their localisation and activity regulated in a context-dependent manner. VE-cadherin phosphorylation by Src family kinases (SFKs) at distinct tyrosine sites within its intracellular tail mediates its localisation in endothelial cells, and controls vascular integrity and angiogenic sprouting (Gordon et al., 2016; Jin et al., 2022; Ninchoji et al., 2021; Orsenigo et al., 2012; Schimmel and Gordon, 2018; Smith et al., 2020). Although c-Src was traditionally thought to be primarily responsible for phosphorylation of VE-cadherin, recent advances in Cre-driven endothelial-specific mouse models identified that the SFK Yes (also known as YES1), rather than c-Src, mediates VE-cadherin phosphorylation, turnover and junctional plasticity (Jin et al., 2022). In agreement, endothelial-specific deletion of c-Src does not lead to gross defects in VE-cadherin internalisation. Rather, loss of c-Src leads to reduced retinal angiogenesis due to loss of FA assembly and cell–matrix adhesion, resulting in loss of sprout stability (Schimmel et al., 2020). This suggests that SFKs play different roles in FA crosstalk to cell–cell adhesions, leading to a context-dependent function in vascular growth and physiology.

In addition to facilitating adhesion to the surrounding environment, FAs have been shown to play a role in remodelling of the ECM, acting as sites of localised exocytosis. It has been previously demonstrated that microtubules target and anchor at FAs, facilitating delivery of exocytic vesicles (Nolte et al., 2020). In the context of migrating keratinocytes, this results in localised secretion of the matrix metalloproteinase (MMP) MT1-MMP (also known as MMP14), releasing adhesions from the surrounding matrix, and resulting in FA turnover. This directly associates FAs with the intracellular machinery regulating exocytosis, a fundamental process where molecules are transported to the cell surface, where they can be either released into the extracellular space or integrated into the plasma membrane (Wu et al., 2015a). Microtubules act as the major tracks for transport within cells, where vesicles are transported along these dynamic, tube-like structures by kinesin or dynein motors to the plasma membrane for exocytosis (Noordstra and Akhmanova, 2017). Exocytosis is crucial for a range of cellular processes, such as synaptic neurotransmission, paracrine signalling and the release of hydrolytic enzymes, allowing the turnover of FAs, which is crucial for coordinated cell movement (Stehbens et al., 2014). Although MMPs are known to be crucially important for a wide range of vascular processes, including angiogenesis, morphogenesis and wound repair (Bordeleau et al., 2017; Wang and Khalil, 2018), precisely how alterations in MMP activity and localisation are regulated, and whether this is mediated at endothelial FAs, remains unclear.

Here, we sought to investigate the consequence of elevated c-Src activity on EC adhesions and vascular behaviour. We found that introducing a constitutively active (Y527F) c-Src mutation (c-Src-CA) into ECs in 3D vascular models resulted in a vast ballooning phenotype. These vessels lacked functional angiogenic sprouts and a continuous cellular lining of the vessels. The c-Src-CA mutation induced large FAs, elevated paxillin and VE-cadherin phosphorylation, and loss of cell–cell junctions. We found that local secretion of MMPs at these enlarged FAs resulted in degradation of ECM components, with broad-spectrum pharmacological inhibition of MMPs rescuing the vascular ballooning induced by constitutively active c-Src. Thus, our work clearly shows the importance of tightly controlled FA formation and turnover, mediated by c-Src, in enabling functional angiogenesis.

## RESULTS

### Elevated c-Src activity induces vascular malformations

Loss of endothelial c-Src results in decreased vascular stability due to loss of cell–matrix adhesions (Schimmel et al., 2020). We further sought to determine how elevated c-Src activity would affect EC behaviour. Addition of a glycine/serine-rich flexible linker between the C-terminus and a fluorescent tag ensures normal regulation of c-Src protein activation (Sandilands et al., 2004) (Fig. S1A). We generated several c-Src–mScarlet (mSc) fusion proteins including wild type (c-Src-WT–mSc), constitutively active (c-Src-CA–mSc), which has a Y527F mutation preventing phosphorylation of the inhibitory Tyr527 (Roskoski, 2005), and dominant negative (c-Src-DN–mSc), which has Y527F/K295R mutations holding the protein in its open confirmation to bind target proteins but a dysfunctional kinase domain (Shvartsman et al., 2007) (Fig. S1A–C). Expression of the c-Src–mScarlet fusion proteins in human umbilical vein ECs (HUVECs) using a lentiviral-based system led to a significant increase in c-Src protein levels, which was not altered by either the Y527 or Y527F/K295R mutations (Fig. S1D,E).

**Fig. 1.**
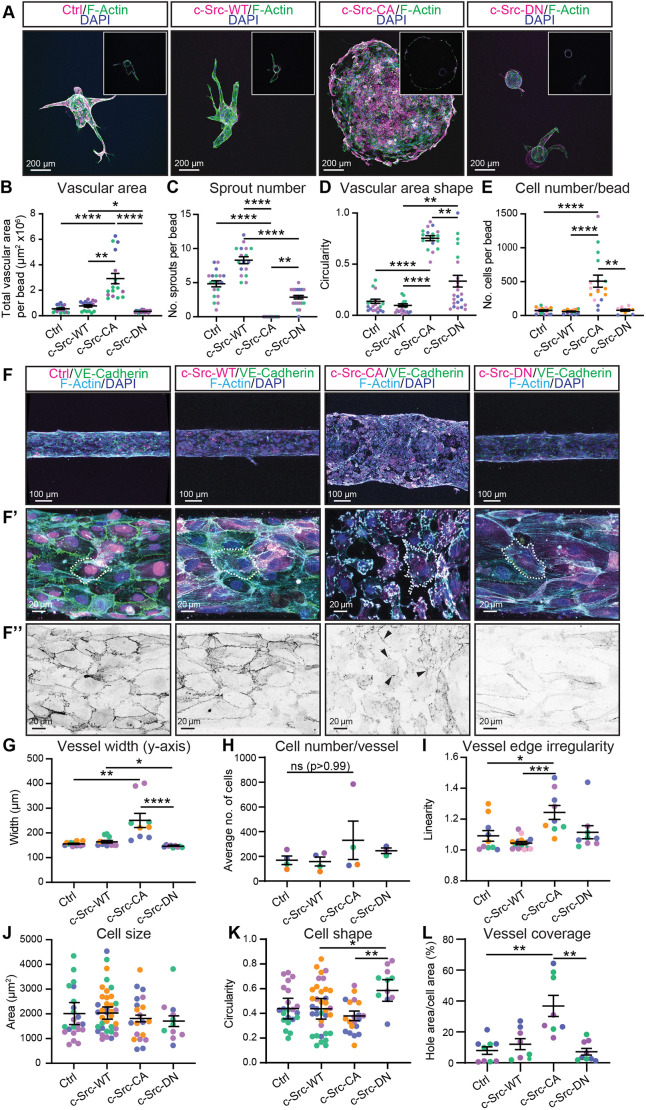
**Constitutively active c-Src induces ballooning of *in vitro* 3D vasculature.** (A) Representative images of fibrin bead sprouts. HUVECs transduced with mScarlet-tagged c-Src WT, mutants (CA or DN) or control (Ctrl; empty vector mScarlet) (magenta) were grown in a 5 mg/ml fibrin gel bead sprouting assay for 7 days before fixation. Immunofluorescence staining was performed for F-actin (phalloidin; green) and nuclei (DAPI; blue). Inserts show single *Z*-planes for the cross section of the vascular area. (B–E) Quantification of sprout parameters: vascular area (B), number of sprouts per bead (C), shape of vascular area (D), and number of cells per bead (E); *n*=3–5 independent experiments. (F) Representative images of HUVECs transduced as in A (magenta) seeded in PDMS microfluidic vessels containing 2.5 mg/ml collagen matrix for 3 days before fixing. Immunofluorescence staining was performed for VE-cadherin (green), F-actin (phalloidin; cyan) and nuclei (DAPI; blue). Low magnification (F) was used to quantify vessel width (G), number of cells per vessel (H), and vessel edge irregularity (I); *n*=3–5 independent experiments, three vessels per replicate with each data point representing an average of 3–5 measurements per vessel. High magnification (F′) was used to quantify individual cell morphology within microvessels as cell size (J), cell shape (K), and vessel coverage as a percentage of total vessel area (L). *n*=3–5 independent experiments, 3–5 images per replicate with each data point representing an average of 1–5 cells per image. The dotted outline in F′ highlights an individual cell. High magnification of VE-cadherin channel (F″) with black arrowheads indicating remaining cell–cell junctions. All data are represented as mean±s.e.m. with individual data point indicated; colours represent independent experiments. *P<0.05, **P<0.01, ***P<0.001, ****P<0.0001 (Kruskal–Wallis test with Dunn's multiple comparisons). The mean value of each individual replicate and corresponding s.e.m., instead of all data points separately, was used for statistical analysis in I and J.

To assess how elevated c-Src activity altered vascular sprouting in 3D, we embedded microcarrier beads coated with control and c-Src mutant-expressing ECs within fibrin gels (Nakatsu et al., 2007). After 7 days, c-Src-WT cells displayed a modest increase in the number of sprouts, although there was no significant increase in vascular area or total cell number (Fig. 1A–E). Introduction of the c-Src-CA mutation induced a drastic alteration in morphology, where instead of distinctive sprouts, a rapid radial migration in a lace-like discontinuous pattern (hereafter referred to as ballooning) was observed, with an accompanying increase in the number of cells per bead (Fig. 1A–E; Movie 1). The c-Src-DN mutation showed no phenotype distinguishable from control (Fig. 1A–E), suggesting that the ballooning morphology was a result of increased c-Src kinase activity. Additionally, the c-Src-DN cells displayed decreased vascular area and fewer sprouts than control or c-Src-WT cells (Fig. 1B–E), confirming this mutation suppresses endogenous c-Src signalling in a dominant negative fashion.

The evolutionarily conserved Notch pathway is involved in tissue patterning processes, and is essential for tip and stalk cell specification (Blanco and Gerhardt, 2013; Gerhardt et al., 2003). We hypothesised that the ballooning phenotype of c-Src-CA cells might be due to alterations in Notch signalling and resulting tip and stalk cell selection. However, no change in tip cell number was observed in mice with a loss of endothelial c-Src, suggesting that c-Src does not impact Notch activity (Schimmel et al., 2020). In agreement, no significant changes in DLL4 expression were observed upon c-Src manipulation (Fig. S1F,G), confirming the ballooning phenotype is not a result of altered Notch activity or tip and stalk cell specification.

To further investigate the role of c-Src activity in 3D, we assessed the behaviour of c-Src–mSc cells in microfabricated vessels (Polacheck et al., 2019; 2017). Here, ECs were surrounded by a Type I collagen matrix and subjected to oscillatory flow (∼ >3 dynes/cm^2^) by rocking. Consistent with the observations in the bead sprouting assay, vessels consisting of c-Src-CA cells displayed a ballooning phenotype after 3 days of culture (Fig. 1F; Movie 2), resulting in a significant increase in vessel width compared to vessels comprising control cells (Fig. 1G). Neither c-Src-WT nor c-Src-DN microvessels displayed significant changes in vessel width, revealing that c-Src autoinhibition by Y527 phosphorylation is essential for preventing vascular ballooning. Vascular ballooning was not limited to the venous subtype of ECs (HUVECs), as similar results were obtained when using human aortic ECs (HAECs) (Fig. S1H,I).

In contrast to the bead sprouting assay (Fig. 1E), c-Src-CA microvessels did not display a significant increase in the number of cells per vessel (Fig. 1H). These differences might be due to the length of each assay (7 days for the bead sprouting assay versus 3 days for 3D microvessels). Analysis of proliferation rates in 2D culture using Ki-67 and EdU staining showed that there was no difference in general proliferation indicated by Ki-67, but EdU staining revealed a decrease in cell proliferation (specifically in S-phase) between c-Src-CA mutant and control cells (Fig. S1J–M). This was further supported by our observation that there were no significant changes in the proliferation of c-Src mutant cells in 3D microvessels using Ki-67 and EdU staining (Fig. S2A–D).

Higher magnification analysis of the microvessels revealed that the c-Src-CA vessels had an irregular vessel edge (Fig. 1F,I), but no change in cell size or shape (Fig. 1F′,J,K) compared to control or c-Src-WT cells. Analysis in 2D revealed a significant increase in the cell perimeter and a loss of cell circularity in c-Src-CA cells, but no change in cell size (Fig. S2E–H). This discrepancy in cell shape might be due to morphological alterations of ECs when grown in 2D versus encapsulated 3D settings exposed to flow. We found that a significant percentage of the c-Src-CA vessels lacked cell coverage resulting in gaps in the monolayer (Fig. 1L); however, cells within the vessel still maintained some connections (Fig. 1F″). The monolayer gaps suggested increased cell death; however, analysis of the apoptosis marker cleaved caspase-3 revealed no alterations in apoptosis of the c-Src-CA cells (Fig. S2I,J). Taken together, these results reveal that constitutive activation of c-Src in endothelial cells induces a malformed and perforated vasculature.

### Constitutively active c-Src induces phosphorylation of target proteins at FAs and cell–cell junctions

**Fig. 2.**
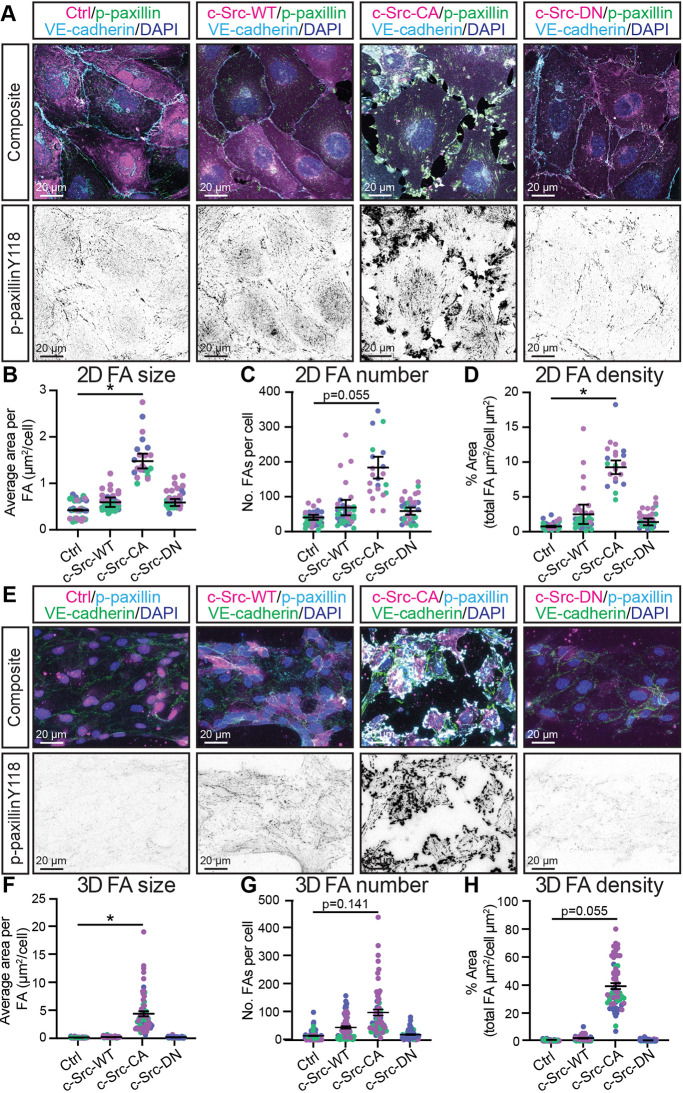
**Constitutively active c-Src induces FA formation and disrupts cell-cell junctions in 2D and 3D.** (A) Representative images of confluent monolayers of HUVECs transduced with mScarlet-tagged c-Src WT, mutants (CA or DN) or control (Ctrl; empty vector mScarlet) (magenta), with immunofluorescence staining performed for FAs [phospho (p)-paxillin Y118; green, shown as individual grey channel in bottom panel], VE-cadherin (cyan) and nuclei (DAPI; blue). (B–D) Quantification of average FA size (B), number (C), and density (D) per cell. *n*=3 independent experiments, 5 images per replicate with each data point representing an average of 3–5 cells per image. (E) Representative images of HUVECs transduced as in A (magenta) and seeded in PDMS microfluidic vessels containing 2.5 mg/ml collagen matrix for 3 days before fixing. Immunofluorescence staining was performed for FAs (p-paxillin Y118; cyan, shown as individual grey channel in bottom panel), VE-cadherin (green) and nuclei (DAPI; blue). (F–H) Quantification of average FA size (F), number (G), and density (H) per cell. *n*=3 independent experiments, 4–5 images per replicate with each data point representing an average of 3–5 cells per image. All data are represented as mean±s.e.m. with individual data point indicated and colours representing independent experiments, and large circles representing average mean per replicate. *P<0.05 (Kruskal–Wallis test with Dunn's multiple comparisons). The mean value of each individual replicate and corresponding s.e.m., instead of all data points separately, was used for statistical analysis.

A loss of c-Src in ECs leads to a reduction in cell–matrix adhesion (Schimmel et al., 2020), and loss of c-Src expression at cell–cell junctions results in reduced VE-cadherin internalisation (Gordon et al., 2016; Sun et al., 2012). We hypothesised that increased c-Src activation would result in an induction of FA and/or VE-cadherin phosphorylation and internalisation. We observed a modest increase in FA size, number and density in c-Src-WT cells grown in 2D, which was further exacerbated by the CA mutation (Fig. 2A–D). Like the 3D microvessels, c-Src-CA induced large gaps in the endothelial monolayer (Fig. 2A), with punctate accumulations of VE-cadherin at sites of cell–cell contact. Furthermore, a significant increase in the phosphorylation of the key FA components paxillin (at Y118) and FAK (at Y576) in c-Src-CA cells was observed in comparison to controls (Fig. S3A,C), in line with previous observations (Ferrando et al., 2012; Martínez et al., 2020). FAK phosphorylation at Y397 was induced (Fig. S3D–G), which is known to be involved in the recruitment and activation of c-Src at FAs (Lietha et al., 2007). This suggests that c-Src acts both up- and down-stream of FAK. Phosphorylation levels of paxillin Y118 and FAK Y576/Y397 in c-Src-DN cells were comparable to those in control cells (Fig. 2A–D; Fig. S3A–G), confirming that the K295 site is essential for the kinase activity of c-Src. Comparing phosphorylation of paxillin Y118 in 2D to the 3D microvessels revealed conservation of the increased FA size and density in c-Src-CA cells compared to control, WT and DN (Fig. 2E–H).

**Fig. 3.**
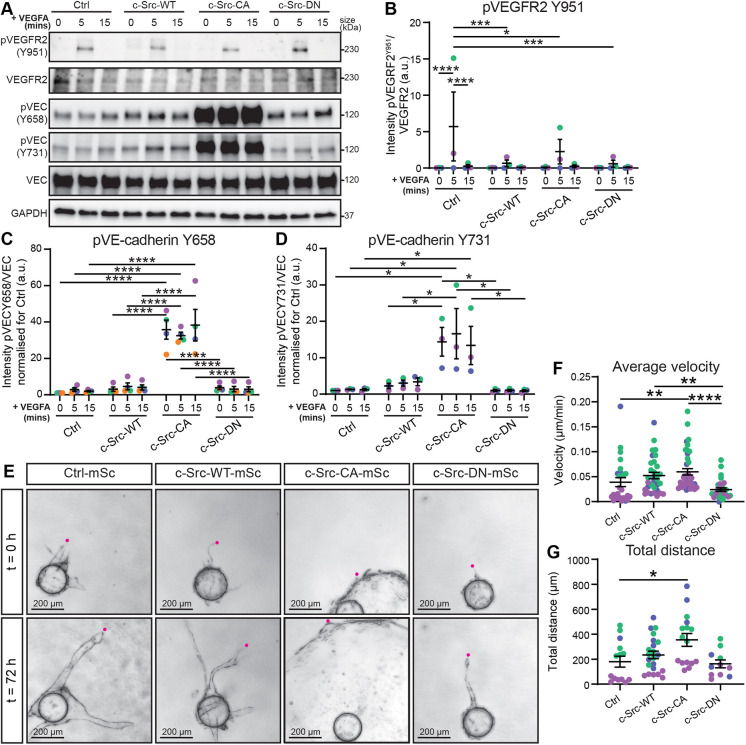
**Constitutively active c-Src increased VE-cadherin phosphorylation and decreases 3D cell migration.** (A) Representative images of western blots of VEGFA-induced VEGFR2 and VE-cadherin phosphorylation in confluent monolayers of HUVECs transduced with mScarlet-tagged c-Src WT, mutants (CA or DN) or control (Ctrl; empty vector mScarlet) and starved overnight in serum-reduced medium before being treated with 100 ng/ml VEGFA for 0 min, 5 min or 15 min. Activation of VEGFR2 and VE-cadherin (VEC) are shown by phosphorylation (p) on indicated tyrosine sites. GAPDH was used as loading control. Full blots with all respective loading controls can be found in Fig. S8. (B) Quantification of VEGFR2 phosphorylation at Tyr(Y)951 confirms receptor activation with a peak at 5 min. Ratio of phosphorylated VEGFR2 (Y951) to total VEGFR2 corrected to loading control (GAPDH). (C) Quantification of VE-cadherin phosphorylation at Y658 relative to total VE-cadherin corrected for loading control (GAPDH) and normalised to 0 min. (D) Quantification of VE-cadherin phosphorylation at Y731 relative to total VE-cadherin corrected for loading control (GAPDH) and normalised to 0 min. *n*=3–4 independent experiments. (E) HUVECs transduced as in A were grown in a 5 mg/ml fibrin gel bead sprouting assay for 5 days before live imaging at 30 min intervals over 72 h (representative images of 0 and 72 h are shown). (F,G) Quantification of total distance from the bead within 72 h (F) and average velocity of the sprouting front (G). *n*=3 independent experiments. using Kruskal–Wallis test with Dunn's multiple comparisons. All data are represented as mean±s.e.m. with individual data point indicated and colours represent independent experiments. *P<0.05, **P<0.01, ***P<0.001, ****P<0.0001 [two-way ANOVA with Tukey's multiple comparisons (B–D) or Kruskal–Wallis test with Dunn's multiple comparisons (F,G)]. a.u., arbitrary units.

To investigate the cause of the loss of endothelial junction integrity (Figs 1F″,L and 2A), we assessed the effect of elevated c-Src activity on VE-cadherin phosphorylation sites (Y658/Y731), which have been previously reported to be linked to VEGFR2-dependent phosphorylation (Giannotta et al., 2013). As expected, c-Src mutants did not alter VEGF-A-induced VEGFR2 phosphorylation at Y951 (Fig. 3A,B), which is a site that has been reported to be upstream of c-Src signalling (Sun et al., 2012). Phosphorylation of VE-cadherin at Y658 or Y731 was not significantly altered by c-Src-WT or c-Src-DN compared to control, whereas c-Src-CA cells displayed a robust increase of VE-cadherin phosphorylation independently of VEGF-A stimulation (Fig. 3A,C,D). Therefore, reduction of EC–cell contacts in c-Src-CA cells might be due to elevated VE-cadherin phosphorylation and subsequent internalisation.

We have previously identified that loss of FAs in endothelial cells results in a highly unstable vasculature due to the inability of cells to form functional contacts with their surrounding matrix (Schimmel et al., 2020). However, although cells could not move in a co-ordinated manner, their velocity was unchanged. Thus, we next assessed the effect of c-Src mutants on EC migration in 2D and 3D. We found that c-Src-CA significantly reduced cell migration velocity and distance in 2D, whereas migration remained unaltered in cells expressing c-Src-WT or c-Src-DN (Fig. S4A–C; Movie 3). The reduction in migration velocity in c-Src-CA cells coincides with an increase in FA size, number and density (Fig. 2A–D). This suggests that the reduction of migration velocity is due to increased cellular adhesion via FAs. As reduction in cell–cell junction integrity has been shown to increase migratory capacity and sprouting angiogenesis (Bentley et al., 2014), our data suggest that a balanced control of both cell–matrix and cell–cell junctions is essential for mediating migration. When migratory capacity was assessed in 3D over 3 days (Fig. 3E–G; Movie 4), the average velocity of c-Src-CA cells (Fig. 3F) and the total distance travelled from the bead by c-Src-CA cells (Fig. 3G) was increased compared to that seen for control. However, the differences in migration capacity of c-Src-CA cells observed in 2D (Fig. S4) and 3D (Fig. 3) are likely due to the intricate challenges posed by migration through a mesh network of extracellular molecules, wherein cells exhibit the ability to move in a 3D manner rather than adhering to linear movement patterns typically seen on matrix-coated glass surfaces (Gordon et al., 2020).

### c-Src activation induces local degradation of the surrounding ECM

Our data demonstrated that c-Src-CA induced elevated levels of paxillin (Y118), FAK (Y576) and VE-cadherin (Y658, Y731) phosphorylation and reduced endothelial cell–cell contacts. However, alterations in cell–matrix adhesion and cell–cell adhesion alone are not sufficient to explain the rapid expansion of c-Src-CA cells into the 3D fibrin (Fig. 1A) or collagen (Fig. 1F) matrix. FAs are localised sites of exocytosis in ECs (Kawakami et al., 2001), allowing secretion of proteases to subsequently remodel their surrounding environment in non-ECs (Nolte et al., 2020). Furthermore, unphosphorylated paxillin is known to be required for fibronectin fibrillogenesis (Zaidel-Bar et al., 2007). Therefore, we hypothesised that upon elevation of c-Src activation, which significantly increases phospho-paxillin Y118 and FA number and size, fibronectin fibril assembly and ECM degradation might be altered.

**Fig. 4.**
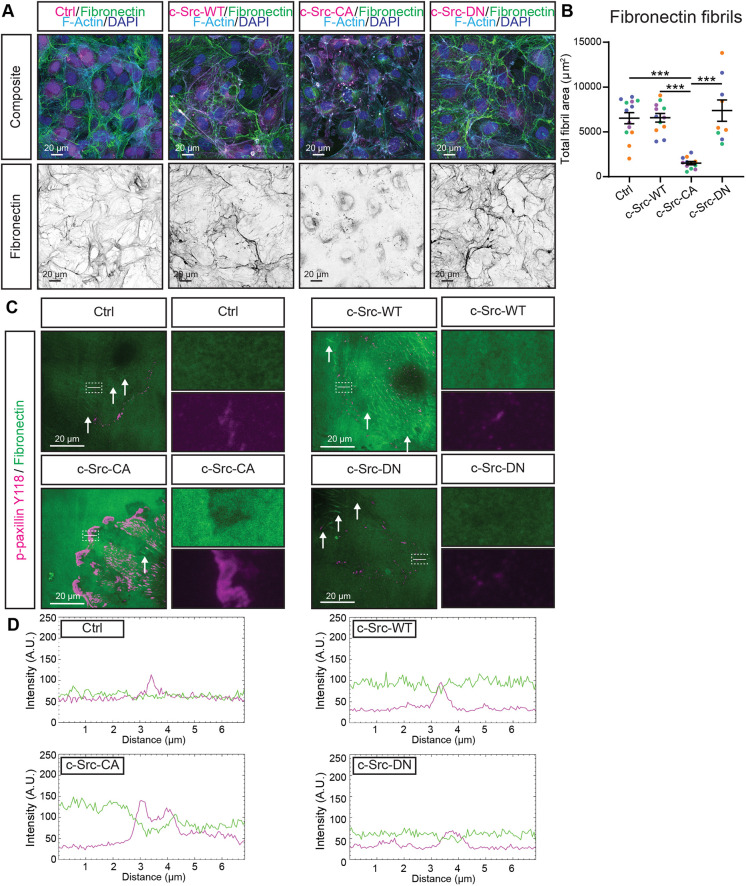
**Constitutively active c-Src disrupts fibrillogenesis and degrades fibronectin locally at FAs.** (A) Representative images of HUVECs transduced with mScarlet-tagged c-Src WT, mutants (CA or DN) or control (Ctrl; empty vector mScarlet) (magenta) grown on fibronectin-coated glass for 24 h before fixing. Immunofluorescence staining was performed for fibronectin (green, shown as individual grey channel in bottom panel), F-actin (phalloidin; cyan) and nuclei (DAPI; blue). (B) Quantification of total fibronectin fibril area per image. *n*=4 independent experiments, 3 images per replicate. (C) Representative images of total internal reflection fluorescence (TIRF) microscopy of sub-confluent mScarlet-tagged c-Src mutant HUVECs grown on fibronectin for 4 h before fixing. Immunofluorescence staining was performed for fibronectin (green) and focal adhesions (p-paxillin Y118; magenta). White dashed box indicates region of higher magnification, single channel images are shown next to the merge, white lines indicate area used to generate intensity plots, white arrows indicate fibronectin fibrils. (D) Intensity plots of areas of interest indicated by white lines in C to demonstrate a decrease in fibronectin (green) intensity at sites of increased focal adhesion signal (magenta). All lines are 7 µm and traverse through a focal adhesion. All data are represented as mean±s.e.m. with individual data points indicated and colours representing independent experiments. ***P<0.001, ****P<0.0001 (Kruskal–Wallis test with Dunn's multiple comparisons). Images in C and D are representative of three independent experiments, 3–5 images per replicate.

We assessed the deposition of fibronectin fibrils over a time course ranging from 4–48 h in c-Src mutant cells grown on uncoated glass coverslips. We found that c-Src-CA cells did not assemble fibrils over time (Fig. S5A,B) in contrast to control, c-Src-WT and c-Src-DN cells, which displayed elevated fibril assembly (48 h versus 4 h). To ascertain whether this was due to a reduction in fibronectin deposition or increased degradation, we grew ECs on fibronectin-coated coverslips for 24 h. This resulted in a significant decrease in the fibril area underneath c-Src-CA cells, which was rescued by the c-Src-DN mutation (Fig. 4A,B). In addition to increased fibronectin degradation by c-Src-CA cells, we identified specific local fibronectin degradation at the sites of enlarged FAs in c-Src-CA cells via total internal reflection microscopy (Fig. 4C,D). The localised degradation of fibronectin at FAs was also verified in actively migrating ECs (Fig. S5C,D). These results suggest that FAs within ECs act as sites of local secretion of proteases to degrade surrounding ECM components during cell migration.

**Fig. 5.**
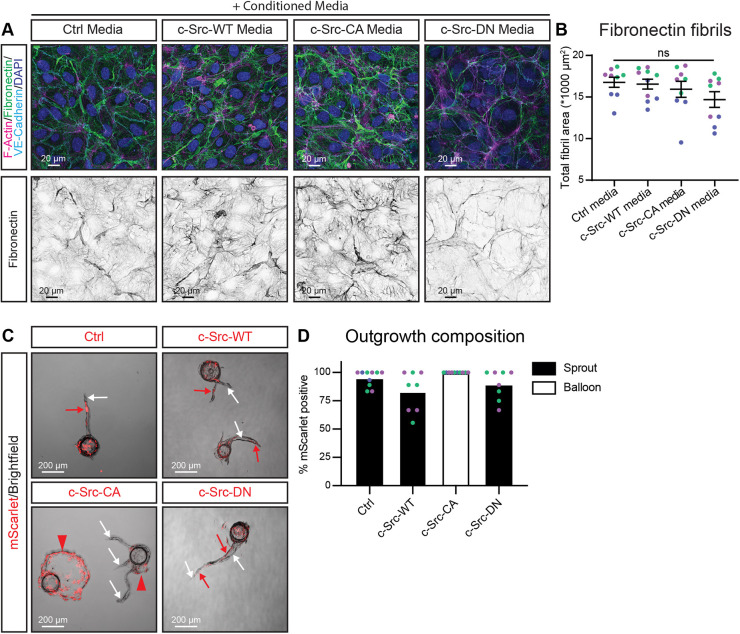
**c-Src-dependent fibronectin degradation is mediated by insoluble factors.** (A) Representative images of HUVECs grown on fibronectin-coated glass, treated with conditioned medium from HUVECs transduced with c-Src WT, mutants (CA or DN) or control (Ctrl; empty vector mScarlet). Cells were fixed and immunofluorescence staining was performed for F-actin (phalloidin; magenta), fibronectin (green), VE-cadherin (cyan) and nuclei (DAPI; blue). Bottom panel shows individual fibronectin channel in grey. (B) Quantification of total fibronectin fibril area per image upon treatment with the conditioned medium from the transduced c-Src cells. *n*=3 independent experiments with 3 images per replicate. (C) Representative images of mixed bead sprouting assay for untransduced (brightfield) and mScarlet-tagged c-Src-transduced (red) HUVECs grown in a 5 mg/ml fibrin gel bead sprouting assay for 7 days before imaging. (D) Quantification of the percentage of outgrowth area (sprouts, black bars; balloons, white bars) positive for mScarlet. *n*=3 independent experiments for Ctrl and c-Src-CA, *n*=2 independent experiments for c-Src-WT and c-Src-DN all with 2–4 images per replicate. All data are represented as mean (±s.e.m. for B) with individual data point indicated and colours represent independent experiments. ns, not significant (Kruskal–Wallis test with Dunn's multiple comparisons). Statistics not performed for D.

As proteases can be membrane inserted or secreted, we next addressed whether the cells were releasing soluble proteases. We added conditioned media from control, c-Src-WT, c-Src-CA and c-Src-DN cells and applied that to wild-type ECs. As wild-type cells were still able to remodel fibronectin into fibrils upon addition of conditioned medium obtained from c-Src-CA cells (Fig. 5A,B), we conclude that the proteases secreted at FAs were not soluble and are likely tethered to the cell membrane. This was further evidenced by mixing unlabelled wild-type ECs with mScarlet-labelled control, c-Src-WT, c-Src-CA and c-Src-DN cells in a bead sprouting assay. Control, c-Src-WT and c-Src-DN cells formed sprouts containing both wild-type and mutant cells (Fig. 5C,D). However, c-Src-CA cells exclusively formed balloons (red arrowheads), whereas wild-type cells coated on the same bead maintained their ability to form sprouts (white arrows) (Fig. 5C). Taken together, these results reveal that proteases produced by c-Src-CA cells are locally secreted at FAs but are membrane bound.

### Inhibition of MMPs rescues vascular ballooning induced by constitutively active c-Src

**Fig. 6.**
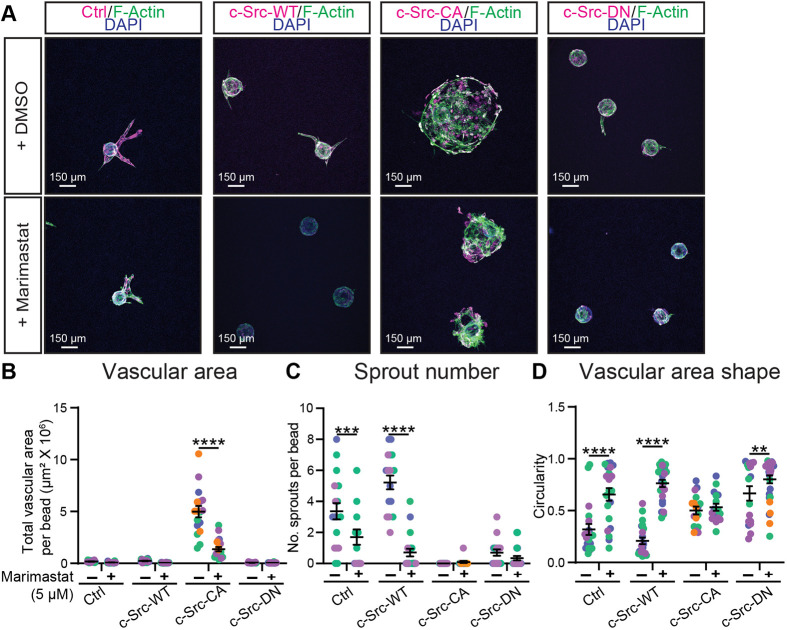
**The broad-spectrum MMP inhibitor Marimastat rescues vascular ballooning in constitutively active c-Src in 3D bead sprouting.** (A) Representative images of HUVECs transduced with c-Src WT, mutants (CA or DN) or control (Ctrl; empty vector mScarlet) (magenta) grown in a 5 mg/ml fibrin gel bead sprouting assay for 7 days and treated every 2 days with DMSO vehicle control or 5 µM Marimastat before fixing. Immunofluorescence staining was performed for F-actin (phalloidin; green) and nuclei (DAPI; blue). Quantification of sprout parameters of vascular area (B), number of sprouts per bead (C), and shape of vascular area (D). *n*=3 independent experiments. All data are represented as mean±s.e.m. with individual data points indicated and colours representing independent experiments. **P<0.01, ***P<0.001, ****P<0.0001 (two-way ANOVA with Sidak's multiple comparisons test).

MMPs degrade various proteins within the ECM and play a role in a range of vascular processes, including angiogenesis, morphogenesis and wound repair (Wang and Khalil, 2018). In non-endothelial cells, FAs have been shown to be sites of MMP secretion, mediating FA turnover and allowing for cell migration (Stehbens et al., 2014). This suggests that excessive ECM breakdown in c-Src-CA cells mediated by elevated MMP activity at FAs may result in rapid vascular ballooning. To test whether inhibition of MMP activity could rescue the c-Src-CA phenotype, we added the potent, broad-spectrum MMP inhibitor Marimastat, which has been utilised in a number of oncology clinical trials (Rasmussen and Mccann, 1997; Thomas and Steward, 2000). Addition of Marimastat to ECs grown in a 2D monolayer was able to rescue fibronectin degradation in c-Src-CA cells (Fig. S6A,B). However, c-Src-CA cells still maintained increased number and size of FAs compared to control, c-Src-WT or c-Src-DN cells (Fig. S6A,C–E). Treatment of ECs with Marimastat in a fibrin bead sprouting assay resulted in significant reduction of the ballooning morphology observed in the c-Src-CA cells (Fig. 6A–D). c-Src-CA cells retained a significant increase in vascular area when compared to control or c-Src-WT cells, indicating only a partial rescue, likely due to drug addition 24 h after seeding (Fig. 6A; Table S1). MMP inhibition also decreased the sprouting ability of control and c-Src-WT cells, with a decrease in the sprout number and increased circularity (due to decreased number of sprout protrusions) (Fig. 6A,C,D).

**Fig. 7.**
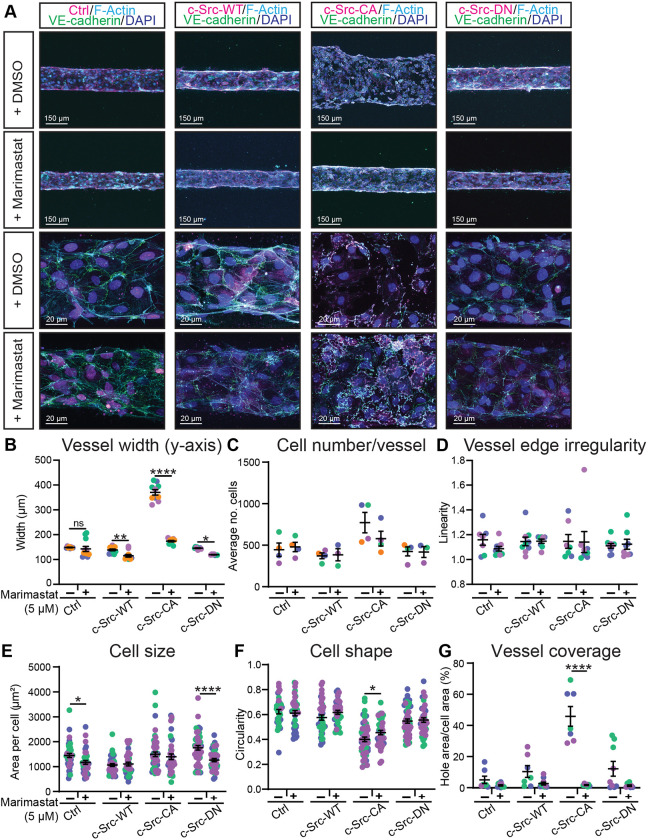
**The broad-spectrum MMP inhibitor Marimastat rescues vascular ballooning in constitutively active c-Src in 3D microvessels.** (A) Representative images of of HUVECs transduced with c-Src WT, mutants (CA or DN) or control (Ctrl; empty vector mScarlet) (magenta) seeded in PDMS microfluidic vessels containing 2.5 mg/ml collagen matrix for 3 days and treated daily with DMSO vehicle control or 5 µM Marimastat before fixing. Immunofluorescence staining was performed for VE-cadherin (green), F-actin (phalloidin; cyan) and nuclei (DAPI; blue). (B–D) Low magnification images (top rows in A) were used to quantify vessel width (B), number of cells per vessel (C) and vessel edge irregularity (D). *n*=3–5 independent experiments, 3 vessels per replicate with each data point representing an average of 3–5 measurements per vessel. (E–G) High magnification images (bottom rows in A) were used to quantify individual cell morphology within microvessels as cell size (E), cell shape (F) and vessel coverage as a percentage of total vessel area (G). *n*=3–5 independent experiments, 3–5 images per replicate with each data point representing an average of 1–5 cells per image. All data are represented as mean±s.e.m. with individual data points indicated and colours representing independent experiments. *P<0.05, **P<0.01, ***P<0.001, ****P<0.0001 (two-way ANOVA with Sidak's multiple comparisons test). The mean value of each individual replicate and corresponding s.e.m., instead of all data points separately, was used for statistical analysis in E and F.

To investigate whether MMP inhibition could also rescue the dysfunctional vasculature induced by c-Src-CA cells in a collagen matrix, 3D microvessels were treated with Marimastat. In control cells, Marimastat treatment had no effect on vessel width, vessel edge irregularity or vessel coverage, nor on cell size, shape and number (Fig. 7A–G), suggesting that in a non-angiogenic setting, Marimastat does not adversely affect vessel morphology. In agreement with the results observed in the fibrin bead sprouting assay (Fig. 6), treatment of 3D microvessels with Marimastat resulted in a partial rescue of the increase in vessel width induced by c-Src-CA cells (Fig. 7A,B). This partial rescue of c-Src-CA cells by Marimastat treatment was confirmed in HAECs (Fig. S7), confirming conservation of the mechanism in both venous and arterial ECs. Although large FAs were still detected in c-Src-CA cells treated with Marimastat (Fig. S6C), the loss of c-Src-CA cell coverage of the vessel was restored (Fig. 7A,G). Taken together, these results reveal that elevated c-Src activation induces large FAs, inducing enhanced local secretion of MMPs to degrade the ECM and subsequent vascular malformations.

## DISCUSSION

Our investigation into the impact of a constitutively active variant of c-Src on endothelial dynamics has unveiled a pivotal role for c-Src in orchestrating FA formation, steering the course of MMP secretion and activity precisely at the nexus of cell–matrix adhesion. Here, we show that strategically confined MMP-mediated matrix degradation at FAs leads to a rapid and aberrant dilation of the vascular lumen within the context of 3D environments. However, this dilation can be partially counteracted by administering the broad-spectrum MMP inhibitor Marimastat. These findings reveal a novel function of FAs as targeted loci of protease secretion within ECs, generating an environment that is conducive to promoting cellular migration in a complex 3D matrix.

The regulatory influence of SFKs on EC behaviour and vascular adhesion has been extensively investigated across diverse contexts. The existence of structural similarities among c-Src, Yes and Fyn (Sato et al., 2009) coupled with the limited availability of tools for precise interrogation of the distinct roles of each SFK member within the endothelium, has presented considerable challenges. The recent adoption of conditional knockout mice with endothelial-specific modifications has yielded valuable insights into the distinct control exerted by individual SFK members over various signalling outputs and resultant biological phenomena. In the context of developing retinal tissue, we have found that loss of c-Src in ECs results in impaired cell–matrix adhesion without gross alterations in VE-cadherin localisation or phosphorylation (Schimmel et al., 2020). In contrast, loss of Yes in ECs results in reduced VE-cadherin phosphorylation and endocytosis (Jin et al., 2022). Here, we clearly demonstrate that induction of c-Src activity results in elevated VE-cadherin phosphorylation, an occurrence decoupled from growth factor instigation or induction of fluid forces. These findings conclusively affirm the capability of c-Src to phosphorylate VE-cadherin. It should be noted that the predominant SFK responsible for VE-cadherin phosphorylation is subject to contextual variability; c-Src appears to orchestrate phosphorylation and consequent permeability downstream of endothelial nitric oxide synthase (eNOS) activity (Ninchoji et al., 2021), whereas Yes likely takes on a primary role in this process in response to fluid shear stress (Jin et al., 2022).

The controlled expansion of vascular lumens is primarily mediated through Rho GTPase-dependent regulation of actin contractility. Ras-interacting protein 1 (Rasip1) plays a role in enhancing the activity of Cdc42 and Rac1 while concurrently inhibiting RhoA (Xu et al., 2011). Together with ArhGAP29, this can further block Rho associated kinase (ROCK) signalling, which normally acts to phosphorylate myosin light chain (MLC) and regulate F-actin contractility (Post et al., 2015; Xu et al., 2011). The inhibition of either RhoA or ROCK proteins leads to lumen expansion due to the disruption of actin contractility, whereas the absence of Rasip1 results in narrower lumens in developing vasculature (Barry et al., 2016; Koo et al., 2016). Interestingly, c-Src has been shown to disrupt RhoA signalling through p190 RhoGAP (also known as ArhGAP35), leading to disruption of FAs and increased motility (Chang et al., 1995). Additionally, c-Src can counteract ROCK signalling, promoting vessel stability (Im and Kazlauskas, 2007). This stands in contrast to the phenotypes observed here when c-Src is activated, namely large FAs and destabilised vasculature. This discrepancy might arise from differences in the extent of c-Src activation in these models, in agreement with requirement for a controlled balance in pMLC levels (Schnellmann et al., 2022). Phosphoproteomic screening for c-Src substrates in non-endothelial cells has identified numerous candidates associated with actin remodelling and adhesion, such as filamin B, tensin 1 and p130-Cas (also known as BCAR1) (Ferrando et al., 2012). Therefore, c-Src clearly possesses the capability to promote actin-dependent processes, potentially inducing heightened tension at the membrane, which could lead to alterations in the linear distribution of VE-cadherin.

Whereas cell–matrix and cell–cell adhesions were traditionally considered distinct membrane components, it is now becoming clear that cell–cell junctions and FAs are intricately associated (Canel et al., 2013; Mui et al., 2016; Pulous et al., 2019; Schwartz and Desimone, 2008; Weber et al., 2011). Significant crosstalk between both adhesion sites occurs via reciprocal binding to the actin cytoskeleton in addition to binding similar effector proteins like c-Src and FAK (Canel et al., 2013). Disruption of FA components, such as talin proteins, results in impaired stability of the vascular bed and altered VE-cadherin organisation at junctions (Chau et al., 2022; Pulous et al., 2019), whereas stabilisation of the cytoskeleton upon loss of talin restores VE-cadherin organisation and vascular stability, demonstrating the actin-mediated crosstalk between the two adhesion compartments (Chau et al., 2022). As c-Src has been found to regulate both FAs and cell–cell junctions, it possesses the capacity to fine-tune EC migratory behaviour. Besides the direct interaction with FAs and cell–cell junctions, the above discussed effects and potential control of the actin cytoskeleton by c-Src would enable regulation of the crosstalk between the adhesion compartments. This adds an additional layer of complexity to the molecular mechanism of c-Src-mediated control of cell adhesion and migration. The precise mechanisms governing the assembly and disassembly of both FAs and cell–cell junctions by the constituents of these adhesion complexes, including c-Src, FAK, paxillin, talin, vinculin and the actin cytoskeleton, represent a captivating area for future study.

In addition to mediating cell–matrix adhesion and crosstalk to cell–cell junctions, FAs have been reported to function as exocytosis hotspots to mediate local ECM degradation and FA detachment, which is essential for migration (Stehbens et al., 2014; Zaidel-Bar et al., 2007). Our results confirm that increased FA size and number, due to increased phosphorylation of complex constituents, induces MMP-dependent ECM degradation. Active c-Src has been implicated in the co-ordination between the tubulin cytoskeleton and the apical membrane, directing polarised fusion events (Kim et al., 2017). In non-endothelial cells, microtubules guide the trafficking of MMPs specifically to FAs in order to promote FA turnover. Our data suggests this phenomenon is conserved in the endothelium, where large FAs act as docking sites for microtubule-mediated MMP exocytosis, initiating local breakdown of the surrounding matrix.

In endothelial cells, c-Src is known to induce phosphorylation and expression of MT1-MMP (Nyalendo et al., 2007), a membrane-bound MMP that has been shown to enhance invasion and migration of ECs during angiogenesis (Bordeleau et al., 2017; Jeong et al., 1999). Therefore, it is likely that MT1-MMP trafficking to FAs is altered in our models. Interestingly, this is not the first instance of localised secretion occurring at sites of cell–matrix adhesion in the endothelium, as exemplified by the FA protein zyxin, which is known to facilitate the secretion of von Willebrand factor (vWF) from Weibel–Palade bodies (WPBs) (Han et al., 2017). Zyxin mediates the dynamic reorganisation of actin filaments around WPBs at sites of exocytosis, promoting fusion and release of vWF. This illustrates that FAs act as microdomains for exocytosis, influencing various vascular processes. However, whether c-Src-induced exocytosis can similarly regulate the release of vWF and promote thrombi formation remains unknown.

Growing cells within a 3D network offers the advantage of applying mechanical forces, a crucial aspect when investigating processes influenced by mechanics, such as cell–matrix adhesion. Moreover, assessing cellular phenotypes within this 3D network permits the analysis of mechanical forces, including fluid flow, mechanical stiffness and stretch (Gordon et al., 2020; Wang et al., 2023). Notably, we consistently observed alterations in FAs across various 2D and 3D models, and the application of Marimastat effectively rescued matrix degradation, regardless of the environmental context. In our study, we observed similar phenotypes when cells were grown in collagen or fibrin, or with or without flow. Thus, in our models, vascular expansion appears to be independent of the specific ECM type surrounding the cells and is not contingent upon fluid flow. However, it is important to note that c-Src is a well-known mechanosensory protein (Koudelková et al., 2021) and can regulate how the endothelium responds to changes in fluid flow (Caolo et al., 2018; Conway et al., 2013; Coon et al., 2015; Orsenigo et al., 2012; Vion et al., 2021), stiffness (Wang et al., 2019) and stretch (Gawlak et al., 2016; Naruse et al., 1998; Tian et al., 2016). Alterations in these mechanical forces could conceivably lead to changes in c-Src activity, potentially comparable to the levels observed in c-Src-CA cells, thereby triggering the induction of vascular malformations.

The interactions between the vasculature and the ECM are associated with a range of disease states. In the process of luminal expansion in vascular malformations and arteriovenous malformations (AVMs), vessels need to overcome the mechanical ECM barrier to allow for vascular remodelling and infiltration into a 3D space. Interestingly, pre-clinical models of hereditary haemorrhagic telangiectasia (HHT) exhibit elevated integrin and FA activity (Park et al., 2021) in the first report linking endothelial cell–ECM interaction to vascular malformations. The requirement of elevated MMP secretion in diseases such as HHT is evidenced by the fact patients with AVMs show elevated MMP9 and MMP2 expression in plasma and tissue samples (Liu et al., 2035; Wei et al., 2016). Our data suggest that heightened c-Src activity causes MMP-induced ECM degradation to enable expansion of vascular malformations. Whether c-Src activity is induced in HHT or in endothelial cells within AVMs remains to be determined. Notably, ECM degradation allows endothelial cells to create new blood vessels in stiff environments. Increased ECM stiffness, which is common in conditions like cancer, promotes angiogenesis and disrupts blood vessel barriers, elevating the risk of metastasis (Bordeleau et al., 2017; Fenner et al., 2014; Setargew et al., 2021). High levels of MMPs correlate with poor prognosis in cancer patients, and MMPs have been investigated for their involvement in mediating growth of the tumour vasculature (Senger and Davis, 2011; Têtu et al., 2006). Although it is known that c-Src expression is increased in human cancers and c-Src is a well-known promotor of tumour angiogenesis (Eliceiri et al., 1999; Simatou et al., 2020), the specific mechanisms by which changes in ECM stiffness activate endothelial c-Src to induce MMP secretion remain unknown.

In conclusion, our research has yielded compelling evidence underscoring the importance of finely tuned regulation of cell–matrix interactions in the establishment of a robust and functional vascular system. Through our investigations, we have showcased that the signalling processes intrinsic to ECs demand meticulous control. Specifically, our findings have highlighted the consequences of perturbations in c-Src activity levels, with both the loss of c-Src activity, as documented by Schimmel (Schimmel et al., 2020), and the hyperactivation of c-Src, as documented here, leading to the formation of aberrant vascular networks. This newfound understanding opens promising avenues for therapeutic intervention in diseases characterised by irregular vasculature. One such strategy revolves around the targeted modulation of endothelial-derived proteases, aimed at curtailing vascular invasion and uncontrolled expansion, which holds potential for ameliorating conditions associated with malformed vasculature.

## MATERIALS AND METHODS

### Antibodies and dyes

The following antibodies and dyes were used: rabbit anti-phospho-c-Src (Y416) (Invitrogen, 44660G, 1:100 for immunocytochemistry (ICC)], rabbit anti-phospho-VE-cadherin (Y658) [Invitrogen, 44-1144G, 1:1000 for western blotting (WB)], rabbit anti- phospho-VE-cadherin (Y731) (Invitrogen, 44-1145G, 1:500 for WB), rabbit anti-phospho-paxillin (Y118) (Invitrogen, 44-722G, 1:1000 for WB, 1:200 for ICC), mouse anti-c-Src GD11 (Millipore, 05-184, 1:1000 for WB, 1:200 for ICC), goat anti-VE-cadherin (R&D systems, AF938, 1:2000 for WB), goat anti-VEGFR2 (R&D systems, AF357, 1:1000 for WB), mouse anti-VE-cadherin-Alexa Fluor 647 (BD Biosciences, 561567, 1:250 for ICC), mouse anti-phospho-FAK (Y397) (BD Biosciences, 611806, 1:200 for ICC, 1:1000 for WB), mouse anti-fibronectin (BD Biosciences, 610077, 1:200 for ICC), rabbit anti-phosho-paxillin (Y118) (Abcam, AB4833, 1:100 for ICC), rabbit anti-Ki-67 (Abcam, ab15580, 1:200 for ICC), rabbit anti-paxillin (Cell Signaling, 2542, 1:1000 for WB), rabbit anti-phospho-FAK (Y576) (Cell Signaling, 3281, 1:1000 for WB), rabbit anti- phospho-VEGFR2 (Y951) (Cell Signaling, 2471, 1:1000 for WB), rabbit anti-GAPDH (Cell Signaling, 2118, 1:5000 for WB), rabbit anti-DLL4 (Cell Signaling, 2589, 1:500 for WB), mouse anti-FAK (Santa Cruz Biotechnology, sc-271126, 1:1000 for WB), phalloidin (conjugated to Alexa Fluor 488 and 670, Cytoskeleton Jomar, PHDG1-A, PHDN1-A, 1:500 for ICC), rabbit anti-cleaved caspase 3 (D175) (Cell Signaling, 1:200 for ICC) and the Click-iT EdU Cell Proliferation kit Alexa Fluor 647 dye (Invitrogen, C10340, 10 µM for ICC).

Secondary antibodies conjugated to Alexa Fluor 488, Alexa Fluor 555 or Alexa Fluor 647 for immunofluorescence staining were obtained from Invitrogen and used at 1:400 for ICC. Horseradish peroxidase (HRP)-conjugated secondary antibodies were obtained from Thermo Fisher Scientific and used at 1:5000 for western blotting.

### Cell culture

Human umbilical vein endothelial cells (HUVECs) from a single donor (CC-2935 Lonza), which had been authenticated and tested for contamination by Lonza, were cultured in Endothelial Basal Media-Plus supplemented with SingleQuots Bullet Kit (EGM-Plus, CC-5035 Lonza) until passage 2–3 for 3D cultures and passage 3–7 for 2D cultures. Human aortic endothelial cells (HAECs) from a single donor (CC-2535 Lonza), which had been authenticated and tested for contamination by Lonza, were cultured in Endothelial Basal Media-2 supplemented with SingleQuots Bullet Kit (EGM-2, CC-3162 Lonza) until passage 2–3 for 3D cultures. Human lung fibroblasts (NHLFs, CC-2512 Lonza) and human embryonic kidney (HEK)-293T cells (12022001 CellBank), authenticated and tested for contamination upon receipt, were cultured in Dulbecco's modified Eagle's medium (DMEM) with L-glutamine and sodium pyruvate (Invitrogen), containing 10% (v/v) heat-inactivated FBS and 100 U/ml penicillin and streptomycin (Life Technologies, Australia). All cells were kept at 37°C and 5% CO_2_.

### Generation of c-Src mutants

The pLV-CMV-IRIS-PURO-c-Src-mScarlet plasmid (kindly provided by Jaap van Buul, University of Amsterdam, The Netherlands) was used to the generation of Y527F and Y527F/K295R mutants via QuickChange II site-directed mutagenesis kit (Agilent Technologies, 200523) according to the manufacturer's protocol. Generation of the c-Src-Y527F mutant used the forward primer 5′-TCGACAGAGCCCCAGTTCCAGCCTGGAGAGAAC-3′ and the reverse primer 5′-GTTCTCTCCAGGCTGGAACTGGGGCTCTGTCGA-3′. Generation of the c-Src-K295R mutant used the forward primer 5′-CCAGAGTGGCCATAAGGACTCTGAAGCCCGG-3′ and the reverse primer 5′-CCGGGCTTCAGAGTCCTTATGGCCACTCTGG-3′.

### Lentiviral transduction

Lentivirus constructs (used at 136 ng/cm^2^) were packaged into lentiviral particles in HEK-293T cells by co-transfection of third-generation lentiviral packaging plasmids (68 ng/cm^2^ pMDL/pRRE, 34 ng/cm^2^ pRSV-REV, 41 ng/cm^2^ pMD2.G, kindly provided by Lena Claesson-Welsh, Uppsala University, Sweden) with 0.84 µl/cm^2^ PEI 2500 (Polyplus at 1 mg/ml) in OptiMEM (Gibco) according to the manufacturer's protocol. Supernatant containing lentivirus was harvested (at 1500 ***g*** for 45 min) at 48 and 72 h after transfection, filtered through a 0.45 µm filter and concentrated using Lenti-X (Scientifix, 631231). For lentiviral transduction, HUVECs were incubated with concentrated lentivirus, selected with puromycin (1.5 mg/ml) after 24 h and used for assays after 72 h.

### Bead sprouting assay

Fibrin gel bead sprouting assays were performed as described previously (Nakatsu et al., 2007). Briefly, HUVEC were coated onto Cytodex 3 microcarrier beads (Sigma-Aldrich) overnight. Coated microcarrier beads were embedded in a fibrin gel [fibrinogen (5 mg/ml, Sigma-Aldrich, F8630) and aprotinin (50 µg/ml, Sigma-Aldrich, A3428)] in EBM-Plus (Lonza, CC-5036) supplemented with 2% fetal bovine serum (Thermo Fisher Scientific, 10099141). Fibrinogen solution containing the microcarrier beads was clotted using 1 U of thrombin (Sigma-Aldrich, T9549/T4393) for 30 min at 37°C in a 24 well ibiTreat U-plate (Ibidi, 82406). Fibroblasts (NHLFs; 20,000 cells per well) were added on top of the fibrin gel in EGM-Plus with 100 ng/ml VEGF-A (Thermo Fischer Scientific, PHD9391) and medium was refreshed every other day.

Live imaging was performed between days 3–7. Z-stacks were acquired every 30 min for 72 h at 37°C and 5% CO_2_ using a Zeiss Axio Observer 7 inverted widefield microscope.

After 7 days, sprouts were fixed and stained for ICC. Fibroblasts were removed using Trypsin and gels were thoroughly washed with PBS^+/+^ (PBS supplemented with 1 mM CaCl_2_ and 0.5 mM MgCl_2_) before fixing with 4% paraformaldehyde (PFA) for 10 min, permeabilising with 0.5% Triton X-100 for 5 min, and blocking with 5% BSA in PBS^+/+^ for 2 h at room temperature on a shaker. Primary and secondary antibodies were incubated overnight at 4°C in 5% BSA in PBS^+/+^. Sprouts were imaged with a Zeiss LSM 710 Confocal microscope with a Plan Apo 10×/0.45 objective.

### Microfluidic devices

Microfluidic devices were prepared as described previously (Polacheck et al., 2019) using 2.5 mg/ml collagen I (rat tail, R&D Systems) as ECM. In brief, PDMS (Sylgard 184) was cast in moulds, degassed for 2 h and cured overnight at 65°C. PDMS gels were cut out, and ports were punched and bonded to plasma-treated glass coverslips. The ECM channel was treated with 0.01% poly-L-lysine for 4 h followed by 1.1% glutaraldehyde for 15 min and washed overnight in MQ. Steel acupuncture needles (diameter of 200 µm) were inserted before UV sterilisation and insertion of collagen I ECM. Channels were seeded with HUVECs or HAECs at 10^6^ cells per ml and maintained under oscillatory, gravity-driven flow by a lab rocker (>3 dynes/cm^2^) for 3 days at 37°C with 5% CO_2_. Medium was refreshed daily.

Microvessels were fixed subsequently with 1% PFA containing 0.05% Triton X-100 for 90 s, followed by 4% PFA for 15 min, and permeabilised in 0.5% Triton X-100 for 15 min all at 37°C. Microvessels were blocked in 2% BSA in PBS^+/+^ for 4 h at room temperature. Primary antibodies were incubated overnight at 4°C in 2% BSA in PBS^+/+^. Microvessel *Z*-stack images were acquired on a Zeiss LSM 880 Confocal microscope with 10×/0.45 Air or 40×/1.2 water immersion objectives.

### Western blotting

Cells were washed once with PBS^+/+^ and lysed in SDS-sample buffer containing 100 mM DTT and boiled at 95°C for 10 min to denature proteins. Proteins were separated by SDS-PAGE on 4–15% gradient gels (Mini-PROTEAN Precast gels, Bio-Rad) in running buffer (200 mM glycine, 25 mM Tris-HCl pH 8.6, 0.1% SDS) and transferred onto nitrocellulose membrane (Bio-Rad, 1620112) in blot buffer (48 nM Tris-HCl, 39 nM glycine, 0.04% SDS and 20% methanol). Membranes were blocked in 5% (w/v) BSA (Sigma) in Tris-buffered saline with 0.1% Tween 20 (TBST) for 30 min before incubating in primary antibodies overnight at 4°C followed by secondary antibodies linked to HRP (Invitrogen) for 1 h at room temperature. Between each step, the membranes were washed three times for 10 min in TBST. The HRP signals were visualised by enhanced chemiluminescence (ECL; Bio-Rad) and imaged with a Chemidoc (Bio-Rad). Images were analysed using FIJI software, measuring signal intensity, and adjusted to the loading control. Phosphorylated proteins were adjusted to the relative total protein. Full scans of the membranes can be found in Fig. S8.

### Immunocytochemistry

For 2D immunofluorescence staining, HUVECs were cultured on 12 mm glass coverslips, or in 35 mm glass bottom dish (MatTek, P35G-1.5-14-C), coated with 5 µg/ml fibronectin (Sigma). Cells were washed with PBS^+/+^, fixed in 4% PFA for 10 min, and blocked/permeabilised for 30 min in 3% BSA, 0.3% Triton X-100 in PBS^+/+^. Primary antibodies were incubated with cells for 60 min at room temperature in 1.5% BSA in PBS^+/+^, washed three times in PBS^+/+^, and incubated in secondary antibody for 60 min at room temperature in 1.5% BSA in PBS^+/+^ before mounting in ProlongGold containing DAPI (Cell Signaling Technologies). Imaging was performed on a Zeiss LSM 710 Confocal microscope with 10×/0.45 air, 40×/1.1 water immersion or 63×/1.15 water immersion objectives or on a Zeiss LSM 880 confocal microscope with 10×/0.45 Air, 40×/1.2 water immersion, 40×/1.3 oil immersion or 63×/1.4 oil immersion objectives.

### Conditioned media

Puromycin selected HUVECs transduced with control or c-Src mutant lentivirus were grown on fibronectin-coated glass coverslips and supernatant was harvested (without centrifugation) after 24 h. The conditioned medium was transferred onto wild-type HUVECs grown on fibronectin-coated glass coverslips and incubated for 24 h before fixation and ICC staining with fibronectin and VE-cadherin.

### Drug treatments

HUVECs and HAECs were treated with 5 µM Marimastat (Sigma-Aldrich, M2699, in DMSO) or 0.01% DMSO as vehicle control in EGM-Plus medium. In 2D experiments, HUVECs were adhered for 2–4 h before Marimastat or control was administered for 24 h. Similarly, in microvessels, HUVECs and HAECs were adhered for 2–4 h before Marimastat treatment once per day for 3 days. In fibrin sprouting assays, HUVECs were coated on beads and embedded in the fibrin gel for 24 h before Marimastat treatment was administered on days 1, 3 and 5, before fixation at day 7. For VEGF-A stimulation, confluent HUVECs were starved overnight in EBM-Plus with 1% FBS prior to addition of 100 ng/ml VEGF-A165 (Thermo Fisher Scientific, PHC9391, in 0.1% BSA in PBS) in starvation medium. For EdU cell proliferation assay, cells were incubated for 2 h in 10 µM EdU labelling in EGM-Plus medium. Detection of EdU was performed according to the manufacture's protocol (Invitrogen, C10340).

### TIRF microscopy

HUVECs were grown for 4–6 h at sub-confluency on #1.5 coverslips (Hurst Scientific, 0117500) before fixing and staining as for regular ICC. The critical angle was set for each condition using a Nikon Ti2 inverted microscope stand with Andor dragonfly spinning disc Scanhead with 100×/1.49 total internal reflection fluorescence (TIRF) oil objective.

### Migration assay

HUVECs transduced with mScarlet-tagged control or c-Src mutants and were selected with puromycin and seeded into glass bottom 24-well plates (Ibidi, 82426) containing a silicon barrier (Ibidi, 80209) and grown until confluent. The silicon barrier was removed, and time-lapse imaging of the scratch area was undertaken for 16 h with 10 min interval on Nikon Deconvolution Ti-E Inverted Microscope with a 10×/0.45 air objective. Alternatively, the silicon barrier was removed, and cells were allowed to migrate for 3 h before fixation and immunofluorescence staining for fibronectin, FAs and nuclei.

### Image analysis and quantification

For FA analysis in FIJI software, maximum *Z*-projections were made, and filtered using Gaussian blur (sigma=1) before sharpening. The FA signal was thresholded using the MaxEntropy, individual cells were traced using the VE-cadherin border, and FA count, area and density were measured within each cell using particle analyser (size: 0.25 µm^2^–infinity).

Vascular area and vascular area shape were quantified in FIJI software by tracing the perimeter of the cell area in a minimum *Z*-projection of the brightfield image and subtracting the area of the bead, measuring the size and circularity respectively. The number of sprouts was measured as the number of vessels extending from the bead.

Microfluidic vessel width was measured in two axes (*Y* and *Z*) in Imaris. Five evenly spaced points across the tube were measured with a straight line and the mean was calculated per image to produce 2–3 length measurements per vessel. Linearity was measured by tracing the edge of the vessel (marked by phalloidin) and dividing this length by the length of a straight line through the vessel edge in FIJI software. To measure the number of cells per image, the Imaris spot counter was used to identify and quantify DAPI nuclei numbers. Microfluidic vessel coverage was measured by tracing any holes in the vessel wall (the inverse of cell area marked by phalloidin) and dividing this by the total cell area per image.

The TIRF critical angle was adjusted in accordance with variations in coverslip thickness. Images were used to analyse intensity plots in FIJI software, which were generated with a line of 7 µm length and 3 µm width. The FA channel was processed by background subtraction (rolling ball radius=50.0), followed by Gaussian blur (sigma=1.0).

Proliferation was quantified in sub-confluent cells by counting number of nuclei containing Ki-67 as a ratio to total number of nuclei stained with DAPI.

Fibrillar area was calculated by subtracting background (rolling ball radius=50.0) and thresholding the fibronectin channel using MaxEntropy and measuring the area.

### Statistical methods

GraphPad Prism was use for generating the graphs and to perform statistical testing. Specification on which statistical test was used for each graph can be found in the figure legends. Only significant comparisons are displayed in the graphs, *P*-values of all comparisons can be found in Table S1.

## Supplementary Material



10.1242/joces.262101_sup1 Supplementary information
