# Supplementary material for: c-Src-induced vascular malformations require localised matrix degradation at focal adhesions
Source: J Cell Sci. 2024 Jul 10;137(13):jcs262101. doi: 10.1242/jcs.262101 (PMC11267457; doi:10.1242/jcs.262101)
Supplement: Supplementary information [file joces-137-262101-s1.pdf]

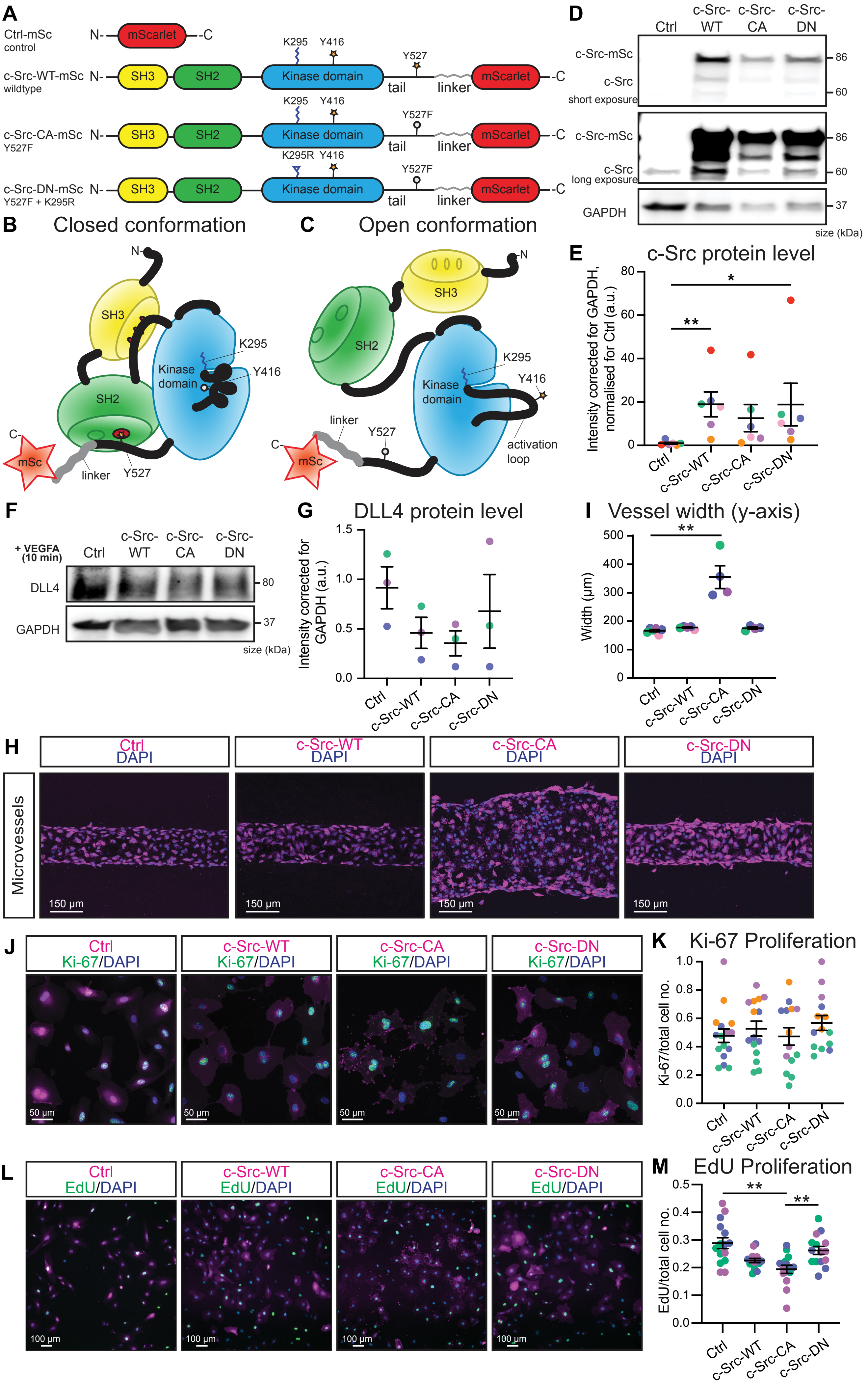
A
Ctrl-mSc
control
c-Src-WT-mSc
wildtype
c-Src-CA-mSc
Y527F
c-Src-DN-mSc
Y527F + K295R
mScarlet
SH3
SH2
Kinase domain
K295
K295R
Y416
Y527
Y527F
tail
linker
B Closed conformation
C Open conformation
activation loop
D
Ctrl
c-Src-WT
c-Src-CA
c-Src-DN
c-Src-mSc
c-Src
short exposure
long exposure
GAPDH
size (kDa)
E c-Src protein level
Intensity corrected for GAPDH, normalised for Ctrl (a.u.)
F
+ VEGFA (10 min)
DLL4
G DLL4 protein level
Intensity corrected for GAPDH (a.u.)
I Vessel width (y-axis)
Width (µm)
H
Microvessels
Ctrl DAPI
c-Src-WT DAPI
c-Src-CA DAPI
c-Src-DN DAPI
150 µm
J
Ki-67/DAPI
50 µm
K Ki-67 Proliferation
Ki-67/total cell no.
L
EdU/DAPI
100 µm
M EdU Proliferation
EdU/total cell no.

Fig. S1. Expression of c-Src mutants in endothelial cells.
(A) Schematic of constructs: Control (Ctrl-mSc) containing an empty vector with mScarlet, wildtype c-Src (c-Src-WT-mSc) containing c-Src with C-terminal mScarlet tag, constitutive active c-Src (c-Src-CA-mSc) containing c-Src with a Y527F point mutation, dominant negative c-Src (c-Src-DN-mSc) containing c-Src with a double point mutation at Y527F and K295R sites. **(B)** Schematic of c-Src in closed conformation due to phosphorylated autoinhibitory Tyr527 site binding to the SH2 domain. **(C)** Schematic of c-Src in open conformation due to dephosphorylation of Tyr527, exposing the SH2 and SH3 domains for binding of target proteins, and the activation loop including Y416 for phosphorylation and activation of c-Src. **(D)** Representative Western blot of HUVEC transduced with mScarlet-tagged control, c-Src or c-Src mutants, showing endogenous c-Src at 60 kDa and overexpressing c-Src-mScarlet at 86 kDa. GAPDH was used as loading control. **(E)** Quantification of c-Src protein expression levels. n = 6 independent experiments. **(F)** Representative Western blot of HUVEC transduced with mScarlet-tagged control, or c-Src mutants stimulated with 100 ng/ml VEGF-A for 10 minutes, showing DLL4. GAPDH was used as loading control. **(G)** Quantification of DLL4 protein expression levels. n = 3 independent experiments. **(H)** Representative images of HAECs transduced with mScarlet-tagged c-Src mutants (magenta) seeded in PDMS microfluidic vessels containing 2.5 mg/mL collagen matrix for 3 days before fixing. Immunofluorescent staining was performed for nuclei (DAPI; blue). **(I)** Quantification of vessel width. n = 3-4 independent experiments, 1-2 vessels per replicate with each data point representing an average of 3-5 measurements per vessel. **(J)** Representative images of sub-confluent mScarlet-tagged c-Src mutant HUVECs (magenta) grown overnight before fixing. Immunofluorescent staining was performed for proliferating cells (Ki-67; green) and nuclei (DAPI; blue). **(K)** Quantification of proliferation in sub-confluent monolayers measured by ratio of Ki-67-positive cells to total number of cells. n = 4 independent experiments. **(L)** Representative images of sub-confluent mScarlet-tagged c-Src mutant HUVECs (magenta) grown overnight before 2h Edu incorporation. Click chemistry was performed for proliferating cells (EdU; green) and immunofluorescent staining for nuclei (DAPI; blue). **(M)** Quantification of proliferation in sub-confluent monolayers measured by ratio of EdU-positive cells to total number of cells. n = 3 independent experiments. All data are represented as mean ± SEM with individual data point indicated and colours represent independent experiments. Statistical significance was determined using Kruskal-Wallis test with Dunn's multiple comparisons (E, G, I, K, M). * $p<0.05$, ** $p<0.01$

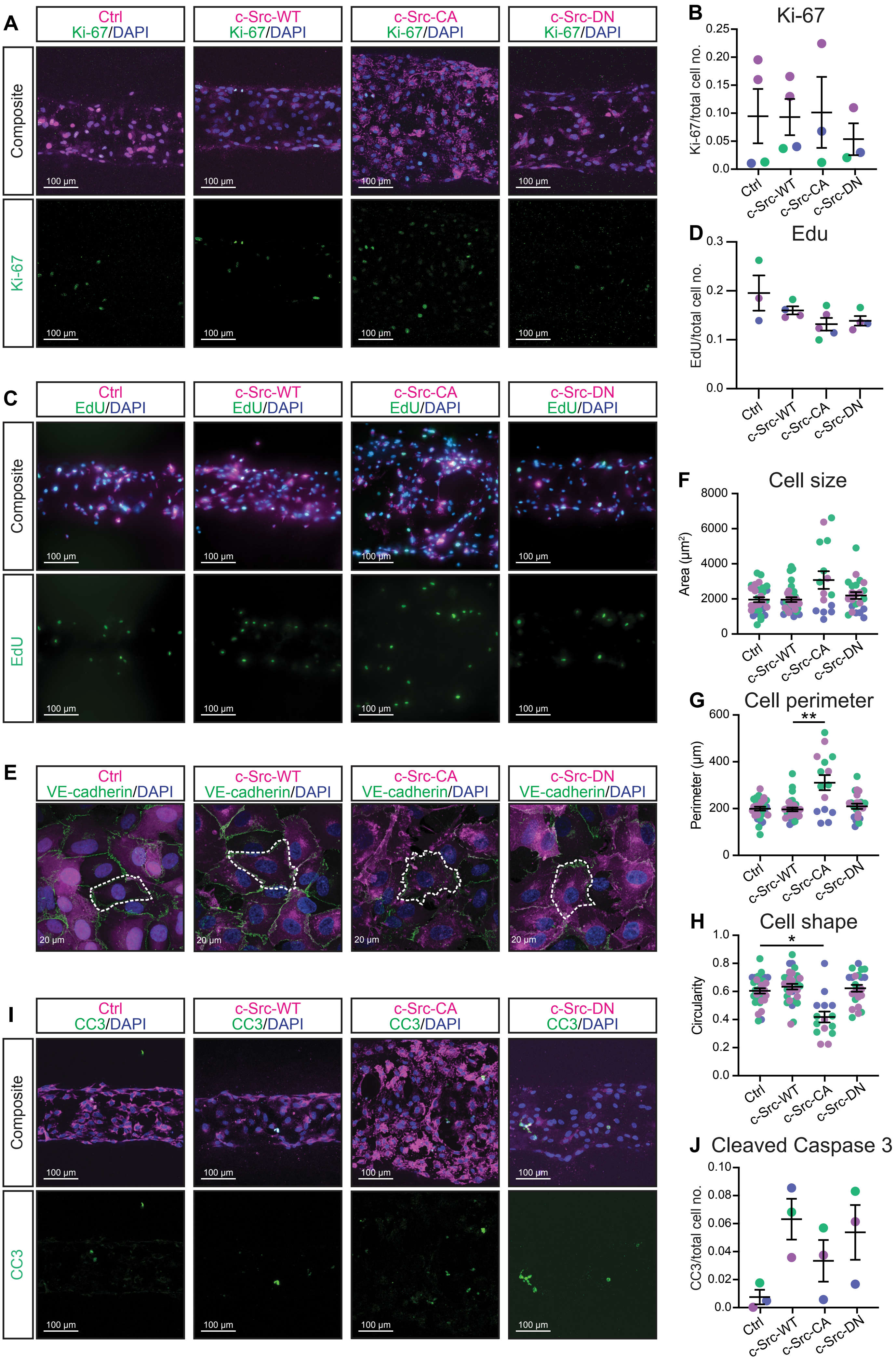
A
Ctrl
Ki-67/DAPI
c-Src-WT
Ki-67/DAPI
c-Src-CA
Ki-67/DAPI
c-Src-DN
Ki-67/DAPI
Composite
Ki-67
100 µm
B
Ki-67
Ki-67/total cell no.
Ctrl
c-Src-WT
c-Src-CA
c-Src-DN
C
EdU/DAPI
EdU
D
Edu
EdU/total cell no.
E
VE-cadherin/DAPI
20 µm
F
Cell size
Area (µm²)
G
Cell perimeter
Perimeter (µm)
**
H
Cell shape
Circularity
*
I
CC3/DAPI
CC3
J
Cleaved Caspase 3
CC3/total cell no.

Fig. S2. Effects of constitutively active c-Src on proliferation and apoptosis in 3D culture. **(A)** Representative images of mScarlet-tagged c-Src mutant HUVECs (magenta) seeded in PDMS microfluidic vessels containing 2.5 mg/mL collagen matrix for 3 days before fixing. Immunofluorescent staining was performed for proliferating cells (Ki-67; green) and nuclei (DAPI; blue). Bottom panel shows individual Ki-67 channel in green. **(B)** Quantification of proliferation in 3D microfluidic vessels measured by ratio of Ki-67-positive cells to total number of cells. n = 3 independent experiments with 1-2 vessels per replicate. (C) Representative images of mScarlet-tagged c-Src mutant HUVECs (magenta) seeded in PDMS microfluid vessels containing 2.5 mglmL collagen matrix for 3 days before 2h Edu incorporation and fixing. Click chemistry was performed for proliferating cells (EdU; green) and immunofluorescent staining for nuclei (DAPI; blue). Bottom panel shows individual EdU channel in green. **(D)** Quantification of proliferation in 3D microfluidic vessels measured by ratio of EdU-positive cells to total number of cells. n = 3 independent experiments. **(E)** Representative images of mScarlet-tagged c-Src mutant HUVECs (magenta) grown to confluent monolayer before fixing and immunofluorescent staining was performed for VE-cadherin (green) and nuclei (DAPI; blue). Cell borders outlined in white. Quantification of confluent monolayers for cell size **(F)**, cell perimeter **(G)**, and cell shape **(H)**. n = 3 independent experiments. **(I)** Representative images of mScarlet-tagged c-Src mutant HUVECs (magenta) seeded in PDMS microfluidic vessels containing 2.5 mg/mL collagen matrix for 3 days before fixing. Immunofluorescent staining was performed for apoptotic cells (Cleaved Caspase 3 – CC3; green) and nuclei (DAPI; blue). Bottom panel shows individual CC3 channel in green. **(J)** Quantification of apoptosis in 3D microfluidic vessels measured by ratio of Cleaved Caspase 3-positive cells to total number of cells. n = 3 independent experiments with 1 vessel per replicate. All data are represented as mean ± SEM with individual data point indicated and colours represent independent experiments. Statistical significance was determined using Kruskal-Wallis test with Dunn's multiple comparisons (B, D-F, H). * $p<0.05$, ** $p<0.01$.

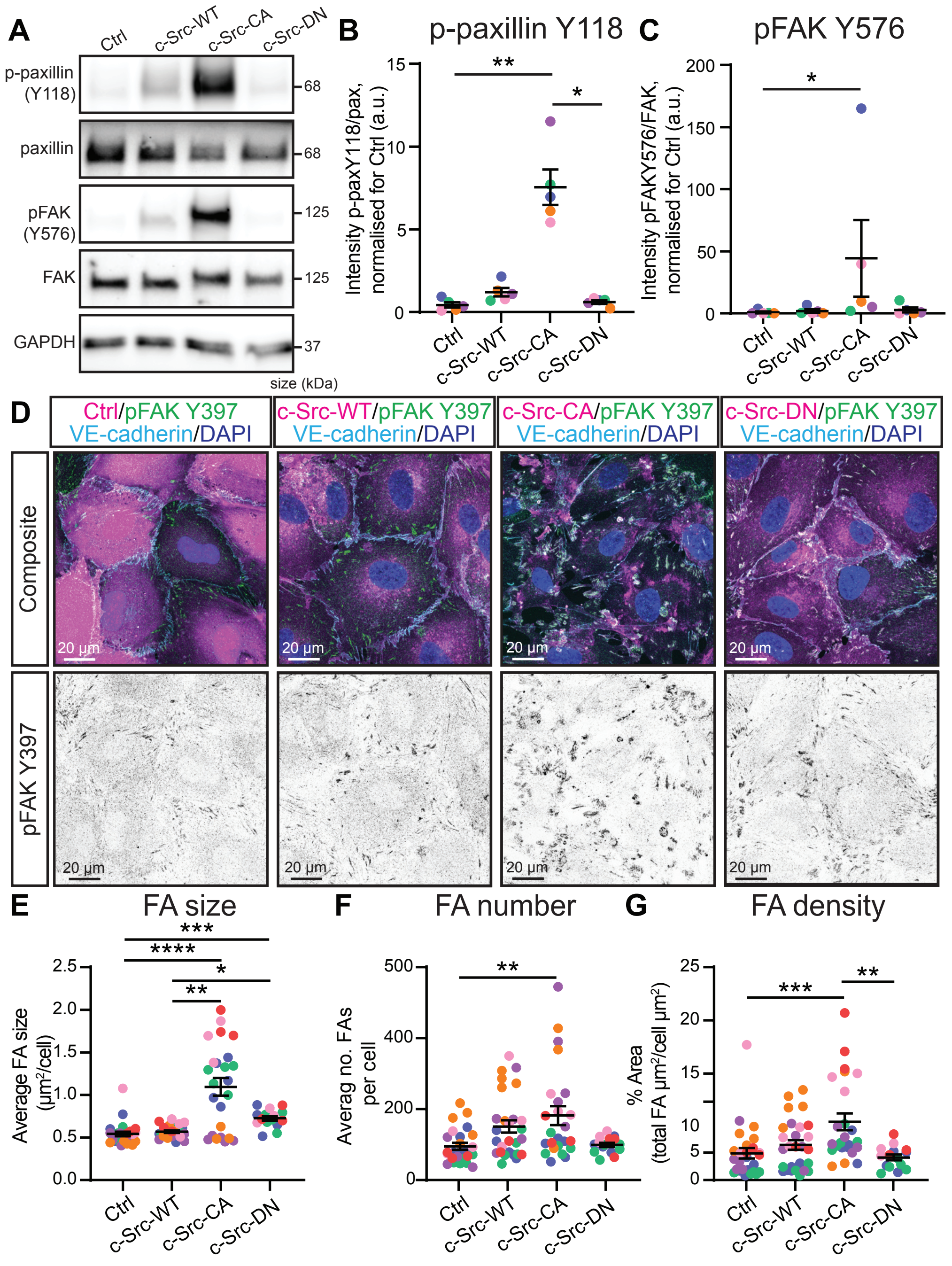

Fig. S3. Effects of constitutively active c-Src on paxillin and FAK.

(A) Representative Western blot for phosphorylation of paxillin (Y118) and FAK(Y576) in HUVECs transduced with c-Src mutant cells. **(B)** Quantification of paxillin phosphorylation on Y118 in relation to total paxillin corrected for loading control (GAPDH) and normalised to control cells. n = 5 independent experiments. **(C)** Quantification of FAK phosphorylation on Y576 in relation to total FAK corrected for loading control (GAPDH) and normalised to control cells. n = 5 independent experiments. **(D)** Representative images of confluent monolayers of mScarlet-tagged c-Src mutant HUVECs (magenta) fixed and immunofluorescent staining was performed for phospho-FAK (Y397; green, shown as individual black channel in bottom panel), VE-cadherin (cyan), and nuclei (DAPI; blue). Quantification of average focal adhesion size **(E)**, number **(F)**, and density **(G)** per cell. n = 4-6 independent experiments, 5 images per replicate with each data point representing an average of 3-5 cells per image. All data are represented as mean ± SEM with individual data point indicated and colours represent independent experiments. Statistical significance was determined using Kruskal-Wallis test with Dunn's multiple comparisons (B, C, E-G). The mean value of each individual replicate and corresponding SEM, instead of all data points separately, was used for statistical analysis (E-G). * $p<0.05$, ** $p<0.01$, *** $p<0.0005$, **** $p<0.0001$.

Journal of Cell Science • Supplementary information

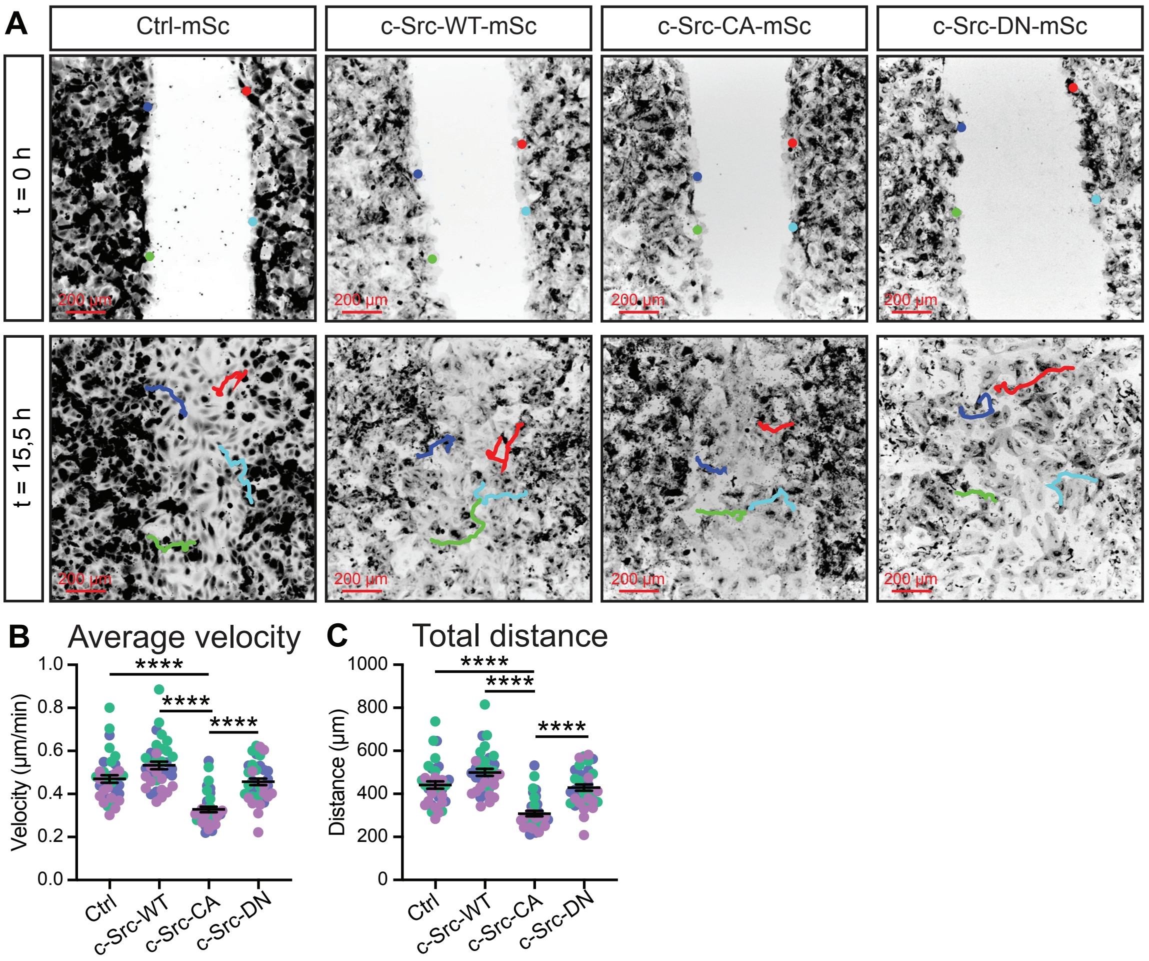

Fig. S4. Constitutively active c-Src reduces cell migration in 2D.
(A) Representative still images of mScarlet-tagged c-Src mutant HUVEC migrating in a scratch assay over the course of 15.5 h. Individual tracked cell paths are highlighted. n = 3 independent experiments. Quantification of average velocity **(B)**, and total distance travelled within 15.5 h **(C)**. All data are represented as mean ± SEM with individual data point indicated and colours represent independent experiments. Statistical significance was determined using Kruskal-Wallis test with Dunn's multiple comparisons (B, C). **** $p<0.0001$.

Journal of Cell Science • Supplementary information

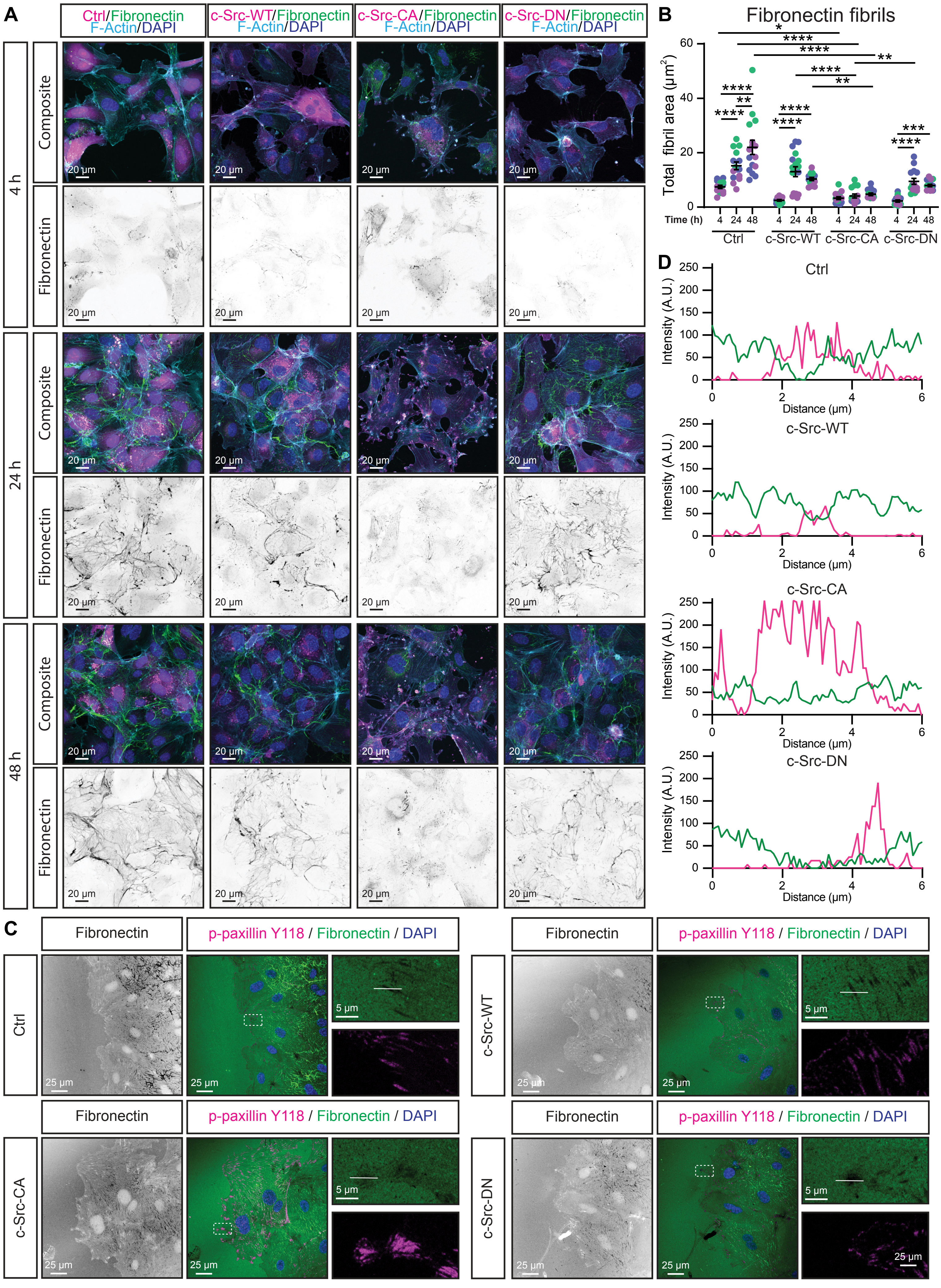

Journal of Cell Science • Supplementary information

Fig. S5. Fibronectin fibril deposition is inhibited by constitutively active c-Src.
(A) Representative images of mScarlet-tagged c-Src mutant HUVECs (magenta) grown on uncoated glass for 4, 24 or 48 h to allow fibronectin deposition and remodelling before fixing. Immunofluorescent staining was performed for fibronectin (green, shown as individual black channel in bottom panels), F-Actin (Phalloidin; cyan), and nuclei (DAPI; blue). **(B)** Quantification of total fibronectin fibril area per image. n = 3 independent experiments. **(C)** Representative confocal images of mScarlet-tagged c-Src mutant HUVECs grown in Ibidi culture inserts on fibronectin. Cells were allowed to migrate after insert removal for 3 h before fixing. Immunofluorescent staining was performed for fibronectin (green), focal adhesions (p-paxillin Y118; magenta) and nuclei (DAPI; blue). Single channel fibronectin panel shows degradation spots lack IF signal (white). White dashed box indicates region of higher magnification, single channel images show next to the merge, white lines indicate area used to generate intensity plots. **(D)** Intensity plots of areas of interest indicated by white lines in (C) to demonstrate a decrease in fibronectin (green) intensity at sites of increased focal adhesion signal (magenta). All lines are 6 μm and traverse through a focal adhesion. All data are represented as mean ± SEM with individual data point indicated and colours represent independent experiments. Statistical significance was determined using two-way ANOVA with Turkey's multiple comparisons test (B). * $p<0.05$, ** $p<0.01$, *** $p<0.0005$, **** $p<0.0001$.

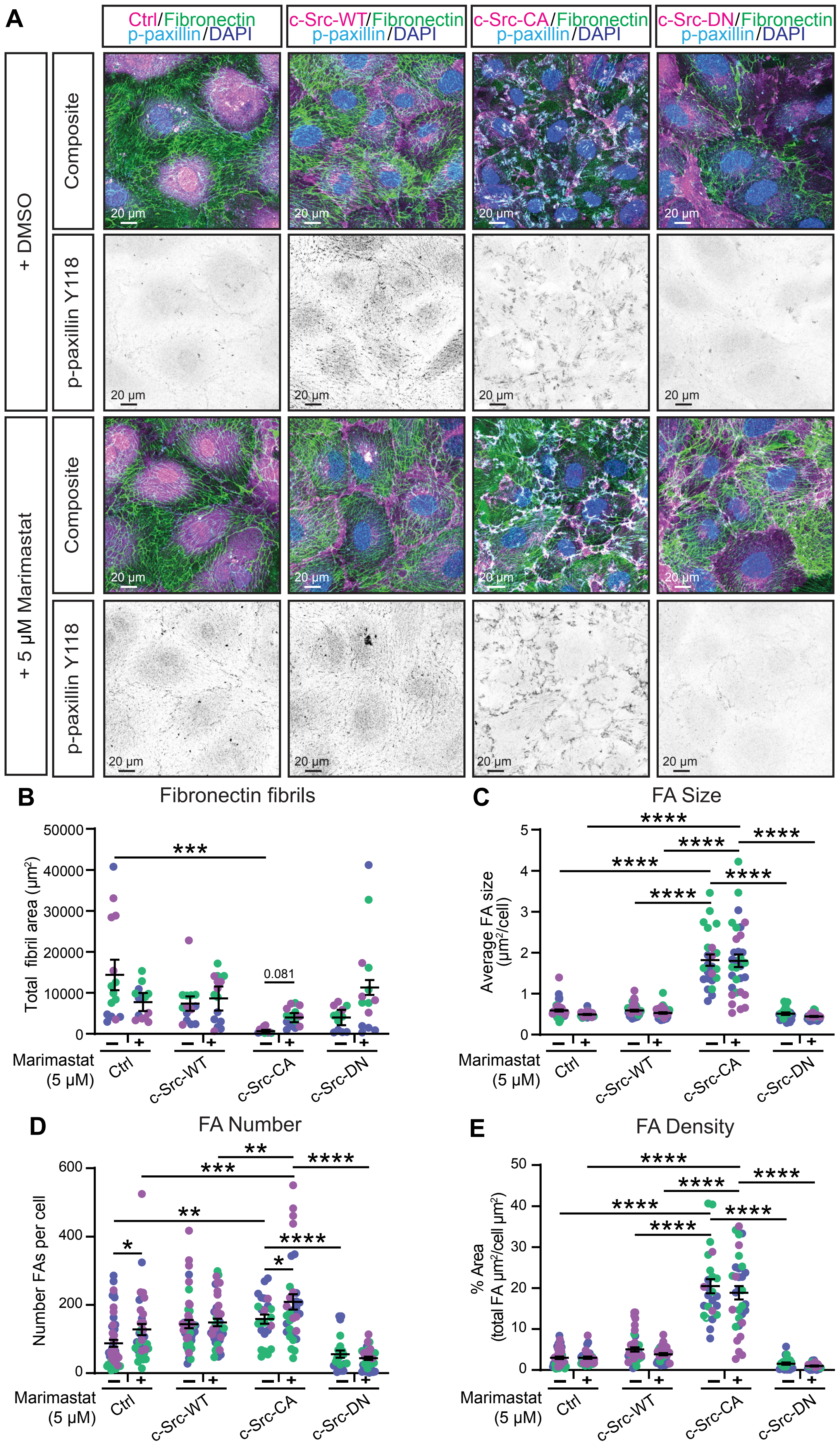

A
Ctrl/Fibronectin p-paxillin/DAPI
c-Src-WT/Fibronectin p-paxillin/DAPI
c-Src-CA/Fibronectin p-paxillin/DAPI
c-Src-DN/Fibronectin p-paxillin/DAPI
+ DMSO
+ 5 µM Marimastat
Composite
p-paxillin Y118
20 µm
B
Fibronectin fibrils
Total fibril area (µm²)
0.081
***
Marimastat (5 µM)
Ctrl
c-Src-WT
c-Src-CA
c-Src-DN
C
FA Size
Average FA size (µm²/cell)
****
D
FA Number
Number FAs per cell
**
***
*
****
E
FA Density
% Area (total FA µm²/cell µm²)
****

Fig. S6. MMP inhibition rescues fibronectin fibril depositions but focal adhesion upregulation is not affected.

(A) Representative images of mScarlet-tagged c-Src mutant HUVECs (magenta) grown on fibronectin-coated glass and treated with DMSO vehicle control or 5 µM Marimastat at 4 h post-seeding. Cells were grown in presence of DMSO or Marimastat for 24 h before fixing and immunostaining for fibronectin (green), phospho-paxillin Y118 (cyan, shown as individual black channel in bottom panels), and nuclei (DAPI; blue). **(B)** Quantification of total fibronectin fibril area per image. Quantification of average focal adhesion size **(C)**, number **(D)**, and density **(E)** per cell. n = 3 independent experiments. All data are represented as mean ± SEM with individual data point indicated and colours represent independent experiments. Statistical significance was determined using two-way ANOVA with Turkey's multiple comparisons test (B-E). The mean value of each individual replicate and corresponding SEM, instead of all data points separately, was used for statistical analysis (B). * p<0.05, ** p<0.01, *** p<0.0005, **** p<0.0001.

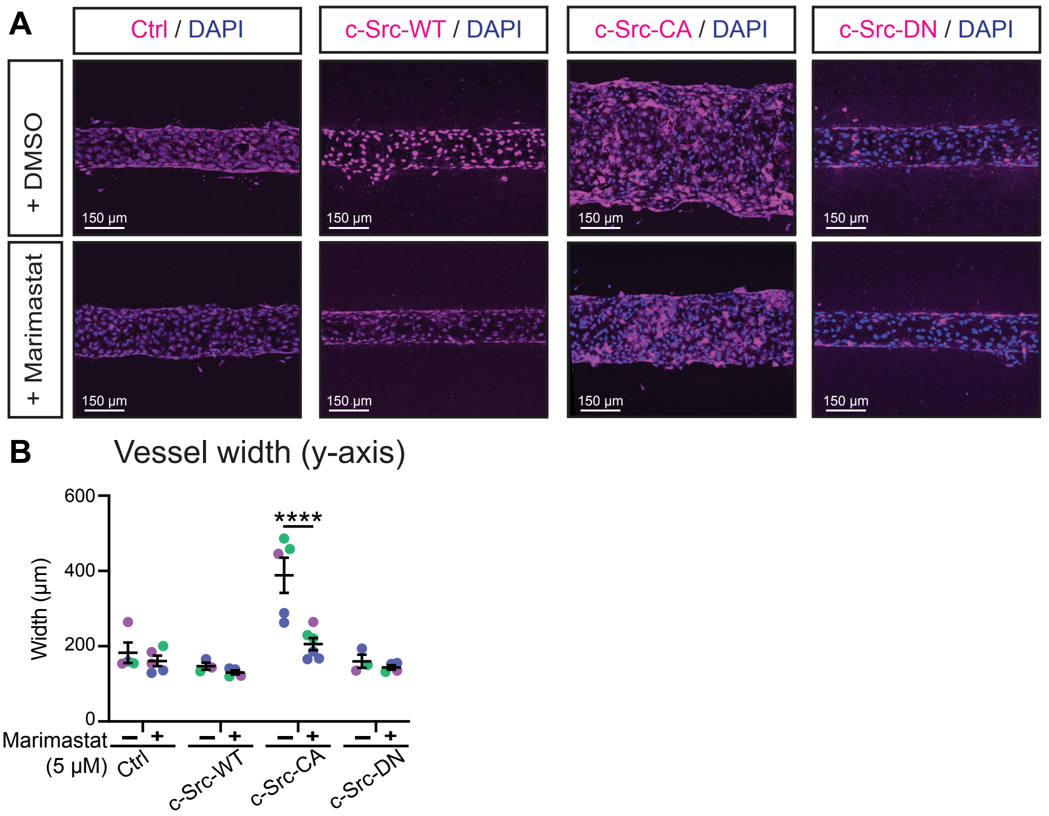

Fig. S7. Broad spectrum MMP inhibitor Marimastat rescues vascular ballooning in constitutively active c-Src in 3D microvessels with HAEC.

(A) Representative images of HAECs transduced with mScarlet-tagged c-Src mutants (magenta) seeded in PDMS microfluidic vessels containing 2.5 mg/mL collagen matrix for 3 days and treated daily with DMSO vehicle control or 5 µM Marimastat before fixing. Immunofluorescent staining was performed for nuclei (DAPI; blue). (B) Quantification of vessel width. n = 3 independent experiments, 3 images per replicate with each data point representing an average of 5 measurements per image. All data are represented as mean SEM with individual data point indicated and colours represent independent experiments. Statistical significance was determined using two-way ANOVA with Sidak's multiple comparisons test. The mean value of each individual replicate and corresponding SEM, instead of all data points separately, was used for statistical analysis (B). **** p<0.0001.

Journal of Cell Science • Supplementary information

Fig 3A

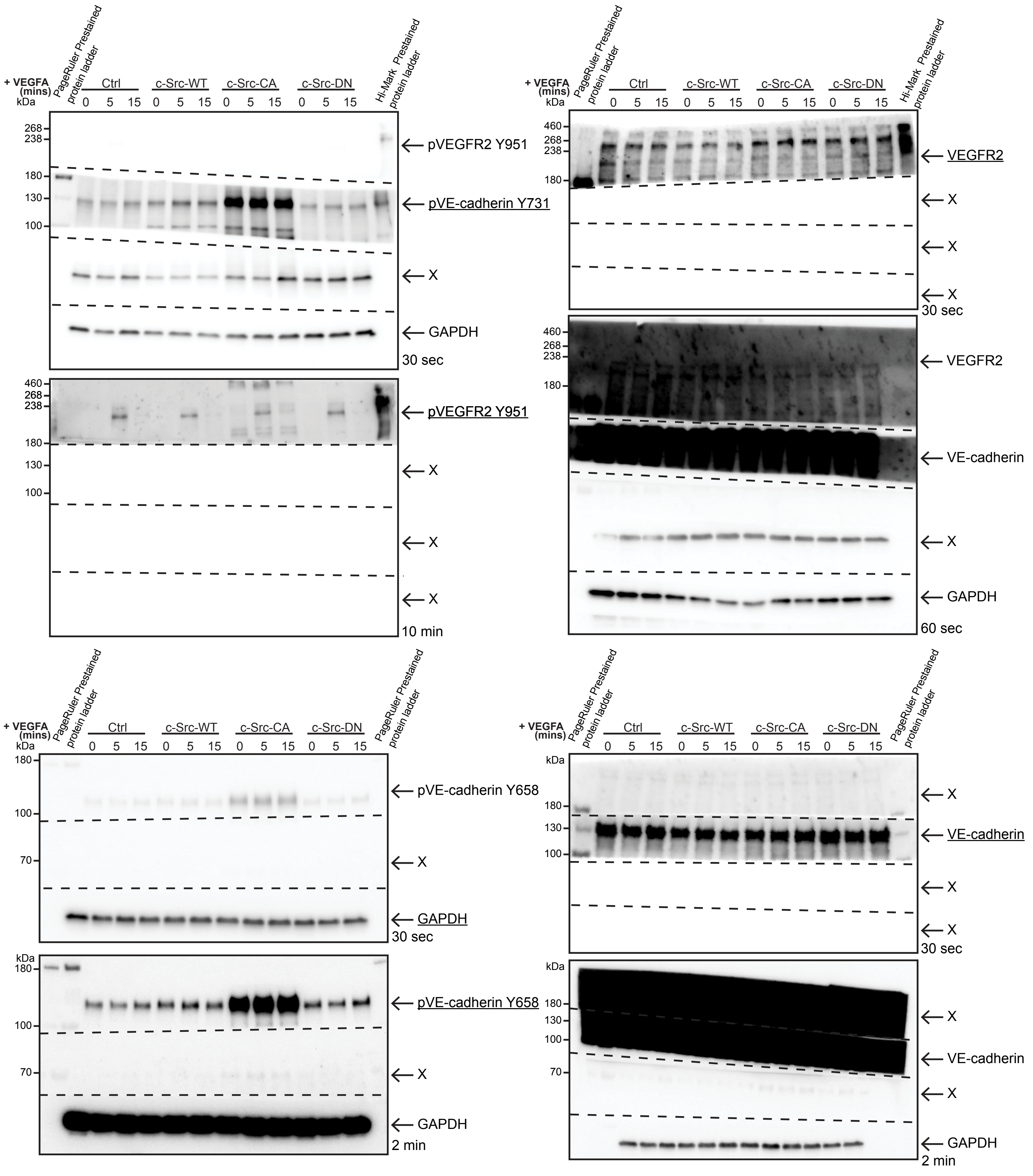

Journal of Cell Science • Supplementary information

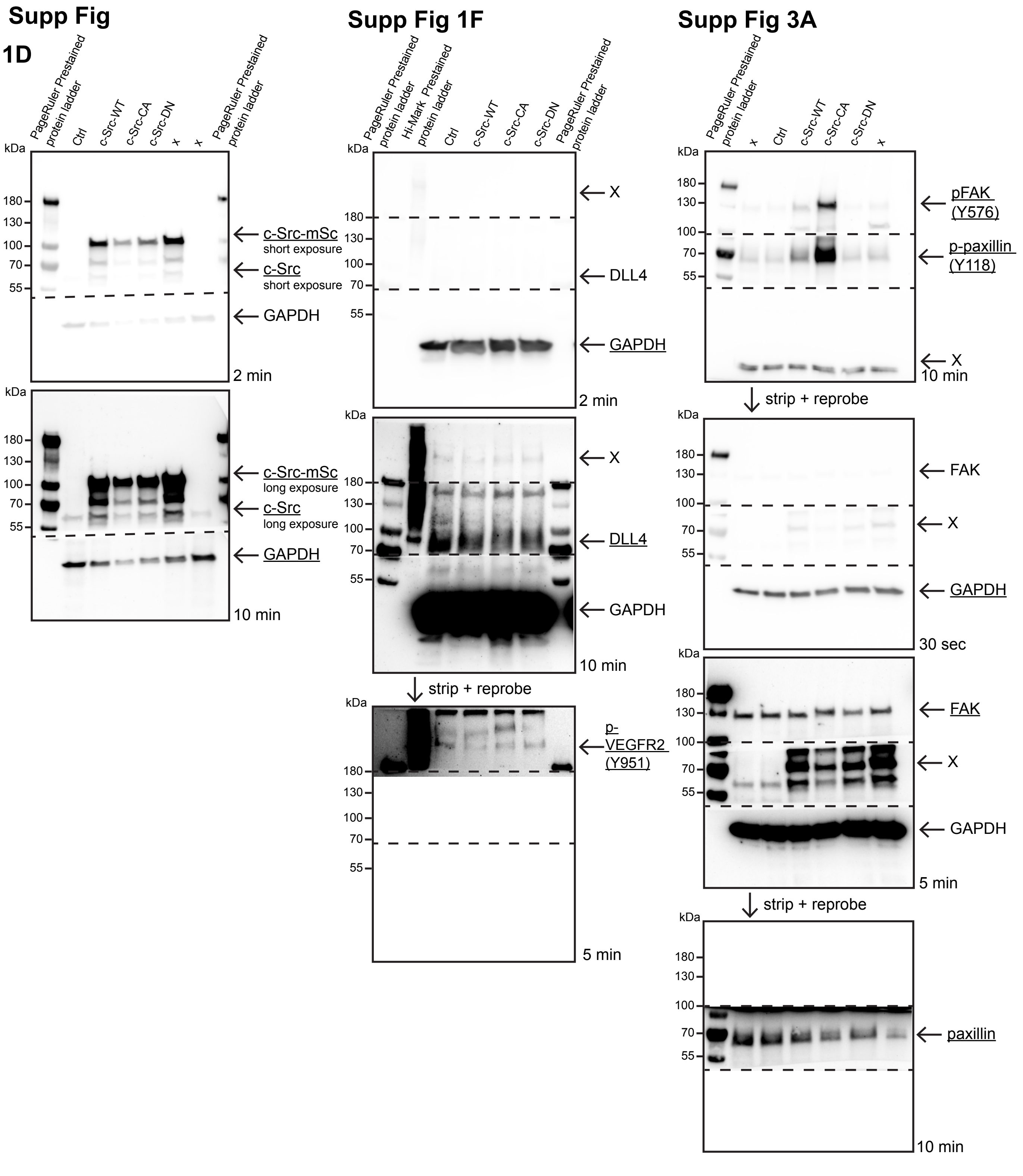

Fig. S8. Uncropped scans of Western blots.

Full, uncropped scans of Western blots shown in Fig 3A, Fig. S1D, Fig. S1F, Fig. S3A. Used lanes are labelled as in the corresponding figure, with other lanes labelled as X. Dashed line indicates where membrane was cut. Underlined proteins indicate which blot was used in the corresponding figure and time indicates exposure time.

Journal of Cell Science • Supplementary information

Table S1. Summary of all statistical tests and p values.

List of all the different statistical comparisons that were made per graph and their respective p values.

Fig. 1B. Vascular area

**Kruskal-Wallis test**	
P value	<0.0001
Exact or approximate P value?	Approximate
P value summary	****
Do the medians vary signif. (P < 0.05)?	Yes
Number of groups	4
Kruskal-Wallis statistic	47.39

**Dunn's multiple comparisons test**	**Summary**	**Adjusted P Value**
Ctrl vs. c-Src-WT	ns	>0.9999
Ctrl vs. c-Src-CA	****	<0.0001
Ctrl vs. c-Src-DN	ns	0.4813
c-Src-WT vs. c-Src-CA	**	0.0016
c-Src-WT vs. c-Src-DN	*	0.0283
c-Src-CA vs. c-Src-DN	****	<0.0001

Fig. 1C. Sprout number

**Kruskal-Wallis test**	
P value	<0.0001
Exact or approximate P value?	Approximate
P value summary	****
Do the medians vary signif. (P < 0.05)?	Yes
Number of groups	4
Kruskal-Wallis statistic	60.87

**Dunn's multiple comparisons test**	**Summary**	**Adjusted P Value**
Ctrl vs. c-Src-WT	ns	0.0722
Ctrl vs. c-Src-CA	****	<0.0001
Ctrl vs. c-Src-DN	ns	0.1788
c-Src-WT vs. c-Src-CA	****	<0.0001
c-Src-WT vs. c-Src-DN	****	<0.0001
c-Src-CA vs. c-Src-DN	**	0.0058

Fig. 1D. Vascular area shape

**Kruskal-Wallis test**	
P value	<0.0001
Exact or approximate P value?	Approximate
P value summary	****
Do the medians vary signif. (P < 0.05)?	Yes
Number of groups	4
Kruskal-Wallis statistic	46.78

**Dunn's multiple comparisons test**	**Summary**	**Adjusted P Value**
Ctrl vs. c-Src-WT	ns	>0.9999
Ctrl vs. c-Src-CA	****	<0.0001
Ctrl vs. c-Src-DN	ns	0.1000
c-Src-WT vs. c-Src-CA	****	<0.0001
c-Src-WT vs. c-Src-DN	**	0.0019
c-Src-CA vs. c-Src-DN	**	0.0094

Fig. 1E. Cell number per bead

Kruskal-Wallis test	
P value	<0.0001
Exact or approximate P value?	Approximate
P value summary	****
Do the medians vary signif. (P < 0.05)?	Yes
Number of groups	4
Kruskal-Wallis statistic	33.13

Dunn's multiple comparisons test	Summary	Adjusted P Value
Ctrl vs. c-Src-WT	ns	>0.9999
Ctrl vs. c-Src-CA	****	<0.0001
Ctrl vs. c-Src-DN	ns	>0.9999
c-Src-WT vs. c-Src-CA	****	<0.0001
c-Src-WT vs. c-Src-DN	ns	>0.9999
c-Src-CA vs. c-Src-DN	**	0.0012

Fig. 1G. Vessel Width (Y)

Kruskal-Wallis test	
P value	<0.0001
Exact or approximate P value?	Approximate
P value summary	****
Do the medians vary signif. (P < 0.05)?	Yes
Number of groups	4
Kruskal-Wallis statistic	28.06

Dunn's multiple comparisons test	Summary	Adjusted P Value
Ctrl vs. c-Src-WT	ns	>0.9999
Ctrl vs. c-Src-CA	**	0.0041
Ctrl vs. c-Src-DN	ns	0.1787
c-Src-WT vs. c-Src-CA	ns	0.0680
c-Src-WT vs. c-Src-DN	*	0.0144
c-Src-CA vs. c-Src-DN	****	<0.0001

Fig. 1H. Cell number per vessel

Kruskal-Wallis test	
P value	0.4249
Exact or approximate P value?	Exact
P value summary	ns
Do the medians vary signif. (P < 0.05)?	No
Number of groups	4
Kruskal-Wallis statistic	2.988

Dunn's multiple comparisons test	Summary	Adjusted P Value
Ctrl vs. c-Src-WT	ns	>0.9999
Ctrl vs. c-Src-CA	ns	>0.9999
Ctrl vs. c-Src-DN	ns	>0.9999
c-Src-WT vs. c-Src-CA	ns	>0.9999
c-Src-WT vs. c-Src-DN	ns	0.7457
c-Src-CA vs. c-Src-DN	ns	>0.9999

Fig. 1I. Vessel edge irregularity

Kruskal-Wallis test	
P value	0.0008
Exact or approximate P value?	Approximate

Journal of Cell Science • Supplementary information

P value summary	***
Do the medians vary signif. (P < 0.05)?	Yes
Number of groups	4
Kruskal-Wallis statistic	16.80

Dunn's multiple comparisons test	Summary	Adjusted P Value
Ctrl vs. c-Src-WT	ns	>0.9999
Ctrl vs. c-Src-CA	*	0.0208
Ctrl vs. c-Src-DN	ns	>0.9999
c-Src-WT vs. c-Src-CA	***	0.0004
c-Src-WT vs. c-Src-DN	ns	0.4149
c-Src-CA vs. c-Src-DN	ns	0.3191

Fig. 1J. Cell size

Kruskal-Wallis test	
P value	0.4325
Exact or approximate P value?	Approximate
P value summary	ns
Do the medians vary signif. (P < 0.05)?	No
Number of groups	4
Kruskal-Wallis statistic	2.746

Dunn's multiple comparisons test	Summary	Adjusted P Value
Ctrl vs. c-Src-WT	ns	>0.9999
Ctrl vs. c-Src-CA	ns	>0.9999
Ctrl vs. c-Src-DN	ns	>0.9999
c-Src-WT vs. c-Src-CA	ns	>0.9999
c-Src-WT vs. c-Src-DN	ns	0.9496
c-Src-CA vs. c-Src-DN	ns	>0.9999

Fig. 1K. Cell shape

Kruskal-Wallis test	
P value	0.0028
Exact or approximate P value?	Approximate
P value summary	**
Do the medians vary signif. (P < 0.05)?	Yes
Number of groups	4
Kruskal-Wallis statistic	14.11

Dunn's multiple comparisons test	Summary	Adjusted P Value
Ctrl vs. c-Src-WT	ns	>0.9999
Ctrl vs. c-Src-CA	ns	0.7442
Ctrl vs. c-Src-DN	ns	0.0689
c-Src-WT vs. c-Src-CA	ns	0.6113
c-Src-WT vs. c-Src-DN	*	0.0342
c-Src-CA vs. c-Src-DN	**	0.0011

Fig. 1L. Vessel coverage

Kruskal-Wallis test	
P value	0.0008
Exact or approximate P value?	Approximate
P value summary	***
Do the medians vary signif. (P < 0.05)?	Yes
Number of groups	4
Kruskal-Wallis statistic	16.61

Journal of Cell Science • Supplementary information

**Dunn's multiple comparisons test**	**Summary**	**Adjusted P Value**
Ctrl vs. c-Src-WT	ns	>0.9999
Ctrl vs. c-Src-CA	**	0.0011
Ctrl vs. c-Src-DN	ns	>0.9999
c-Src-WT vs. c-Src-CA	ns	0.1025
c-Src-WT vs. c-Src-DN	ns	>0.9999
c-Src-CA vs. c-Src-DN	**	0.0051

Fig. 2B. 2D FA size

Kruskal-Wallis test	
P value	0.0020
Exact or approximate P value?	Exact
P value summary	**
Do the medians vary signif. (P < 0.05)?	Yes
Number of groups	4
Kruskal-Wallis statistic	9.359

**Dunn's multiple comparisons test**	**Summary**	**Adjusted P Value**
Ctrl vs. c-Src-WT	ns	0.6775
Ctrl vs. c-Src-CA	*	0.0134
Ctrl vs. c-Src-DN	ns	0.8462
c-Src-WT vs. c-Src-CA	ns	0.8462
c-Src-WT vs. c-Src-DN	ns	>0.9999
c-Src-CA vs. c-Src-DN	ns	0.6775

Fig. 2C. 2D FA number

**Kruskal-Wallis test**	
P value	0.0382
Exact or approximate P value?	Exact
P value summary	*
Do the medians vary signif. (P < 0.05)?	Yes
Number of groups	4
Kruskal-Wallis statistic	7.205

**Dunn's multiple comparisons test**	**Summary**	**Adjusted P Value**
Ctrl vs. c-Src-WT	ns	>0.9999
Ctrl vs. c-Src-CA	ns	0.0552
Ctrl vs. c-Src-DN	ns	>0.9999
c-Src-WT vs. c-Src-CA	ns	0.5366
c-Src-WT vs. c-Src-DN	ns	>0.9999
c-Src-CA vs. c-Src-DN	ns	0.4202

Fig. 2D. 2D FA density

**Kruskal-Wallis test**	
P value	0.0028
Exact or approximate P value?	Exact
P value summary	**
Do the medians vary signif. (P < 0.05)?	Yes
Number of groups	4
Kruskal-Wallis statistic	9.154

**Dunn's multiple comparisons test**	**Summary**	**Adjusted P Value**
Ctrl vs. c-Src-WT	ns	0.5366
Ctrl vs. c-Src-CA	*	0.0194

Journal of Cell Science • Supplementary information

Ctrl vs. c-Src-DN	ns	>0.9999
c-Src-WT vs. c-Src-CA	ns	>0.9999
c-Src-WT vs. c-Src-DN	ns	>0.9999
c-Src-CA vs. c-Src-DN	ns	0.3255

Fig. 2F. 3D FA size

**Kruskal-Wallis test**	
P value	0.0008
Exact or approximate P value?	Exact
P value summary	***
Do the medians vary signif. (P < 0.05)?	Yes
Number of groups	4
Kruskal-Wallis statistic	9.513

**Dunn's multiple comparisons test**	**Summary**	**Adjusted P Value**
Ctrl vs. c-Src-WT	ns	0.4202
Ctrl vs. c-Src-CA	*	0.0194
Ctrl vs. c-Src-DN	ns	>0.9999
c-Src-WT vs. c-Src-CA	ns	>0.9999
c-Src-WT vs. c-Src-DN	ns	>0.9999
c-Src-CA vs. c-Src-DN	ns	0.2492

Fig. 2G. 3D FA number

**Kruskal-Wallis test**	
P value	0.0681
Exact or approximate P value?	Exact
P value summary	ns
Do the medians vary signif. (P < 0.05)?	No
Number of groups	4
Kruskal-Wallis statistic	6.590

**Dunn's multiple comparisons test**	**Summary**	**Adjusted P Value**
Ctrl vs. c-Src-WT	ns	>0.9999
Ctrl vs. c-Src-CA	ns	0.1412
Ctrl vs. c-Src-DN	ns	>0.9999
c-Src-WT vs. c-Src-CA	ns	>0.9999
c-Src-WT vs. c-Src-DN	ns	>0.9999
c-Src-CA vs. c-Src-DN	ns	0.1887

Fig. 2H. 3D FA density

**Kruskal-Wallis test**	
P value	0.0284
Exact or approximate P value?	Exact
P value summary	*
Do the medians vary signif. (P < 0.05)?	Yes
Number of groups	4
Kruskal-Wallis statistic	7.513

**Dunn's multiple comparisons test**	**Summary**	**Adjusted P Value**
Ctrl vs. c-Src-WT	ns	>0.9999
Ctrl vs. c-Src-CA	ns	0.0552
Ctrl vs. c-Src-DN	ns	>0.9999
c-Src-WT vs. c-Src-CA	ns	0.8462
c-Src-WT vs. c-Src-DN	ns	>0.9999
c-Src-CA vs. c-Src-DN	ns	0.2492

Journal of Cell Science • Supplementary information

Fig. 3B. pVEGFR2 Y951

Two-way ANOVA	Ordinary		
Alpha	0.05		
Source of Variation	% of total variation	P value	P value summary
Interaction	26.54	0.0145	*
Row Factor	27.96	0.0005	***
Column Factor	14.20	0.0273	*

Tukey's multiple comparisons test	Summary	Adjusted P Value
0		
Ctrl vs. c-Src-WT	ns	>0.9999
Ctrl vs. c-Src-CA	ns	>0.9999
Ctrl vs. c-Src-DN	ns	>0.9999
c-Src-WT vs. c-Src-CA	ns	>0.9999
c-Src-WT vs. c-Src-DN	ns	>0.9999
c-Src-CA vs. c-Src-DN	ns	>0.9999
5		
Ctrl vs. c-Src-WT	***	0.0004
Ctrl vs. c-Src-CA	*	0.0200
Ctrl vs. c-Src-DN	***	0.0003
c-Src-WT vs. c-Src-CA	ns	0.3688
c-Src-WT vs. c-Src-DN	ns	>0.9999
c-Src-CA vs. c-Src-DN	ns	0.3640
15		
Ctrl vs. c-Src-WT	ns	0.9993
Ctrl vs. c-Src-CA	ns	0.9984
Ctrl vs. c-Src-DN	ns	0.9997
c-Src-WT vs. c-Src-CA	ns	0.9913
c-Src-WT vs. c-Src-DN	ns	>0.9999
c-Src-CA vs. c-Src-DN	ns	0.9938
Ctrl		
0 vs. 5	****	<0.0001
0 vs. 15	ns	0.9805
5 vs. 15	****	<0.0001
c-Src-WT		
0 vs. 5	ns	0.8874
0 vs. 15	ns	0.9984
5 vs. 15	ns	0.9109
c-Src-CA		
0 vs. 5	ns	0.1122
0 vs. 15	ns	0.9461
5 vs. 15	ns	0.1985
c-Src-DN		
0 vs. 5	ns	0.8891
0 vs. 15	ns	0.9954
5 vs. 15	ns	0.9268

Fig. 3C. pVE-cadherin Y658

Two-way ANOVA	Ordinary		
Alpha	0.05		
Source of Variation	% of total variation	P value	P value summary
Interaction	0.6348	0.9361	ns
Row Factor	0.09516	0.8768	ns
Column Factor	86.29	<0.0001	****

Tukey's multiple comparisons test	Summary	Adjusted P Value
0		
Ctrl vs. c-Src-WT	ns	0.9649
Ctrl vs. c-Src-CA	****	<0.0001
Ctrl vs. c-Src-DN	ns	0.9158
c-Src-WT vs. c-Src-CA	****	<0.0001
c-Src-WT vs. c-Src-DN	ns	0.9980
c-Src-CA vs. c-Src-DN	****	<0.0001
5		
Ctrl vs. c-Src-WT	ns	0.9774
Ctrl vs. c-Src-CA	****	<0.0001
Ctrl vs. c-Src-DN	ns	0.9999
c-Src-WT vs. c-Src-CA	****	<0.0001
c-Src-WT vs. c-Src-DN	ns	0.9866
c-Src-CA vs. c-Src-DN	****	<0.0001
15		
Ctrl vs. c-Src-WT	ns	0.9638
Ctrl vs. c-Src-CA	****	<0.0001
Ctrl vs. c-Src-DN	ns	0.9954
c-Src-WT vs. c-Src-CA	****	<0.0001
c-Src-WT vs. c-Src-DN	ns	0.9949
c-Src-CA vs. c-Src-DN	****	<0.0001
Ctrl		
0 vs. 5	ns	0.9113
0 vs. 15	ns	0.9742
5 vs. 15	ns	0.9796
c-Src-WT		
0 vs. 5	ns	0.9371
0 vs. 15	ns	0.9730
5 vs. 15	ns	0.9920
c-Src-CA		
0 vs. 5	ns	0.7447
0 vs. 15	ns	0.8415
5 vs. 15	ns	0.4069
c-Src-DN		
0 vs. 5	ns	0.9843
0 vs. 15	ns	0.9790
5 vs. 15	ns	0.9996

Fig. 3D. pVE-cadherin Y731

Two-way ANOVA	Ordinary		
Alpha	0.05		
Source of Variation	% of total variation	P value	P value summary
Interaction	0.7640	0.9962	ns
Row Factor	0.2847	0.8991	ns
Column Factor	66.99	<0.0001	****

Tukey's multiple comparisons test	Summary	Adjusted P Value
0		
Ctrl vs. c-Src-WT	ns	0.9884
Ctrl vs. c-Src-CA	*	0.0122
Ctrl vs. c-Src-DN	ns	>0.9999
c-Src-WT vs. c-Src-CA	*	0.0255
c-Src-WT vs. c-Src-DN	ns	0.9880
c-Src-CA vs. c-Src-DN	*	0.0121
5		
Ctrl vs. c-Src-WT	ns	0.9705

Journal of Cell Science • Supplementary information

Ctrl vs. c-Src-CA	**	0.0038
Ctrl vs. c-Src-DN	ns	0.9999
c-Src-WT vs. c-Src-CA	*	0.0109
c-Src-WT vs. c-Src-DN	ns	0.9562
c-Src-CA vs. c-Src-DN	**	0.0032
15		
Ctrl vs. c-Src-WT	ns	0.9482
Ctrl vs. c-Src-CA	*	0.0254
Ctrl vs. c-Src-DN	ns	0.9998
c-Src-WT vs. c-Src-CA	ns	0.0809
c-Src-WT vs. c-Src-DN	ns	0.9235
c-Src-CA vs. c-Src-DN	*	0.0210
Ctrl		
0 vs. 5	ns	0.9972
0 vs. 15	ns	0.9979
5 vs. 15	ns	>0.9999
c-Src-WT		
0 vs. 5	ns	0.9795
0 vs. 15	ns	0.9571
5 vs. 15	ns	0.9957
c-Src-CA		
0 vs. 5	ns	0.8426
0 vs. 15	ns	0.9643
5 vs. 15	ns	0.6966
c-Src-DN		
0 vs. 5	ns	>0.9999
0 vs. 15	ns	0.9998
5 vs. 15	ns	0.9996

Fig. 3F. Average velocity

**Kruskal-Wallis test**	
P value	<0.0001
Exact or approximate P value?	Approximate
P value summary	****
Do the medians vary signif. (P < 0.05)?	Yes
Number of groups	4
Kruskal-Wallis statistic	25.81
Data summary	
Number of treatments (columns)	4
Number of values (total)	125

**Dunn's multiple comparisons test**	**Summary**	**Adjusted P Value**
Ctrl vs. c-Src-WT	ns	0.1088
Ctrl vs. c-Src-CA	**	0.0091
Ctrl vs. c-Src-DN	ns	>0.9999
c-Src-WT vs. c-Src-CA	ns	>0.9999
c-Src-WT vs. c-Src-DN	**	0.0020
c-Src-CA vs. c-Src-DN	****	<0.0001

Fig. 3G. Total distance

**Kruskal-Wallis test**	
P value	0.0205
Exact or approximate P value?	Approximate
P value summary	*
Do the medians vary signif. (P < 0.05)?	Yes

Number of groups	4
Kruskal-Wallis statistic	9.787

Dunn's multiple comparisons test	Summary	Adjusted P Value
Ctrl vs. c-Src-WT	ns	>0.9999
Ctrl vs. c-Src-CA	*	0.0402
Ctrl vs. c-Src-DN	ns	>0.9999
c-Src-WT vs. c-Src-CA	ns	0.6172
c-Src-WT vs. c-Src-DN	ns	>0.9999
c-Src-CA vs. c-Src-DN	ns	0.0619

Fig. 4B. Fibronectin fibrils

Kruskal-Wallis test	
P value	<0.0001
Exact or approximate P value?	Approximate
P value summary	****
Do the medians vary signif. (P < 0.05)?	Yes
Number of groups	4
Kruskal-Wallis statistic	25.33

Dunn's multiple comparisons test	Summary	Adjusted P Value
Ctrl vs. c-Src-WT	ns	>0.9999
Ctrl vs. c-Src-CA	***	0.0002
Ctrl vs. c-Src-DN	ns	>0.9999
c-Src-WT vs. c-Src-CA	***	0.0002
c-Src-WT vs. c-Src-DN	ns	>0.9999
c-Src-CA vs. c-Src-DN	***	0.0004

Fig. 5B. Fibronectin fibrils

Kruskal-Wallis test	
P value	0.2494
Exact or approximate P value?	Approximate
P value summary	ns
Do the medians vary signif. (P < 0.05)?	No
Number of groups	4
Kruskal-Wallis statistic	4.114

Dunn's multiple comparisons test	Summary	Adjusted P Value
Ctrl vs. c-Src-WT	ns	>0.9999
Ctrl vs. c-Src-CA	ns	>0.9999
Ctrl vs. c-Src-DN	ns	0.3850
c-Src-WT vs. c-Src-CA	ns	>0.9999
c-Src-WT vs. c-Src-DN	ns	0.5923
c-Src-CA vs. c-Src-DN	ns	>0.9999

Fig. 5D. Outgrowth composition

Two-way ANOVA	Ordinary		
Alpha	0.05		
Source of Variation	% of total variation	P value	P value summary
Interaction	77.16	<0.0001	****
Row Factor	0.3954	0.0220	*
Column Factor	18.03	<0.0001	****

Tukey's multiple comparisons test	Summary	Adjusted P Value
EV		
Sprout vs. Balloon	****	<0.0001
Src WT		
Sprout vs. Balloon	****	<0.0001
Src-CA		
Sprout vs. Balloon	****	<0.0001
Src DN		
Sprout vs. Balloon	****	<0.0001
Sprout		
EV vs. Src WT	*	0.0156
EV vs. Src-CA	****	<0.0001
EV vs. Src DN	ns	0.4411
Src WT vs. Src-CA	****	<0.0001
Src WT vs. Src DN	ns	0.5056
Src-CA vs. Src DN	****	<0.0001
Balloon		
EV vs. Src WT	ns	>0.9999
EV vs. Src-CA	****	<0.0001
EV vs. Src DN	ns	>0.9999
Src WT vs. Src-CA	****	<0.0001
Src WT vs. Src DN	ns	>0.9999
Src-CA vs. Src DN	****	<0.0001

Fig. 6B. Vascular area with marimastat

Two-way ANOVA	Ordinary		
Alpha	0.05		
Source of Variation	% of total variation	P value	P value summary
Interaction	18.90	<0.0001	****
Row Factor	57.34	<0.0001	****
Column Factor	8.371	<0.0001	****

Tukey's multiple comparisons test	Summary	Adjusted P Value
Ctrl		
DMSO vs. Marimastat	ns	0.6643
c-Src-WT		
DMSO vs. Marimastat	ns	0.4461
c-Src-CA		
DMSO vs. Marimastat	****	<0.0001
c-Src-DN		
DMSO vs. Marimastat	ns	0.9476
DMSO		
Ctrl vs. c-Src-WT	ns	0.9976
Ctrl vs. c-Src-CA	****	<0.0001
Ctrl vs. c-Src-DN	ns	0.9699
c-Src-WT vs. c-Src-CA	****	<0.0001
c-Src-WT vs. c-Src-DN	ns	0.9137
c-Src-CA vs. c-Src-DN	****	<0.0001
Marimastat		
Ctrl vs. c-Src-WT	ns	0.9998
Ctrl vs. c-Src-CA	****	<0.0001
Ctrl vs. c-Src-DN	ns	0.9995
c-Src-WT vs. c-Src-CA	****	<0.0001
c-Src-WT vs. c-Src-DN	ns	>0.9999
c-Src-CA vs. c-Src-DN	****	<0.0001

Journal of Cell Science • Supplementary information

Fig. 6C. Sprout number with marimastat

Two-way ANOVA	Ordinary		
Alpha	0.05		
Source of Variation	% of total variation	P value	P value summary
Interaction	17.04	<0.0001	****
Row Factor	32.37	<0.0001	****
Column Factor	13.47	<0.0001	****

Tukey's multiple comparisons test	Summary	Adjusted P Value
Ctrl		
DMSO vs. Marimastat	***	0.0003
c-Src-WT		
DMSO vs. Marimastat	****	<0.0001
c-Src-CA		
DMSO vs. Marimastat	ns	0.8389
c-Src-DN		
DMSO vs. Marimastat	ns	0.4144
DMSO		
Ctrl vs. c-Src-WT	***	0.0006
Ctrl vs. c-Src-CA	****	<0.0001
Ctrl vs. c-Src-DN	****	<0.0001
c-Src-WT vs. c-Src-CA	****	<0.0001
c-Src-WT vs. c-Src-DN	****	<0.0001
c-Src-CA vs. c-Src-DN	ns	0.4778
Marimastat		
Ctrl vs. c-Src-WT	ns	0.0971
Ctrl vs. c-Src-CA	**	0.0021
Ctrl vs. c-Src-DN	**	0.0082
c-Src-WT vs. c-Src-CA	ns	0.4668
c-Src-WT vs. c-Src-DN	ns	0.8007
c-Src-CA vs. c-Src-DN	ns	0.9298

Fig. 6D. Vascular area shape with marimastat

Two-way ANOVA	Ordinary		
Alpha	0.05		
Source of Variation	% of total variation	P value	P value summary
Interaction	11.68	<0.0001	****
Row Factor	14.72	<0.0001	****
Column Factor	21.62	<0.0001	****

Tukey's multiple comparisons test	Summary	Adjusted P Value
Ctrl		
DMSO vs. Marimastat	****	<0.0001
c-Src-WT		
DMSO vs. Marimastat	****	<0.0001
c-Src-CA		
DMSO vs. Marimastat	ns	0.7357
c-Src-DN		
DMSO vs. Marimastat	**	0.0076
DMSO		
Ctrl vs. c-Src-WT	ns	0.3028
Ctrl vs. c-Src-CA	*	0.0369
Ctrl vs. c-Src-DN	****	<0.0001
c-Src-WT vs. c-Src-CA	****	<0.0001
c-Src-WT vs. c-Src-DN	****	<0.0001

c-Src-CA vs. c-Src-DN	ns	0.0928
Marimastat		
Ctrl vs. c-Src-WT	ns	0.2963
Ctrl vs. c-Src-CA	ns	0.1677
Ctrl vs. c-Src-DN	*	0.0151
c-Src-WT vs. c-Src-CA	***	0.0008
c-Src-WT vs. c-Src-DN	ns	0.5577
c-Src-CA vs. c-Src-DN	****	<0.0001

Fig. 7B. Vessel width with marimastat

**Two-way ANOVA**	Ordinary		
Alpha	0.05		
Source of Variation	% of total variation	P value	P value summary
Interaction	24.77	<0.0001	****
Row Factor	59.11	<0.0001	****
Column Factor	15.17	<0.0001	****

**Tukey's multiple comparisons test**	**Summary**	**Adjusted P Value**
Ctrl		
DMSO vs. Marimastat	ns	0.5364
c-Src-WT		
DMSO vs. Marimastat	**	0.0072
c-Src-CA		
DMSO vs. Marimastat	****	<0.0001
c-Src-DN		
DMSO vs. Marimastat	*	0.0112
DMSO		
Ctrl vs. c-Src-WT	ns	0.7077
Ctrl vs. c-Src-CA	****	<0.0001
Ctrl vs. c-Src-DN	ns	0.9940
c-Src-WT vs. c-Src-CA	****	<0.0001
c-Src-WT vs. c-Src-DN	ns	0.8415
c-Src-CA vs. c-Src-DN	****	<0.0001
Marimastat		
Ctrl vs. c-Src-WT	**	0.0091
Ctrl vs. c-Src-CA	**	0.0020
Ctrl vs. c-Src-DN	ns	0.0817
c-Src-WT vs. c-Src-CA	****	<0.0001
c-Src-WT vs. c-Src-DN	ns	0.9733
c-Src-CA vs. c-Src-DN	****	<0.0001

Fig. 7C. cell number/vessel with marimastat

**Two-way ANOVA**	Ordinary		
Alpha	0.05		
Source of Variation	% of total variation	P value	P value summary
Interaction	6.478	0.4437	ns
Row Factor	40.67	0.0043	**
Column Factor	1.123	0.4944	ns

**Tukey's multiple comparisons test**	**Summary**	**Adjusted P Value**
Ctrl		
DMSO vs. Marimastat	ns	0.7606
c-Src-WT		
DMSO vs. Marimastat	ns	0.9152
c-Src-CA		
DMSO vs. Marimastat	ns	0.0855

Journal of Cell Science • Supplementary information

c-Src-DN		
DMSO vs. Marimastat	ns	0.9483
DMSO		
Ctrl vs. c-Src-WT	ns	0.9003
Ctrl vs. c-Src-CA	*	0.0279
Ctrl vs. c-Src-DN	ns	0.9973
c-Src-WT vs. c-Src-CA	**	0.0058
c-Src-WT vs. c-Src-DN	ns	0.9595
c-Src-CA vs. c-Src-DN	*	0.0181
Marimastat		
Ctrl vs. c-Src-WT	ns	0.8465
Ctrl vs. c-Src-CA	ns	0.7833
Ctrl vs. c-Src-DN	ns	0.9506
c-Src-WT vs. c-Src-CA	ns	0.3541
c-Src-WT vs. c-Src-DN	ns	0.9932
c-Src-CA vs. c-Src-DN	ns	0.5111

Fig. 7D. Vessel edge irregularity with marimastat

**Two-way ANOVA**	Ordinary		
Alpha	0.05		
Source of Variation	% of total variation	P value	P value summary
Interaction	2.023	0.7538	ns
Row Factor	1.195	0.8709	ns
Column Factor	0.4434	0.6102	ns

**Tukey's multiple comparisons test**	**Summary**	**Adjusted P Value**
Ctrl		
DMSO vs. Marimastat	ns	0.2443
c-Src-WT		
DMSO vs. Marimastat	ns	0.9675
c-Src-CA		
DMSO vs. Marimastat	ns	0.9350
c-Src-DN		
DMSO vs. Marimastat	ns	0.8308
DMSO		
Ctrl vs. c-Src-WT	ns	0.9956
Ctrl vs. c-Src-CA	ns	0.9966
Ctrl vs. c-Src-DN	ns	0.8544
c-Src-WT vs. c-Src-CA	ns	>0.9999
c-Src-WT vs. c-Src-DN	ns	0.9296
c-Src-CA vs. c-Src-DN	ns	0.9390
Marimastat		
Ctrl vs. c-Src-WT	ns	0.7381
Ctrl vs. c-Src-CA	ns	0.8044
Ctrl vs. c-Src-DN	ns	0.9298
c-Src-WT vs. c-Src-CA	ns	0.9994
c-Src-WT vs. c-Src-DN	ns	0.9776
c-Src-CA vs. c-Src-DN	ns	0.9917

Fig. 7E. Cell size with marimastat

**Two-way ANOVA**	Ordinary		
Alpha	0.05		
Source of Variation	% of total variation	P value	P value summary
Interaction	2.876	0.0127	*
Row Factor	7.550	<0.0001	****
Column Factor	3.098	0.0007	***

Journal of Cell Science • Supplementary information

Tukey's multiple comparisons test	Summary	Adjusted P Value
Ctrl		
DMSO vs. Marimastat	*	0.0283
c-Src-WT		
DMSO vs. Marimastat	ns	0.7533
c-Src-CA		
DMSO vs. Marimastat	ns	0.3786
c-Src-DN		
DMSO vs. Marimastat	****	<0.0001
DMSO		
Ctrl vs. c-Src-WT	*	0.0107
Ctrl vs. c-Src-CA	ns	0.9715
Ctrl vs. c-Src-DN	*	0.0451
c-Src-WT vs. c-Src-CA	**	0.0011
c-Src-WT vs. c-Src-DN	****	<0.0001
c-Src-CA vs. c-Src-DN	ns	0.0881
Marimastat		
Ctrl vs. c-Src-WT	ns	0.9523
Ctrl vs. c-Src-CA	ns	0.2769
Ctrl vs. c-Src-DN	ns	0.8759
c-Src-WT vs. c-Src-CA	ns	0.0924
c-Src-WT vs. c-Src-DN	ns	0.5661
c-Src-CA vs. c-Src-DN	ns	0.6974

Fig. 7F. Cell shape with marimastat

**Two-way ANOVA**	Ordinary		
Alpha	0.05		
Source of Variation	% of total variation	P value	P value summary
Interaction	0.8390	0.2688	ns
Row Factor	27.32	<0.0001	****
Column Factor	0.6291	0.0862	ns

Tukey's multiple comparisons test	Summary	Adjusted P Value
Ctrl		
DMSO vs. Marimastat	ns	0.6412
c-Src-WT		
DMSO vs. Marimastat	ns	0.1323
c-Src-CA		
DMSO vs. Marimastat	*	0.0338
c-Src-DN		
DMSO vs. Marimastat	ns	0.7351
DMSO		
Ctrl vs. c-Src-WT	ns	0.2919
Ctrl vs. c-Src-CA	****	<0.0001
Ctrl vs. c-Src-DN	*	0.0186
c-Src-WT vs. c-Src-CA	****	<0.0001
c-Src-WT vs. c-Src-DN	ns	0.6891
c-Src-CA vs. c-Src-DN	****	<0.0001
Marimastat		
Ctrl vs. c-Src-WT	ns	0.9951
Ctrl vs. c-Src-CA	****	<0.0001
Ctrl vs. c-Src-DN	ns	0.1861
c-Src-WT vs. c-Src-CA	****	<0.0001
c-Src-WT vs. c-Src-DN	ns	0.1101
c-Src-CA vs. c-Src-DN	***	0.0009

Fig. 7G. Vessel coverage with marimastat

Two-way ANOVA	Ordinary		
Alpha	0.05		
Source of Variation	% of total variation	P value	P value summary
Interaction	24.17	<0.0001	****
Row Factor	23.83	<0.0001	****
Column Factor	27.58	<0.0001	****

Tukey's multiple comparisons test	Summary	Adjusted P Value
Ctrl		
DMSO vs. Marimastat	ns	0.4150
c-Src-WT		
DMSO vs. Marimastat	ns	0.0759
c-Src-CA		
DMSO vs. Marimastat	****	<0.0001
c-Src-DN		
DMSO vs. Marimastat	*	0.0124
DMSO		
Ctrl vs. c-Src-WT	ns	0.6468
Ctrl vs. c-Src-CA	****	<0.0001
Ctrl vs. c-Src-DN	ns	0.3565
c-Src-WT vs. c-Src-CA	****	<0.0001
c-Src-WT vs. c-Src-DN	ns	0.9761
c-Src-CA vs. c-Src-DN	****	<0.0001
Marimastat		
Ctrl vs. c-Src-WT	ns	0.9892
Ctrl vs. c-Src-CA	ns	>0.9999
Ctrl vs. c-Src-DN	ns	>0.9999
c-Src-WT vs. c-Src-CA	ns	0.9949
c-Src-WT vs. c-Src-DN	ns	0.9877
c-Src-CA vs. c-Src-DN	ns	0.9999

Fig. S1E. c-Src expression level

Kruskal-Wallis test	
P value	0.0056
Exact or approximate P value?	Approximate
P value summary	**
Do the medians vary signif. (P < 0.05)?	Yes
Number of groups	4
Kruskal-Wallis statistic	12.61

Dunn's multiple comparisons test	Summary	Adjusted P Value
Ctrl vs. c-Src-WT	**	0.0057
Ctrl vs. c-Src-CA	ns	0.1335
Ctrl vs. c-Src-DN	*	0.0374
c-Src-WT vs. c-Src-CA	ns	>0.9999
c-Src-WT vs. c-Src-DN	ns	>0.9999
c-Src-CA vs. c-Src-DN	ns	>0.9999

Fig. S1G. DLL4 expression level

Kruskal-Wallis test	
P value	0.5386
Exact or approximate P value?	Exact

Journal of Cell Science • Supplementary information

P value summary	ns
Do the medians vary signif. (P < 0.05)?	No
Number of groups	4
Kruskal-Wallis statistic	2.385

Dunn's multiple comparisons test	Summary	Adjusted P Value
Ctrl vs. c-Src-WT	ns	>0.9999
Ctrl vs. c-Src-CA	ns	0.8462
Ctrl vs. c-Src-DN	ns	>0.9999
c-Src-WT vs. c-Src-CA	ns	>0.9999
c-Src-WT vs. c-Src-DN	ns	>0.9999
c-Src-CA vs. c-Src-DN	ns	>0.9999

Fig. S1I. Vessel width (y-axis)

Kruskal-Wallis test	
P value	0.0060
Exact or approximate P value?	Approximate
P value summary	**
Do the medians vary signif. (P < 0.05)?	Yes
Number of groups	4
Kruskal-Wallis statistic	12.45

Dunn's multiple comparisons test	Summary	Adjusted P Value
Ctrl vs. c-Src-WT	ns	0.4220
Ctrl vs. c-Src-CA	**	0.0029
Ctrl vs. c-Src-DN	ns	>0.9999
c-Src-WT vs. c-Src-CA	ns	0.5105
c-Src-WT vs. c-Src-DN	ns	>0.9999
c-Src-CA vs. c-Src-DN	ns	0.1960

Fig. S1K. Ki-67 Proliferation

Kruskal-Wallis test	
P value	0.5630
Exact or approximate P value?	Approximate
P value summary	ns
Do the medians vary signif. (P < 0.05)?	No
Number of groups	4
Kruskal-Wallis statistic	2.046

Dunn's multiple comparisons test	Summary	Adjusted P Value
Ctrl vs. c-Src-WT	ns	>0.9999
Ctrl vs. c-Src-CA	ns	>0.9999
Ctrl vs. c-Src-DN	ns	>0.9999
c-Src-WT vs. c-Src-CA	ns	>0.9999
c-Src-WT vs. c-Src-DN	ns	>0.9999
c-Src-CA vs. c-Src-DN	ns	>0.9999

Fig. S1M. EdU Proliferation

Kruskal-Wallis test	
P value	0.0060
Exact or approximate P value?	Approximate
P value summary	***
Do the medians vary signif. (P < 0.05)?	Yes
Number of groups	4
Kruskal-Wallis statistic	17.22

Dunn's multiple comparisons test	Summary	Adjusted P Value
Ctrl vs. c-Src-WT	ns	0.1495
Ctrl vs. c-Src-CA	**	0.0010
Ctrl vs. c-Src-DN	ns	>0.9999
c-Src-WT vs. c-Src-CA	ns	0.7770
c-Src-WT vs. c-Src-DN	ns	0.6106
c-Src-CA vs. c-Src-DN	**	0.0097

Fig. S2B. Ki-67 Proliferation

Kruskal-Wallis test	
P value	0.9806
Exact or approximate P value?	Exact
P value summary	ns
Do the medians vary signif. (P < 0.05)?	No
Number of groups	4
Kruskal-Wallis statistic	0.2078

Dunn's multiple comparisons test	Summary	Adjusted P Value
Ctrl vs. c-Src-WT	ns	>0.9999
Ctrl vs. c-Src-CA	ns	>0.9999
Ctrl vs. c-Src-DN	ns	>0.9999
c-Src-WT vs. c-Src-CA	ns	>0.9999
c-Src-WT vs. c-Src-DN	ns	>0.9999
c-Src-CA vs. c-Src-DN	ns	>0.9999

Fig. S2D. EdU Proliferation

Kruskal-Wallis test	
P value	0.1649
Exact or approximate P value?	Exact
P value summary	ns
Do the medians vary signif. (P < 0.05)?	No
Number of groups	4
Kruskal-Wallis statistic	5.088

Dunn's multiple comparisons test	Summary	Adjusted P Value
Ctrl vs. c-Src-WT	ns	>0.9999
Ctrl vs. c-Src-CA	ns	0.3311
Ctrl vs. c-Src-DN	ns	0.5394
c-Src-WT vs. c-Src-CA	ns	0.9530
c-Src-WT vs. c-Src-DN	ns	>0.9999
c-Src-CA vs. c-Src-DN	ns	>0.9999

Fig. S2F. Cell size

Kruskal-Wallis test	
P value	0.3356
Exact or approximate P value?	Approximate
P value summary	ns
Do the medians vary signif. (P < 0.05)?	No
Number of groups	4
Kruskal-Wallis statistic	3.388

Dunn's multiple comparisons test	Summary	Adjusted P Value
Ctrl vs. c-Src-WT	ns	>0.9999

Journal of Cell Science • Supplementary information

Ctrl vs. c-Src-CA	ns	0.7235
Ctrl vs. c-Src-DN	ns	>0.9999
c-Src-WT vs. c-Src-CA	ns	0.5556
c-Src-WT vs. c-Src-DN	ns	>0.9999
c-Src-CA vs. c-Src-DN	ns	>0.9999

Fig. S2G. Cell perimeter

Kruskal-Wallis test	
P value	0.0151
Exact or approximate P value?	Approximate
P value summary	*
Do the medians vary signif. (P < 0.05)?	Yes
Number of groups	4
Kruskal-Wallis statistic	10.44

Dunn's multiple comparisons test	Summary	Adjusted P Value
Ctrl vs. c-Src-WT	ns	>0.9999
Ctrl vs. c-Src-CA	ns	0.0698
Ctrl vs. c-Src-DN	ns	>0.9999
c-Src-WT vs. c-Src-CA	**	0.0085
c-Src-WT vs. c-Src-DN	ns	>0.9999
c-Src-CA vs. c-Src-DN	ns	0.1747

Fig. S2H. Cell Shape

Kruskal-Wallis test	
P value	0.0101
Exact or approximate P value?	Approximate
P value summary	*
Do the medians vary signif. (P < 0.05)?	Yes
Number of groups	4
Kruskal-Wallis statistic	11.33

Dunn's multiple comparisons test	Summary	Adjusted P Value
Ctrl vs. c-Src-WT	ns	0.1545
Ctrl vs. c-Src-CA	*	0.0191
Ctrl vs. c-Src-DN	ns	>0.9999
c-Src-WT vs. c-Src-CA	ns	>0.9999
c-Src-WT vs. c-Src-DN	ns	0.7411
c-Src-CA vs. c-Src-DN	ns	0.0955

Fig. S2J. Cleaved Caspase3 Apoptosis

Kruskal-Wallis test	
P value	0.1112
Exact or approximate P value?	Exact
P value summary	ns
Do the medians vary signif. (P < 0.05)?	No
Number of groups	4
Kruskal-Wallis statistic	5.821

Dunn's multiple comparisons test	Summary	Adjusted P Value
Ctrl vs. c-Src-WT	ns	0.1412
Ctrl vs. c-Src-CA	ns	>0.9999
Ctrl vs. c-Src-DN	ns	0.4202
c-Src-WT vs. c-Src-CA	ns	>0.9999
c-Src-WT vs. c-Src-DN	ns	>0.9999

Journal of Cell Science • Supplementary information

c-Src-CA vs. c-Src-DN	ns	>0.9999

Fig. S3B. p-paxillin Y118

Kruskal-Wallis test	
P value	0.0027
Exact or approximate P value?	Approximate
P value summary	**
Do the medians vary signif. (P < 0.05)?	Yes
Number of groups	4
Kruskal-Wallis statistic	14.15

Dunn's multiple comparisons test	Summary	Adjusted P Value
Ctrl vs. c-Src-WT	ns	0.4150
Ctrl vs. c-Src-CA	**	0.0031
Ctrl vs. c-Src-DN	ns	>0.9999
c-Src-WT vs. c-Src-CA	ns	0.5851
c-Src-WT vs. c-Src-DN	ns	>0.9999
c-Src-CA vs. c-Src-DN	*	0.0234

Fig. S3C. pFAK Y576

Kruskal-Wallis test	
P value	0.0313
Exact or approximate P value?	Approximate
P value summary	*
Do the medians vary signif. (P < 0.05)?	Yes
Number of groups	4
Kruskal-Wallis statistic	8.851

Dunn's multiple comparisons test	Summary	Adjusted P Value
Ctrl vs. c-Src-WT	ns	>0.9999
Ctrl vs. c-Src-CA	*	0.0197
Ctrl vs. c-Src-DN	ns	>0.9999
c-Src-WT vs. c-Src-CA	ns	0.4665
c-Src-WT vs. c-Src-DN	ns	>0.9999
c-Src-CA vs. c-Src-DN	ns	0.4150

Fig. S3E. FA size

Kruskal-Wallis test	
P value	<0.0001
Exact or approximate P value?	Approximate
P value summary	****
Do the medians vary signif. (P < 0.05)?	Yes
Number of groups	4
Kruskal-Wallis statistic	27.90

Dunn's multiple comparisons test	Summary	Adjusted P Value
Ctrl vs. c-Src-WT	ns	>0.9999
Ctrl vs. c-Src-CA	****	<0.0001
Ctrl vs. c-Src-DN	***	0.0007
c-Src-WT vs. c-Src-CA	**	0.0071
c-Src-WT vs. c-Src-DN	*	0.0369
c-Src-CA vs. c-Src-DN	ns	>0.9999

Journal of Cell Science • Supplementary information

Fig. S3F. FA number

Kruskal-Wallis test	
P value	0.0078
Exact or approximate P value?	Approximate
P value summary	**
Do the medians vary signif. (P < 0.05)?	Yes
Number of groups	4
Kruskal-Wallis statistic	11.89

Dunn's multiple comparisons test	Summary	Adjusted P Value
Ctrl vs. c-Src-WT	ns	0.0746
Ctrl vs. c-Src-CA	**	0.0097
Ctrl vs. c-Src-DN	ns	>0.9999
c-Src-WT vs. c-Src-CA	ns	>0.9999
c-Src-WT vs. c-Src-DN	ns	>0.9999
c-Src-CA vs. c-Src-DN	ns	0.3376

Fig. S3G. FA density

Kruskal-Wallis test	
P value	0.0005
Exact or approximate P value?	Approximate
P value summary	***
Do the medians vary signif. (P < 0.05)?	Yes
Number of groups	4
Kruskal-Wallis statistic	17.90

Dunn's multiple comparisons test	Summary	Adjusted P Value
Ctrl vs. c-Src-WT	ns	0.5772
Ctrl vs. c-Src-CA	***	0.0008
Ctrl vs. c-Src-DN	ns	>0.9999
c-Src-WT vs. c-Src-CA	ns	0.1588
c-Src-WT vs. c-Src-DN	ns	0.8570
c-Src-CA vs. c-Src-DN	**	0.0053

Fig. S4B. Average velocity

Kruskal-Wallis test	
P value	<0.0001
Exact or approximate P value?	Approximate
P value summary	****
Do the medians vary signif. (P < 0.05)?	Yes
Number of groups	4
Kruskal-Wallis statistic	57.49

Dunn's multiple comparisons test	Summary	Adjusted P Value
Ctrl vs. c-Src-WT	ns	0.1439
Ctrl vs. c-Src-CA	****	<0.0001
Ctrl vs. c-Src-DN	ns	>0.9999
c-Src-WT vs. c-Src-CA	****	<0.0001
c-Src-WT vs. c-Src-DN	ns	0.0921
c-Src-CA vs. c-Src-DN	****	<0.0001

Fig. S4C. Total distance

Kruskal-Wallis test	
P value	<0.0001
Exact or approximate P value?	Approximate

P value summary	****
Do the medians vary signif. (P < 0.05)?	Yes
Number of groups	4
Kruskal-Wallis statistic	57.88

**Dunn's multiple comparisons test**	**Summary**	**Adjusted P Value**
Ctrl vs. c-Src-WT	ns	0.1288
Ctrl vs. c-Src-CA	****	<0.0001
Ctrl vs. c-Src-DN	ns	>0.9999
c-Src-WT vs. c-Src-CA	****	<0.0001
c-Src-WT vs. c-Src-DN	ns	0.0704
c-Src-CA vs. c-Src-DN	****	<0.0001

Fig. S5B. Fibronectin fibrils

**Two-way ANOVA**	Ordinary		
Alpha	0.05		
Source of Variation	% of total variation	P value	P value summary
Interaction	9.737	<0.0001	****
Row Factor	32.09	<0.0001	****
Column Factor	23.11	<0.0001	****

**Tukey's multiple comparisons test**	**Summary**	**Adjusted P Value**
Ctrl		
4Hr vs. 24Hr	****	<0.0001
4Hr vs. 48Hr	****	<0.0001
24Hr vs. 48Hr	**	0.0032
c-Src-WT		
4Hr vs. 24Hr	****	<0.0001
4Hr vs. 48Hr	****	<0.0001
24Hr vs. 48Hr	ns	0.1625
c-Src-CA		
4Hr vs. 24Hr	ns	0.8596
4Hr vs. 48Hr	ns	0.6106
24Hr vs. 48Hr	ns	0.9045
c-Src-DN		
4Hr vs. 24Hr	****	<0.0001
4Hr vs. 48Hr	***	0.0004
24Hr vs. 48Hr	ns	0.5481
4Hr		
Ctrl vs. c-Src-WT	**	0.0054
Ctrl vs. c-Src-CA	*	0.0260
Ctrl vs. c-Src-DN	**	0.0033
c-Src-WT vs. c-Src-CA	ns	0.9414
c-Src-WT vs. c-Src-DN	ns	0.9988
c-Src-CA vs. c-Src-DN	ns	0.8897
24Hr		
Ctrl vs. c-Src-WT	ns	0.4307
Ctrl vs. c-Src-CA	****	<0.0001
Ctrl vs. c-Src-DN	***	0.0008
c-Src-WT vs. c-Src-CA	****	<0.0001
c-Src-WT vs. c-Src-DN	ns	0.0636
c-Src-CA vs. c-Src-DN	**	0.0020
48Hr		
Ctrl vs. c-Src-WT	****	<0.0001
Ctrl vs. c-Src-CA	****	<0.0001
Ctrl vs. c-Src-DN	****	<0.0001

Journal of Cell Science • Supplementary information

c-Src-WT vs. c-Src-CA	**	0.0017
c-Src-WT vs. c-Src-DN	ns	0.3580
c-Src-CA vs. c-Src-DN	ns	0.1337

Fig. S6B. Fibronectin fibrils

**Two-way ANOVA**	Ordinary		
Alpha	0.05		
Source of Variation	% of total variation	P value	P value summary
Interaction	6.544	0.0472	*
Row Factor	10.81	0.0051	**
Column Factor	2.088	0.1085	ns

**Tukey's multiple comparisons test**	**Summary**	**Adjusted P Value**
Ctrl		
DMSO vs. Marimastat	ns	0.1677
c-Src-WT		
DMSO vs. Marimastat	ns	0.6641
c-Src-CA		
DMSO vs. Marimastat	ns	0.0812
c-Src-DN		
DMSO vs. Marimastat	*	0.0269
DMSO		
Ctrl vs. c-Src-WT	ns	0.1209
Ctrl vs. c-Src-CA	***	0.0007
Ctrl vs. c-Src-DN	*	0.0107
c-Src-WT vs. c-Src-CA	ns	0.2381
c-Src-WT vs. c-Src-DN	ns	0.7436
c-Src-CA vs. c-Src-DN	ns	0.8186
Marimastat		
Ctrl vs. c-Src-WT	ns	0.9712
Ctrl vs. c-Src-CA	ns	0.7164
Ctrl vs. c-Src-DN	ns	0.9716
c-Src-WT vs. c-Src-CA	ns	0.9268
c-Src-WT vs. c-Src-DN	ns	0.8193
c-Src-CA vs. c-Src-DN	ns	0.4549

Fig. S6C. FA size

**Two-way ANOVA**	Ordinary		
Alpha	0.05		
Source of Variation	% of total variation	P value	P value summary
Interaction	0.04728	0.9414	ns
Row Factor	64.94	<0.0001	****
Column Factor	0.2157	0.1812	ns

**Tukey's multiple comparisons test**	**Summary**	**Adjusted P Value**
Ctrl		
DMSO vs. Marimastat	ns	0.2303
c-Src-WT		
DMSO vs. Marimastat	ns	0.4557
c-Src-CA		
DMSO vs. Marimastat	ns	0.8668
c-Src-DN		
DMSO vs. Marimastat	ns	0.4849
DMSO		
Ctrl vs. c-Src-WT	ns	>0.9999

Ctrl vs. c-Src-CA	****	<0.0001
Ctrl vs. c-Src-DN	ns	0.8488
c-Src-WT vs. c-Src-CA	****	<0.0001
c-Src-WT vs. c-Src-DN	ns	0.8516
c-Src-CA vs. c-Src-DN	****	<0.0001
Marimastat		
Ctrl vs. c-Src-WT	ns	0.9684
Ctrl vs. c-Src-CA	****	<0.0001
Ctrl vs. c-Src-DN	ns	0.9466
c-Src-WT vs. c-Src-CA	****	<0.0001
c-Src-WT vs. c-Src-DN	ns	0.7384
c-Src-CA vs. c-Src-DN	****	<0.0001

Fig. S6D. FA number

**Two-way ANOVA**	Ordinary		
Alpha	0.05		
Source of Variation	% of total variation	P value	P value summary
Interaction	1.653	0.0946	ns
Row Factor	22.70	<0.0001	****
Column Factor	1.173	0.0334	*

**Tukey's multiple comparisons test**	**Summary**	**Adjusted P Value**
Ctrl		
DMSO vs. Marimastat	*	0.0201
c-Src-WT		
DMSO vs. Marimastat	ns	0.7923
c-Src-CA		
DMSO vs. Marimastat	*	0.0197
c-Src-DN		
DMSO vs. Marimastat	ns	0.5732
DMSO		
Ctrl vs. c-Src-WT	**	0.0036
Ctrl vs. c-Src-CA	**	0.0022
Ctrl vs. c-Src-DN	ns	0.3788
c-Src-WT vs. c-Src-CA	ns	0.8872
c-Src-WT vs. c-Src-DN	***	0.0001
c-Src-CA vs. c-Src-DN	****	<0.0001
Marimastat		
Ctrl vs. c-Src-WT	ns	0.6828
Ctrl vs. c-Src-CA	***	0.0002
Ctrl vs. c-Src-DN	****	<0.0001
c-Src-WT vs. c-Src-CA	**	0.0073
c-Src-WT vs. c-Src-DN	****	<0.0001
c-Src-CA vs. c-Src-DN	****	<0.0001

Fig. S6E. FA density

**Two-way ANOVA**	Ordinary		
Alpha	0.05		
Source of Variation	% of total variation	P value	P value summary
Interaction	0.1602	0.6785	ns
Row Factor	69.23	<0.0001	****
Column Factor	0.2635	0.1153	ns

**Tukey's multiple comparisons test**	**Summary**	**Adjusted P Value**
Ctrl		
DMSO vs. Marimastat	ns	0.9614

c-Src-WT		
DMSO vs. Marimastat	ns	0.1997
c-Src-CA		
DMSO vs. Marimastat	ns	0.1672
c-Src-DN		
DMSO vs. Marimastat	ns	0.6231
DMSO		
Ctrl vs. c-Src-WT	ns	0.0920
Ctrl vs. c-Src-CA	****	<0.0001
Ctrl vs. c-Src-DN	ns	0.5690
c-Src-WT vs. c-Src-CA	****	<0.0001
c-Src-WT vs. c-Src-DN	*	0.0107
c-Src-CA vs. c-Src-DN	****	<0.0001
Marimastat		
Ctrl vs. c-Src-WT	ns	0.8376
Ctrl vs. c-Src-CA	****	<0.0001
Ctrl vs. c-Src-DN	ns	0.2090
c-Src-WT vs. c-Src-CA	****	<0.0001
c-Src-WT vs. c-Src-DN	*	0.0244
c-Src-CA vs. c-Src-DN	****	<0.0001

Fig. S7B. Vessel width (y-axis)

Two-way ANOVA	Ordinary		
Alpha	0.05		
Source of Variation	% of total variation	P value	P value summary
Interaction	16.49	0.0022	**
Row Factor	50.68	<0.0001	****
Column Factor	9.551	0.0027	**

**Tukey's multiple comparisons test**	**Summary**	**Adjusted P Value**
Ctrl		
DMSO vs. Marimastat	ns	0.5213
c-Src-WT		
DMSO vs. Marimastat	ns	0.6626
c-Src-CA		
DMSO vs. Marimastat	****	<0.0001
c-Src-DN		
DMSO vs. Marimastat	ns	0.6811
DMSO		
Ctrl vs. c-Src-WT	ns	0.7933
Ctrl vs. c-Src-CA	****	<0.0001
Ctrl vs. c-Src-DN	ns	0.9326
c-Src-WT vs. c-Src-CA	****	<0.0001
c-Src-WT vs. c-Src-DN	ns	0.9898
c-Src-CA vs. c-Src-DN	****	<0.0001
Marimastat		
Ctrl vs. c-Src-WT	ns	0.8037
Ctrl vs. c-Src-CA	ns	0.4743
Ctrl vs. c-Src-DN	ns	0.9582
c-Src-WT vs. c-Src-CA	ns	0.1220
c-Src-WT vs. c-Src-DN	ns	0.9808
c-Src-CA vs. c-Src-DN	ns	0.2562

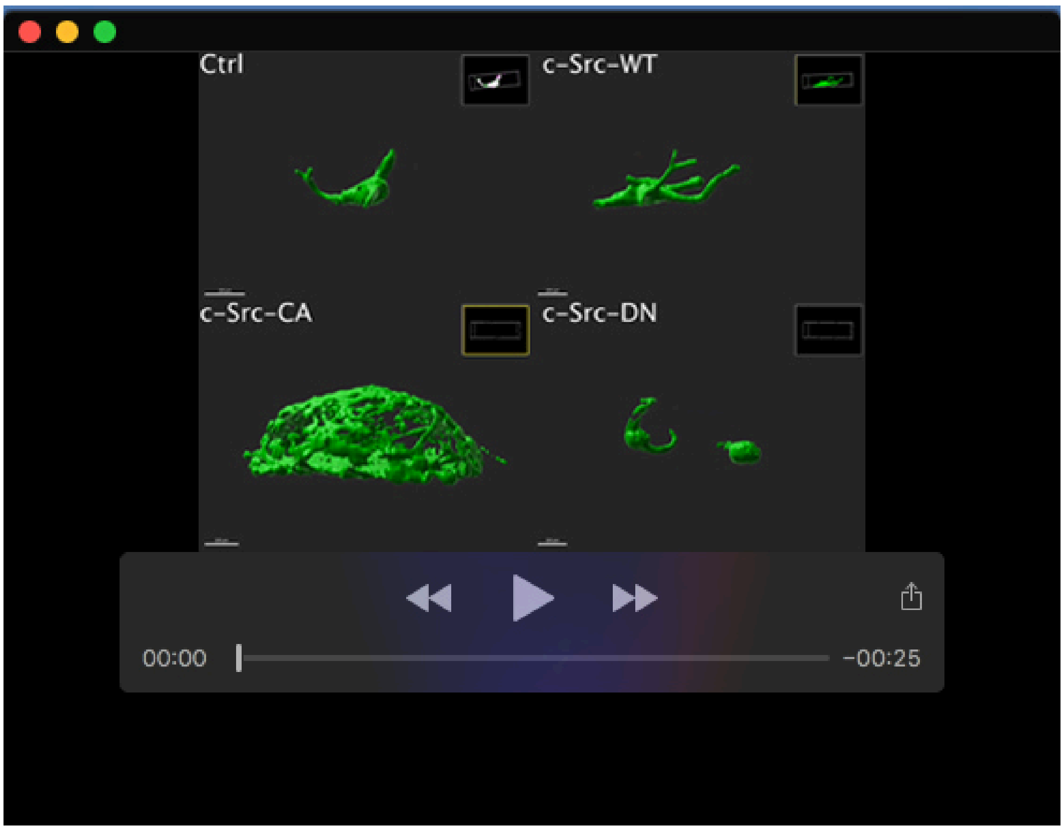

Movie 1. Constitutively active c-Src induces ballooning vasculature in a bead sprouting assay. Corresponds to Figure 1A. HUVECs transduced with c-Src-mScarlet mutants were grown in a 5 mg/mL fibrin gel bead sprouting assay for 7 days before fixing. Immunofluorescent staining for F-Actin (Phalloidin) and mScarlet signal were used to 3D render sprouts using Imaris software.

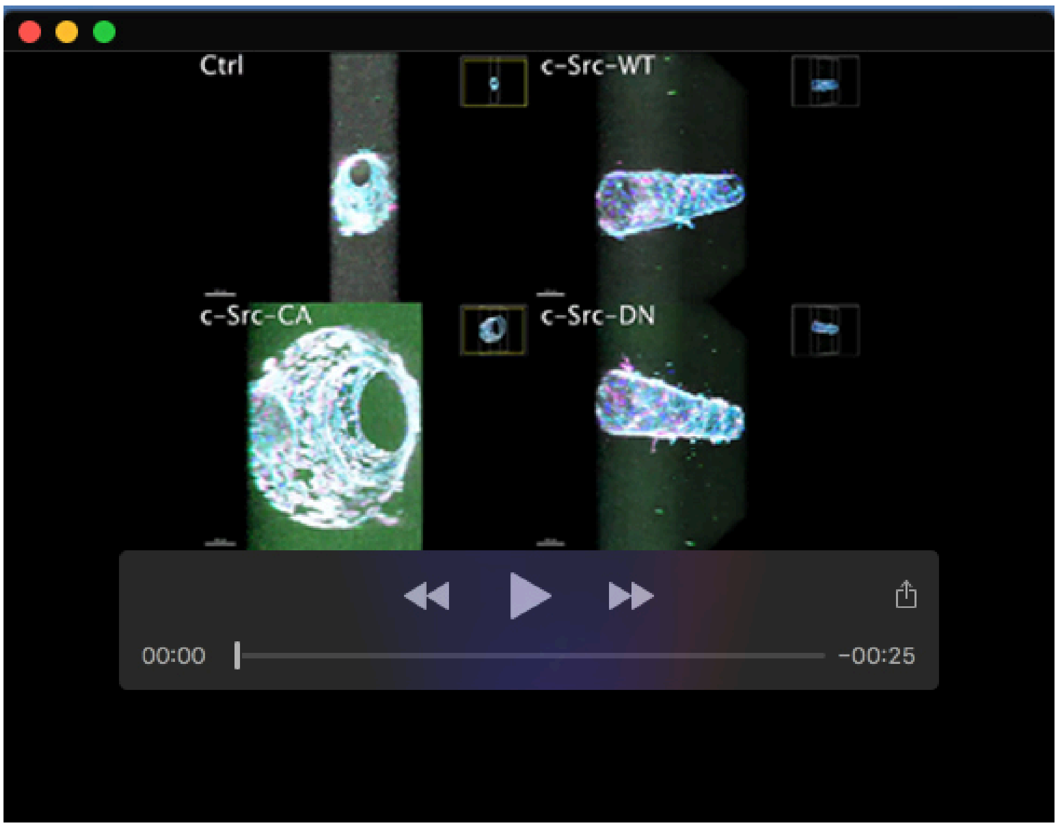

Movie 2. Constitutively active c-Src induces ballooning vasculature in 3D microvessels. Corresponds to Figure 1F. HUVECs transduced with mScarlet-tagged c-Src mutants (magenta) grown in PDMS microfluidic vessels containing 2.5 mg/mL collagen matrix for 3 days before fixing. Immunofluorescent staining was performed for VE-Cadherin (green), F-Actin (cyan), and nuclei (DAPI; blue). 3D projection was generated using Imaris software.

Journal of Cell Science • Supplementary information

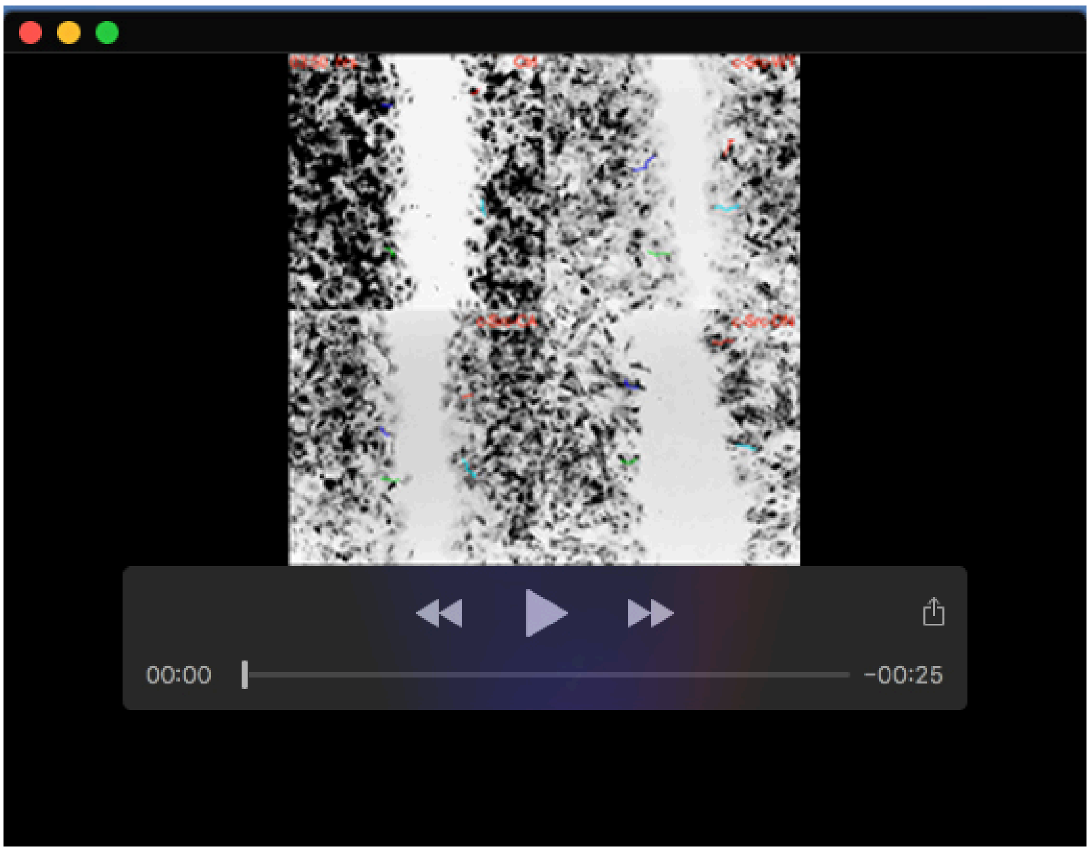

Movie 3. Constitutively active c-Src reduces cell migration in 2D. Corresponds to Supplementary Fig. 4A. HUVECs transduced with mScarlet-tagged c-Src mutants migrating in a scratch assay for live imaging of mScarlet (black) at 10 min interval over 15 h.

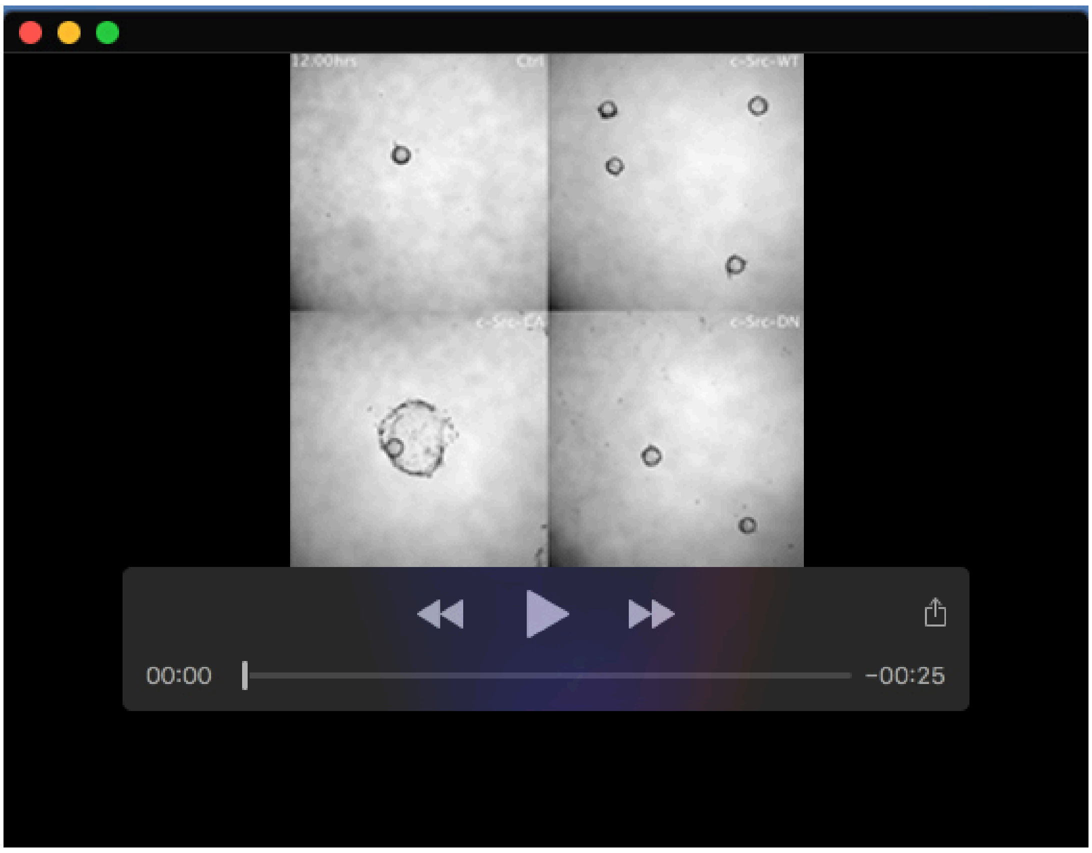

Movie 4. Constitutively active c-Src decreases 3D cell migration in a bead sprouting assay. Corresponds to Figure 3E. HUVECs transduced with mScarlet-tagged c-Src mutants were grown in a 5 mg/mL fibrin gel bead sprouting assay for 5 days before live imaging of DIC at 30 min intervals over 72 h.
