# Supplementary material for: c-Src-induced vascular malformations require localised matrix degradation at focal adhesions
Source: J Cell Sci. 2024 Jul 10;137(13):jcs262101. doi: 10.1242/jcs.262101 (PMC11267457; doi:10.1242/jcs.262101)
Supplement: Supplementary file 1 [file joces-137-262101_review_history.pdf]

c-Src-induced vascular malformations require localised matrix degradation at focal adhesions

Patricia Essebier, Mikaela Keyser, Teodor Yordanov, Brittany Hill, Alexander Yu, Ivar Noordstra, Alpha S. Yap, Samantha J. Stehbens, Anne K. Lagendijk, Lilian Schimmel and Emma J. Gordon
DOI: 10.1242/jcs.262101

Editor: Kairbaan Hodivala-Dilke

Review timeline

Submission to Review Commons:	15 September 2023
Submission to Journal of Cell Science:	7 March 2024
Editorial decision:	12 March 2024
First revision received:	28 May 2024
Accepted:	6 June 2024

Original submission

First decision letter

MS ID#: JOCES/2024/262101

MS TITLE: c-Src induced vascular malformations require localised matrix degradation at focal adhesions

AUTHORS: Patricia Essebier, Teodor Yordanov, Mikaela Keyser, Alexander Yu, Ivar Noordstra, Brittany Hill, Alpha S. Yap, Samantha J Stehbens, Anne Lagendijk, Lilian Schimmel, and Emma Gordon

We have now reached a decision on the above manuscript.

To see the reviewers' reports and a copy of this decision letter, please go to: https://submit-jcs.biologists.org and click on the 'Manuscripts with Decisions' queue in the Author Area. (Corresponding author only has access to reviews.)

As you will see, the reviewers raise a number of substantial criticisms that prevent me from accepting the paper at this stage. They suggest, however, that a revised version might prove acceptable, if you can address their concerns. If you think that you can deal satisfactorily with the criticisms on revision, I would be pleased to see a revised manuscript. We would then return it to the reviewers. In partcilular I agree that all your given plans for experiments are acceptable and appropriate. Some guidance for your responses to specific comments include:
Rev 1.3
More quantitative results would be required. Depending on staining alone might not be acceptable by the reviewers Western blotting or Q-PCR suggested.
Rev 3.2
Given plan of experiments is acceptable and appropriate, please give a reason why EDU incorporation, as suggested by the reviewer was not appropriate.
Rev 3. Minor point 1 – could the authors give separate stain images for some of the very overlapping stains in the supp figures, rather than not give any at all?
Rev 1.3

© 2024. Published by The Company of Biologists under the terms of the Creative Commons Attribution License (https://creativecommons.org/licenses/by/4.0/).

More quantitative results would be required. Depending on staining alone might not be acceptable by the reviewers Western blotting or Q-PCR suggested.
Rev 3.2
Given plan of experiments is acceptable and appropriate, please give a reason why EDU incorporation, as suggested by the reviewer was not appropriate.
Comment preferred not to address
Rev 3.5 – agree that written responses may suffice
Rev 3. Minor point 1 – could the authors give separate stain images for some of the very overlapping stains in the supp figures, rather than not give any at all?

Please ensure that you clearly highlight all changes made in the revised manuscript. Please avoid using 'Tracked changes' in Word files as these are lost in PDF conversion.

I should be grateful if you would also provide a point-by-point response detailing how you have dealt with the points raised by the reviewers in the 'Response to Reviewers' box. Please attend to all of the reviewers' comments. If you do not agree with any of their criticisms or suggestions please explain clearly why this is so.

Reviewer 1

Evidence, reproducibility and clarity

The manuscript demonstrates the effects of overexpression of c-Src variants in HUVEC endothelial cells. The c-Src kinase interacts with cell adhesion machinery, and the manuscript dissects relationships downstream of c-Src with respect to cellular behavior. Transduced wild type, constituent active, dominant negative c-Src is assayed by sprouting in 3D using a bead system, growth of microfabricated vessels under oscillatory flow, focal adhesion analysis, migration analysis, ECM analysis, and by rescue with a matrix Metalloprotease inhibitor.

Major comments:

1. p5. Fig 1: The sentence that the dominant negative completely abrogated 'this' phenotype implies that the dominant negative was put into the same cells as the constitutively active mutation. 'Abrogated' means it stops the phenotype, and the phenotype in the sentence prior was constitutively active. It is more accurate to say that the dominant negative was not distinguishable from wild type, which is what the statistics show. No double transfection (DN- CA) was performed.
2. p.5. Fig 1: the phenotype of the CA cells is fascinating. They expand far beyond their normal territory, but they are held together in a lacy bubble. To me, this looks like a different phenotype from the ballooning that might occur in an arteriovenous malformation in vivo, as in vivo malformations are continuously covered by cells. I understand why the authors might use the term ballooning but given that the cells expand without continuously touching each other, I do not think this is the correct term. Would blebbing, or radial migration in a lace-like discontinuous pattern describe it better?
3. p. 5. The authors do not describe any relationship to notch signaling. But notch signaling is the mechanism by which a sprout is selected. The CA phenotype shows no selection, and every sprout can continue migration. Did the authors check for any relationship between notch signaling c-Src activation? Does upregulation of C-Src downregulate notch?
4. The statistical methods are not described in the methods (GraphPad?). These need to be added. Are only significant comparisons plotted? In Fig 6 and 7 only pairwise statistics are shown. If all significant comparisons are plotted, then this means that the comparison between the rescued CA and the treated or untreated control is not significant. This can be thought of as a partial rescue towards a wild type, but it is definitely not a full rescue. None of the statistical comparisons in Figure 6 or 7 show significant comparisons to wildtype. This needs more discussion.
5. Mmp activity is inferred, but not measured. This is a limitaion as the assumption is that marimostat acting through the expected pathway.

Minor concerns:

© 2024. Published by The Company of Biologists under the terms of the Creative Commons Attribution License (https://creativecommons.org/licenses/by/4.0/).

1. Fig 5D. The presentation of the data in this graph is difficult to understand. It is trying to show the proportion of mScarlet in sprouts or balloons a percentage of all the scarlet cells. It would be better to have all cells represented in one bar, distributed between sprout and balloon in that one bar. i.e., for the control and dominant negative, the bars would be all black and then for the CA it would be all white. The zero data points are confusing. A proportions graph should be investigated here.
2. The methods for vessel coverage for quantification in figs 1 and 7 are missing.

Referees cross-commenting

The comments from the other reviewers seem reasonable.

Significance

The work is well executed and takes a mechanistic approach. The images are well put together and the movies significantly add to the manuscript. The phenotype describes highly unusual endothelial behavior, which is of interest, and an advance in the field for its novelty. Linking cSrc to downstream signalling including mmps and demonstrating a rescue is also novel and a strength. This is a conceptual advance in the relationship between a kinase and cell behaviour in 3D.

Understanding this mechanism may be useful in understanding enlarged vessels in vascular malformations, although the direct relevance is not clear due to limitations of using cultured cells in artificial environments, lacking, for instance, support by secondary cells and ECM that might be contributed by support cells and perhaps modulate the phenotype.

The audience would be specialized in the basic research community.

Reviewer 2

Evidence, reproducibility and clarity

In this work, Essebier and colleagues have shown that the upregulation of c-Src in endothelial cells results in vascular dilation independently of growth factors or shear stress. The authors have shown that this effect is driven by alteration in the number of focal adhesion and the secretion of matrix metalloproteinases responsible for extracellular matrix remodeling as the inhibition of the MMPs rescues the observed effects.

This is an elegant work, with well-designed experiments and nice images to illustrate them. Congratulations. Nevertheless, the results not really support the conclusions drawn by the authors. The authors have only used one type of vein endothelial cells from one single donor but they conclude that is effect is general for all endothelial cells. Endothelial cells are very heterogeneous, not only depending on their function and localization, vein, artery or capillary, but also between different organs and in disease (PMID: 22315715, PMID: 28775214, PMID: 31944177, PMID: 33514719).

The authors, should either repeat some of the key experiments in other type of endothelial cells, maybe arterial or microvasculature cells which are commercially available or at least state that the observations presented in this manuscript apply to HUVECs and discuss whether this would also apply for other cell types.
Minor. Although the methods are well written and can be understood. To improve transparency, the authors should reduce the referring to other papers to describe the methods they perform and at least some kind of brief description should be included.

The authors should report the real p value for their tests. Also when the test is not significant.

Referees cross-commenting

I agree with reviewer #1. Description of the statistical methods should be described in the methods. I have nothing else to add to the comments from the other reviewers.

© 2024. Published by The Company of Biologists under the terms of the Creative Commons Attribution License (https://creativecommons.org/licenses/by/4.0/).

Significance

The work presented here by Essebier and colleagues is very well designed and performed. The main strength of the manuscript is the study of the molecular mechanism that regulate the relationship between cells and the extracellular matrix. This is not very well studied in the context of disease. Although all the assays have been performed elegantly, the main limitation of this study is that it has been performed in only one type of endothelial cell. For this reason, it is not possible to extrapolate the conclusions drawn to all endothelial cells like the authors do.

This work advances our knowledge of endothelial cell biology and it will be of special interest for the vascular biology and development communities.

Reviewer 3

Evidence, reproducibility and clarity

Summary:

In this study, Essebier et al., investigated the impact of constitutive activation of cSrc on endothelial cell behavior during vascular sprouting and homeostasis. The authors generated various mutant versions of cSrc to enable the expression of wild type cSrc, constitutively active cSrc, or cSrc with a dysfunctional kinase domain in HUVEC. They used a range of in vitro methods, including traditional 2D culture techniques and cutting-edge approaches like microfabricated vessels for 3D cell culture. They showed that the constitutive activation of c- Src resulted in a vascular ballooning phenotype both in a 3D angiogenic sprouting assay and in microfabricated blood vessels subjected to shear stress. The expression of this mutant form of c-Src was associated with an increase of focal adhesion size and number and an increase of extracellular matrix degradation. The vascular ballooning phenotype induced by constitutive activation of c-Src was partially rescued by the pharmacological inhibition of the matrix metalloproteinase (MMPs).
Major:

- "This was further supported by our observation that there were no changes in proliferation in c-Src mutant cells grown in a 2D monolayer".
- Figure 1A appears to have increased number of cells in the c-Src-CA condition compared to the control condition. Could the authors quantify the number of cells/area as they did for their 3D vessel model? This would reinforce the idea that the ballooning phenotype they observe is not due to differences in proliferation.
- Would be strengthened with analysis of another proliferation marker, such as EdU label, which is incorporated only during S phase of the cell cycle. Comparing ki67 staining and EdU staining would provide more insights. Also, using their 3D vessel model for this analysis would increase its relevance.
- In Figure 1E', cells expressing the constitutively active form of cSrc appear to detach, giving the impression of cell death. Have the authors tested the viability/apoptosis of c-Src-CA cells, particularly in their 3D model?
- "Therefore, reduction of endothelial cell-cell contacts in c-Src-CA cells may be due to elevated VE-cadherin phosphorylation and subsequent internalisation", "As reduction in cell- cell junction integrity has been shown to increase migratory capacity and sprouting angiogenesis [38], our data suggest that a balanced control of both cell-matrix and cell-cell junctions is essential for mediating migration."
In general, it's not clear how constitutively active cSrc affects focal adhesions and cell-cell adhesion and how this is responsible for their ballooning phenotype. The role of the phosphorylation of the VE-Cadherin and cell-cell junctions in this process is not clear either.
- Further analysis of cell-cell junctions and focal adhesions (co-staining of phosphorylated paxillin and VE-Cadherin) and focal adhesions/fibronectin (like in figure 4C) in the context of cell migration (scratch wound assay) would provide important information to strengthen this notion of balanced control of both cell-matrix and cell-cell junctions.
- It is not clear how the constitutive activation of c-Src affects both cell-cell junction and focal adhesion morphology. Did the authors study signaling pathways downstream of c-Src such as

© 2024. Published by The Company of Biologists under the terms of the Creative Commons Attribution License (https://creativecommons.org/licenses/by/4.0/).

the PI3K-AKT pathway?
- "Taken together, these results reveal that proteases produced by c-Src-CA cells are locally secreted at FAs but are membrane bound."
The claim that proteases are membrane-bound is not convincingly demonstrated.
Could the authors assess whether the constitutive form of cSrc activates the expression of specific genes encoding MMPs by qPCR? Or is it more a matter of the effect of c-Src on the transport of MMPs by microtubules?

Minor:

- General comment: The authors have predominantly presented composite images with overlapping staining, making it challenging to differentiate between different labels. It would be beneficial if the authors could provide individual channel images along with a merge.
- The lab already showed in a previous study that mice lacking c-Src specifically in endothelial cells have reduced blood vessel sprouting, leading to the expectation that the constitutively active form of cSrc would increase sprout number in the sprouting assay. Could the authors explain why the constitutively active form of cSrc induces this vascular ballooning and not an increase in the number of sprouts?
- In Figure 1A, it would be beneficial to include images from orthogonal views. Indeed, in the c-Src-CA condition, it's not clear whether the vascular ballooning observed represents a cluster of cells or an empty space between the bead and the endothelial cells. (Supp movie 1 helps, but it would be useful to add orthogonal views to the figure)
- In Figure 1D, the method used to analyze sprout shape is not clear, especially for the c-Src- CA condition where the number of sprouts is close to 0. The figure legend indicates that this measurement corresponds to the shape of the sprouting area. Could the authors clarify and explain their quantification method?
- "however cells within the vessel still maintained come connections (Fig 1E')": The connections between cells are difficult to see in the images in Figure 1E'. Could the authors provide higher magnification images of the VE-cadherin staining to illustrate these connections between cells?
- "The reduction in migration correlated with an increase in FA size c-Src-CA expressing cells.": Could the authors give more explanation?
- Could the authors widen the cell trajectory trace in Supplementary Figure 3A?
- it is very difficult to distinguish fibronectin fibrils on the images shown in figure 4C. it would be beneficial to change the images.
- "Treatment of ECs with Marimastat in a fibrin bead sprouting assay resulted in a rescue of the ballooning morphology observed in the c-Src-CA cells"
Based on the images displayed in the figure and the associated quantifications, it still appears that c-Src-CA+Marimastat induces a vascular ballooning even if it is less pronounced than in the DMSO condition. Hence, it would be more accurate to describe the observed effect as a "partial rescue".
In the microfabricated 3D vessel, in the figure 7A, cell-cell junctions still appear altered by c-Src-CA after the treatment with Marimastat, compared to the c-Src-WT-Marimastat, it would be more appropriate to talk about "partial rescue".
- In Figure 6A, it seems that there is a decrease in the number of sprouts in the c-Src-DN condition compared to the control condition after the DMSO treatment, which is not observed in Figure 1, could the authors explain why?
- There is no statistical paragraph in the method section.

Referees cross-commenting

Agree that the comments of the reviews all seem reasonable. Since cultured EC do not retain very specialized characteristics, perhaps repeating experiments with many other ECs would not be helpful, but suggest some key experiments be performed with one other type of EC.

Significance

General assessment:

The authors generated different mutant forms of c-Src and used them in innovative 3D endothelial cell culture models. The vascular ballooning phenotype induced by constitutive

© 2024. Published by The Company of Biologists under the terms of the Creative Commons Attribution License (https://creativecommons.org/licenses/by/4.0/).

activation of c-Src is particularly interesting and impressive, especially as it can be reproduced in 2 different culture models. The model of cSrc inducing extracellular matrix degradation specifically at the level of focal adhesions is compelling, although it lacks rigorous support in the 3D model. Further analysis of signaling pathways downstream of c- Src would strengthen the work. The link and the necessity of a balance between cell adhesion and cell-cell junctions are mentioned and have started to be explored, particularly through the phosphorylation of Ve-Cadherin, and more in-depth analysis would strengthen this aspect of the work.

Advance:

This study provides new insight on the role of c-Src in vascular homeostasis and during sprouting angiogenesis and starts to explore cross-talk between EC cell junctions and focal adhesions. This study also provides new elements crucial for our understanding of vascular malformations and the implication of cell-adhesion to the extracellular matrix in this process. This study may lead to further investigations into the role of c-Src in tumor angiogenesis.

Audience:

Basic research / Specialized

Author response to reviewers' comments

Manuscript number: RC-2023-02176
Corresponding author(s): Lilian Schimmel, Emma Gordon (co-corresponding)

1. General Statements

We appreciate the reviewers' thoughtful feedback and thank them for their valuable suggestions to improve the manuscript. We have endeavored to respond to all their comments, with many of their concerns already incorporated in the manuscript. Validations for the additional experiments to be incorporated into the manuscript have been performed and show that all the plans outlined in Section 2 are highly feasible and will be added for the full revision. We believe that the incorporated and planned revisions contribute to a significant improvement on the original manuscript.

2. Description of the planned revisions

Reviewer 1
Major comments:
Point 3. p. 5. The authors do not describe any relationship to notch signaling. But notch signaling is the mechanism by which a sprout is selected. The CA phenotype shows no selection, and every sprout can continue migration. Did the authors check for any relationship between notch signaling c-Src activation? Does upregulation of C-Src downregulate notch?

In previous unpublished results examining the impact of the loss of endothelial c-Src on notch signaling, we observed no alteration in DLL4 expression in the sprouting retina on postnatal day 5 (*Revision Figure 1*). Furthermore, no change in tip cell number was observed in mice with a loss of endothelial c-Src, suggesting c-Src depletion does not impact notch activity (Schimmel et al., Development, 2020, Figure 1M). We have undertaken additional preliminary experiments performing immunostaining with a DLL4 antibody in migrating c-Src-CA cells (*Revision Figure 2*) to assess activation of notch signaling upon c-Src activation. We have not detected any significant changes in DLL4 intensity. We will continue these experiments for the full revision and will confirm the results via further analysis of notch activation by assessing DLL4 expression in the c-Src mutant cells using Western blot.

© 2024. Published by The Company of Biologists under the terms of the Creative Commons Attribution License (https://creativecommons.org/licenses/by/4.0/).

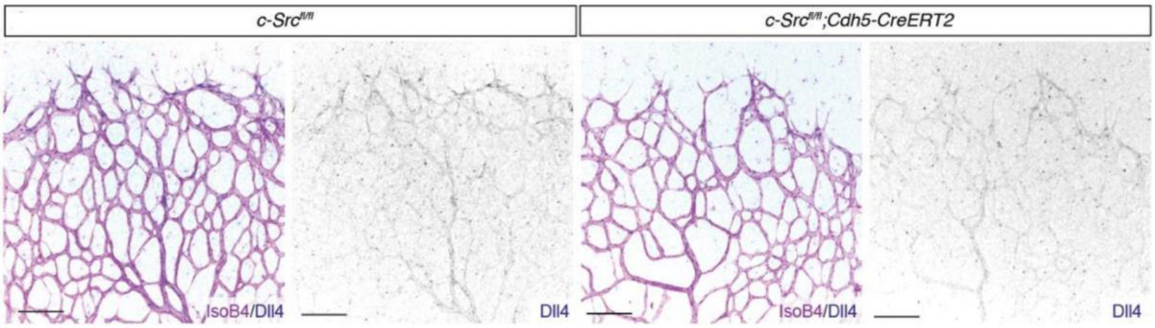

Revision Figure 1. c-Src deletion does not impact notch signalling*. (A) p5 mouse retinas with a loss of endothelial cell c-Src do not display altered DLL4 expression at the vascular front.*

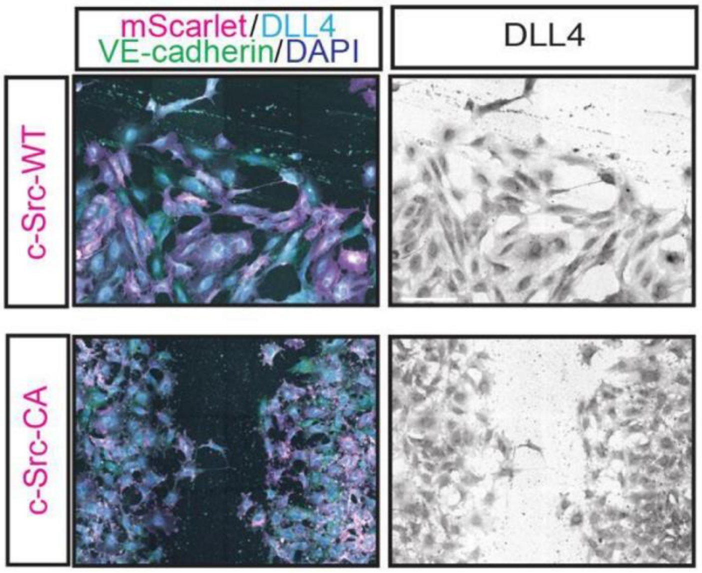

Revision Figure 2: DLL4 expression in migrating cells with constitutive c- Src activity*. (A) Representative images of HUVECs in a wound healing assay. Confluent monolayers were starved overnight in low serum media before inflicting a scratch and treating cells with 100 ng/mL VEGFA. After 16 h, cells were fixed and immunofluorescent staining was performed for delta-like 4 (DLL4; cyan), cell-cell junctions (VE- cadherin; green), and nuclei (DAPI; blue). No change in DLL4 intensity between c-Src-WT and c-Src-CA cells was observed.*

Reviewer 2

Major comments*:*

Point 1. The authors have only used one type of vein endothelial cells from one single donor but they conclude that is effect is general for all endothelial cells. Endothelial cells are very heterogeneous, not only depending on their function and localization, vein, artery or capillary, but also between different organs and in disease (PMID: 22315715, PMID: 28775214, PMID: 31944177, PMID: 33514719). The authors, should either repeat some of the key experiments in other type of endothelial cells, maybe arterial or microvasculature cells which are commercially available or at least state that the observations presented in this manuscript apply to HUVECs and discuss whether this would also apply for other cell types.

We agree it would be highly beneficial to assess whether c-Src-CA induces vascular expansion in other endothelial cell types. We have successfully transduced human arterial endothelial cells (HAEC) with empty vector and c-Src-CA lentivirus and are able to grow HAECs in 3D vessels (***Revision Figure 3***). This demonstrates that introducing the c-Src constructs into other endothelial cells and putting them in 3D assays is highly feasible. We have also used human microvascular endothelial cells (HMVEC) in 3D vessels in previous studies (Schimmel et al., Clin Trans Immunol, 2021). Therefore, we will perform experiments introducing the full set of c-Src

© 2024. Published by The Company of Biologists under the terms of the Creative Commons Attribution License (https://creativecommons.org/licenses/by/4.0/).

mutations in HAEC and/or HMVEC in 3D vessels for the revision to strengthen our findings.

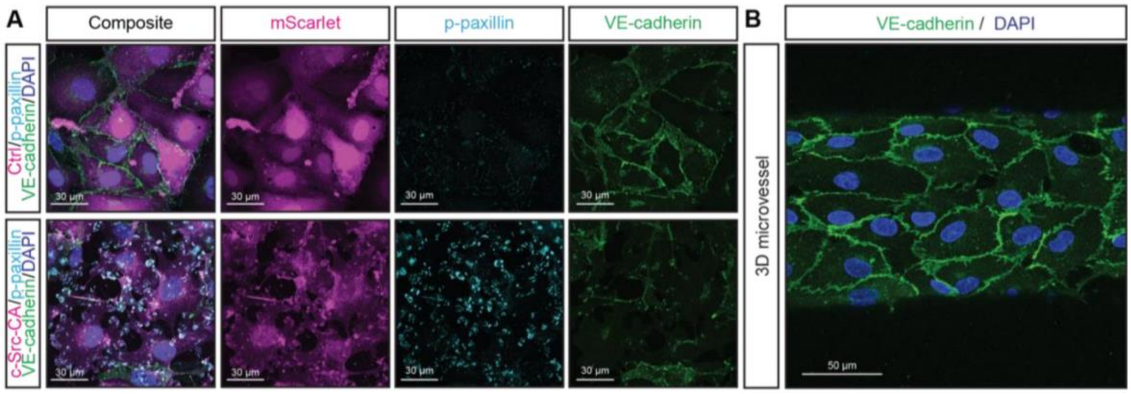

***Revision Figure 3. Arterial endothelial cells can be transduced with c-Src lentiviral plasmids and grown in 3D microvessels**. (A) Representative images of human arterial endothelial cells (HAECs) transduced with control or c-Src-CA lentiviral constructs grown in 2D monolayers and immunofluorescently stained for focal adhesions (p-paxillin Y118; cyan), cell-cell junctions (VE-cadherin; green), and nuclei (DAPI; blue). (B) Representative image of HAECs grown in 3D microfluidic vessels for 3 days before fixing and immunofluorescent staining for cell- cell junctions (VE-Cadherin; green) and nuclei (DAPI; blue).*

Reviewer 3
Major comments:
Point 1. "This was further supported by our observation that there were no changes in proliferation in c-Src mutant cells grown in a 2D monolayer".
Figure 1A appears to have increased number of cells in the c-Src-CA condition compared to the control condition. Could the authors quantify the number of cells/area as they did for their 3D vessel model? This would reinforce the idea that the ballooning phenotype they observe is not due to differences in proliferation.

We have started quantification on the number of cells per bead for the 3D bead sprouting experiments shown in Figure 1. Preliminary results show a trend towards increased numbers in c-Src-CA condition (***Revision Figure 4***). We will complete this quantification for 3 independent experiments and the results will be added for the full revision.

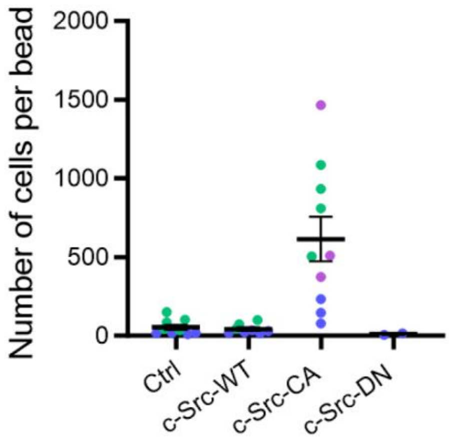

***Revision Figure 4. Number of cells per bead in 3D bead sprouting assay**. Number of cells was quantified based and DAPI positive cells within the vascular area of one bead (mean +/- SEM; n=1-3 independent experiments).*

Point 2. Would be strengthened with analysis of another proliferation marker, such as EdU label, which is incorporated only during S phase of the cell cycle. Comparing ki67 staining and EdU staining would provide more insig**hts. Also, using their 3D vessel model for this analysis would**

© 2024. Published by The Company of Biologists under the terms of the Creative Commons Attribution License (https://creativecommons.org/licenses/by/4.0/).

increase its relevance.

We agree that showing proliferation in a 3D setting would be highly beneficial. We tested proliferation marker Ki67 in 3D vessels to ensure this analysis will be possible (***Revision Figure 5***). We will perform full analysis of proliferation across c-Src mutations in 3D for the revision. We have also performed BrdU labelling in 2D to show that this analysis is feasible (***Revision Figure 5C***). Therefore, we will also perform full analysis of proliferation with BrdU across c-Src mutations for the revision.

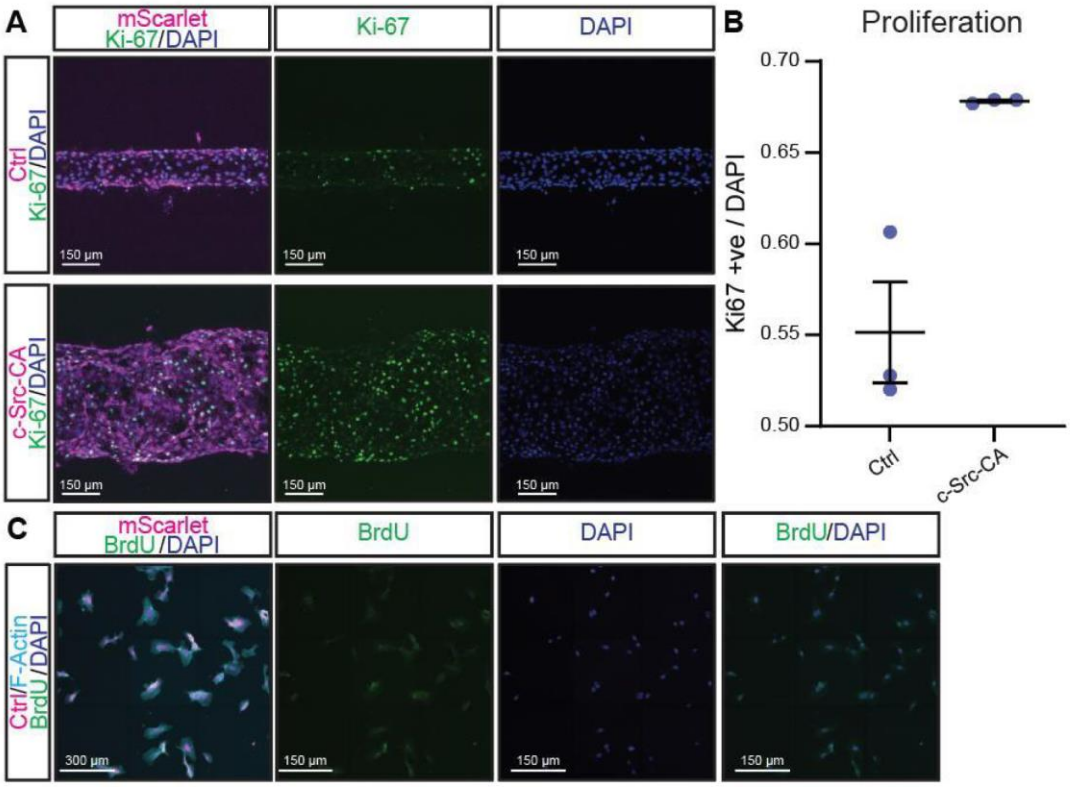

Revision Figure 5. Constitutive c-Src activity increases proliferation in 3D microvessels.
(A) Representative images of HUVECs seeded in PDMS microfluidic vessels containing 2.5 mg/mL collagen matrix for 3 days before fixing. Immunofluorescent staining was performed for Ki-67 (green) and nuclei (DAPI; blue). (B) Proliferation was quantified as the ratio of Ki-67 positive cells to total number of cells (mean +/- SEM; n=1 biological replicate). (C) Representative image of BrdU proliferation assay in 2D. HUVECs were treated with BrdU 1 h after seeding and grown for a further 24 h to perform immunofluorescent staining of BrdU (green), nuclei (DAPI; blue) and F-actin (Phalloidin; cyan).

Point 3. In Figure 1E', cells expressing the constitutively active form of cSrc appear to detach, giving the impression of cell death. Have the authors tested the viability/apoptosis of c-Src-CA cells, particularly in their 3D model?

We agree that showing cell death in our model, especially in a 3D setting, would be highly beneficial. We have tested cell death marker Cleaved Caspase 3 (CC-3) in 3D vessels to ensure this analysis is feasible (***Revision Figure 6***). We will perform full analysis of cell death across c- Src mutations in 3D for the revision.

© 2024. Published by The Company of Biologists under the terms of the Creative Commons Attribution License (https://creativecommons.org/licenses/by/4.0/).

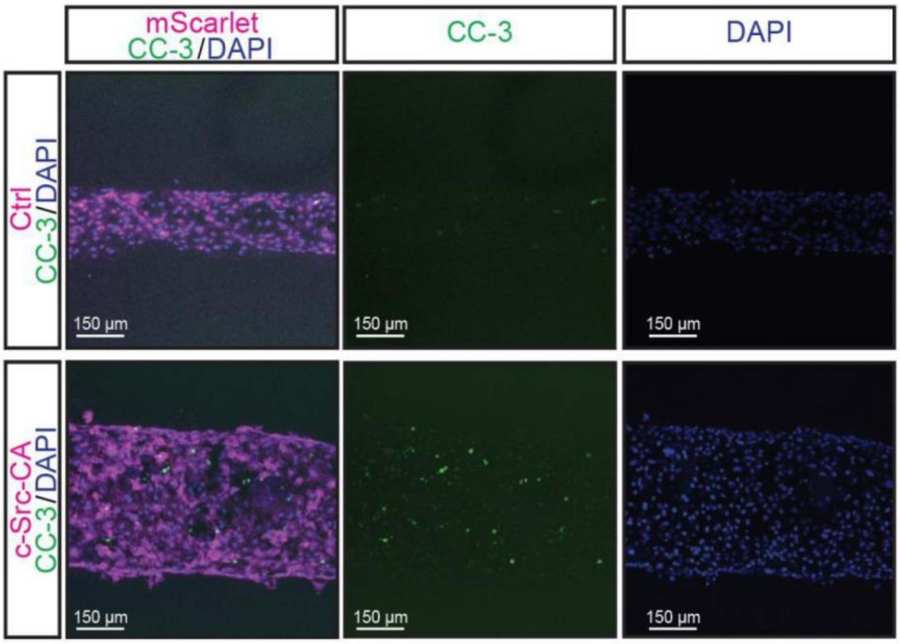

Revision Figure 6. Cleaved caspase 3 immunofluorescent staining n 3D microvessels. *Representative images of HUVECs grown in microfluidic vessels for 3 days before fixing. Staining was performed for cleaved-caspase 3 (CC-3; green) and nuclei (DAPI; blue).*

Point 4. "Therefore, reduction of endothelial cell-cell contacts in c-Src-CA cells may be due to elevated VE-cadherin phosphorylation and subsequent internalisation", "As reduction in cell-cell junction integrity has been shown to increase migratory capacity and sprouting angiogenesis [38], our data suggest that a balanced control of both cell-matrix and cell-cell junctions is essential for mediating migration." In general, it's not clear how constitutively active cSrc affects focal adhesions and cell-cell adhesion and how this is responsible for their ballooning phenotype. The role of the phosphorylation of the VE-Cadherin and cell-cell junctions in this process is not clear either. Further analysis of cell-cell junctions and focal adhesions (co-staining of phosphorylated paxillin and VE-Cadherin) and focal adhesions/fibronectin (like in figure 4C) in the context of cell migration (scratch wound assay) would provide important information to strengthen this notion of balanced control of both cell-matrix and cell-cell junctions.

We will perform experiments on migrating cells in 2D, co-staining for p-paxillin and VE-cadherin, and p-paxillin and Fibronectin (preliminary experiments performed, shown in *Revision Figure 7*), to address the role of balanced cell-matrix and cell-cell junction adhesion, and how they influence Fibronectin deposition in migration cells.

© 2024. Published by The Company of Biologists under the terms of the Creative Commons Attribution License (https://creativecommons.org/licenses/by/4.0/).

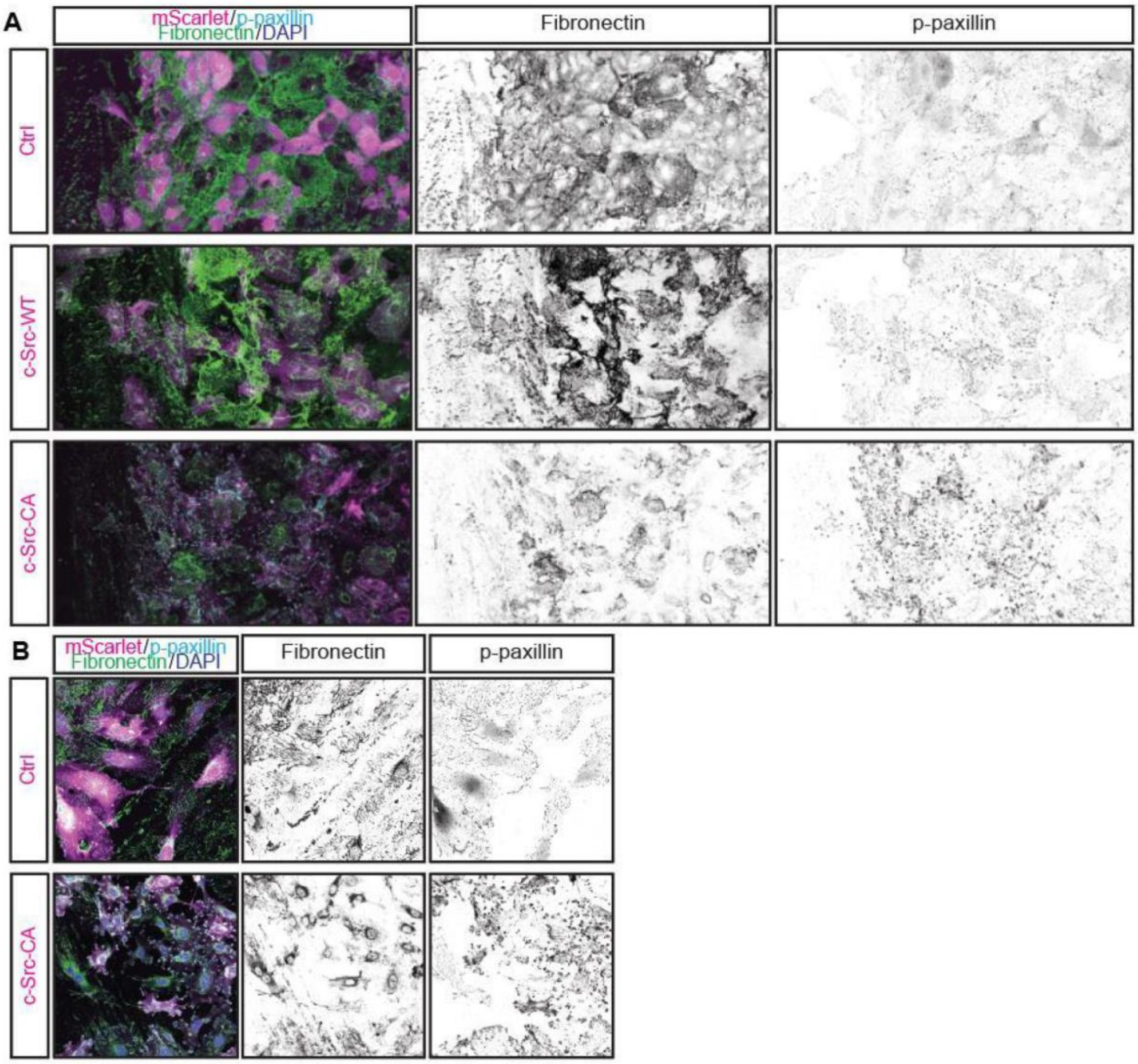

Revision Figure 7: Constitutive c-Src activity disrupts fibrillogenesis and increases focal adhesions in migrating cells. *(A-B) Representative tile scans of HUVECs in a wound healing assay. Confluent monolayers were starved overnight in low serum media before inflicting a scratch and treating cells with 100 ng/mL VEGFA. After 16 h cells were fixed and immunofluorescent staining was performed for focal adhesions (p-paxillin Y118; cyan), fibronectin (green) and nuclei (DAPI; blue) and imaged at low (A) and high (B) magnification.*

Point 6. "Taken together, these results reveal that proteases produced by c-Src-CA cells are locally secreted at FAs but are membrane bound." The claim that proteases are membrane-bound is not convincingly demonstrated. Could the authors assess whether the constitutive form of cSrc activates the expression of specific genes encoding MMPs by qPCR? Or is it more a matter of the effect of c-Src on the transport of MMPs by microtubules?

We would like to clarify the content of Figure 5, which presents two distinct sets of experiments supporting the assertion that the proteases under investigation are membrane-bound. Firstly, the transfer of conditioned medium from c-Src mutant cells demonstrated no degradation of fibronectin fibrils. Secondly, in the bead sprouting assay, a mixed culture of untransduced and c-Src-CA expressing cells was utilised. The results revealed that only c-Src-CA cells formed balloons, while untransduced cells sprouted normally right next to or sometimes even through a balloon.

Recognising the need for a more in-depth understanding, we acknowledge the importance of analysing specific MMP gene expression. To this end, we have ordered qPCR primers for distinct MMPs, namely MMP2, MMP7, MMP9, and MT1-MMP. These forthcoming experiments are not only

© 2024. Published by The Company of Biologists under the terms of the Creative Commons Attribution License (https://creativecommons.org/licenses/by/4.0/).

highly feasible but will also contribute valuable insights. The results of this gene expression analysis will be incorporated into the revision, shedding light on whether constitutively active c-Src induces MMP gene expression or influences MMP transport.

Minor comments:

Point 2. The lab already showed in a previous study that mice lacking c-Src specifically in endothelial cells have reduced blood vessel sprouting, leading to the expectation that the constitutively active form of cSrc would increase sprout number in the sprouting assay. Could the authors explain why the constitutively active form of cSrc induces this vascular ballooning and not an increase in the number of sprouts?

In line with analysis to be performed on notch activity and DLL4 expression (Reviewer 1 point 3, ***Revision Figures 1,2***), we will provide additional discussion on the role of notch signalling and tip cell identity with the full revision.

3. Description of the revisions that have already been incorporated in the transferred manuscript

Reviewer 1

Major comments:

Point 1. p5. Fig 1: The sentence that the dominant negative completely abrogated 'this' phenotype implies that the dominant negative was put into the same cells as the constitutively active mutation. 'Abrogated' means it stops the phenotype, and the phenotype in the sentence prior was constitutively active. It is more accurate to say that the dominant negative was not distinguishable from wild type, which is what the statistics show. No double transfection (DN-CA) was performed.

We have changed the wording in the manuscript accordingly to 'The c-Src-DN mutation showed no phenotype distinguishable from Ctrl (Fig 1A-D).' on page 5.

Point 2. p.5. Fig 1: the phenotype of the CA cells is fascinating. They expand far beyond their normal territory, but they are held together in a lacy bubble. To me, this looks like a different phenotype from the ballooning that might occur in an arteriovenous malformation in vivo, as in vivo malformations are continuously covered by cells. I understand why the authors might use the term ballooning but given that the cells expand without continuously touching each other, I do not think this is the correct term. Would blebbing, or radial migration in a lace-like discontinuous pattern describe it better?

We have changed the phrasing from 'ballooning morphology' to 'radial migration in a lace-like discontinuous pattern' on page 5. For brevity, this has been referred to as 'ballooning' for the remainder of the manuscript, as noted on page 5.

Point 4. The statistical methods are not described in the methods (GraphPad?). These need to be added. Are only significant comparisons plotted? In Fig 6 and 7 only pairwise statistics are shown. If all significant comparisons are plotted, then this means that the comparison between the rescued CA and the treated or untreated control is not significant. This can be thought of as a partial rescue towards a wild type, but it is definitely not a full rescue. None of the statistical comparisons in Figure 6 or 7 show significant comparisons to wildtype. This needs more discussion.

We have now added additional clarification on statistical methods. Details on the statistical tests for each figure are mentioned in the figure legends. A general section on the statistical methods is now added to the methods section on page 18.

Only significant comparisons are displayed in the graphs, but as mentioned by reviewer 2 (minor point 2), we have added additional information for transparency. Each of the different comparisons that were made, and their precise p value, have been compiled a table which has been added as Supplementary Table 1 to the manuscript.

In Figures 6 and 7, we exclusively plotted pairwise comparisons to assess the impact of Marimastat treatment. As outlined in Supplementary Table 1, there is still a statistical significance when comparing Marimastat-treated c-Src-CA with either Marimastat-treated Ctrl or Marimastat- treated

© 2024. Published by The Company of Biologists under the terms of the Creative Commons Attribution License (https://creativecommons.org/licenses/by/4.0/).

c-Src-WT. This suggests a partial rescue. For clarity, we kept only pairwise comparisons in the graphs, but discussed the partial rescue due to remaining significant difference between Marimastat-treated c-Src-CA and Ctrl or c-Src-WT cells in the results, referring to Supplementary Table 1 for p values. An important sidenote: c-Src-CA treated cells cannot exhibit complete rescue since they are initially seeded without Marimastat, and have already initiated ballooning by the time treatment commences.

Point 5. Mmp activity is inferred, but not measured. This is a limitaion as the assumption is that marimostat acting through the expected pathway.

Marimastat is one of the most commonly used broad spectrum MMP inhibitors, with potent activity against major MMPs, including MMP1, MMP3, MMP2, MMP9, MMP7 and MMP14. This is outlined in the existing reference (Rasmussen and McCann, 1997). We have adjusted phrasing to clarify the potency of Marimastat and have emphasised this is an MMP targeting drug which has been widely utilised in oncology clinical trials (page 8).

Minor comments:
Point 1. Fig 5D. The presentation of the data in this graph is difficult to understand. It is trying to show the proportion of mScarlet in sprouts or balloons a percentage of all the scarlet cells. It would be better to have all cells represented in one bar, distributed between sprout and balloon in that one bar. i.e., for the control and dominant negative, the bars would be all black and then for the CA it would be all white. The zero data points are confusing. A proportions graph should be investigated here.

We have changed the graph in Figure 5D, which now represents the % of the outgrowth area, sprouts for Ctrl, c-Src-WT and c-Src-DN and balloon for c-Src-CA, that are mScarlet positive. Resulting in all black bars for Ctrl, c-Src-WT and c-Src-DN and all white bar for c-Src-CA, as the reviewer predicted.

Point 2. The methods for vessel coverage for quantification in figs 1 and 7 are missing.

We have added details of how quantification of vessel coverage in Figure 1 and 7 was performed to the methods section on page 17/18 as follow: 'Microfluidic vessel coverage was measured by tracing any holes in the vessel wall (inverse of cell area marked by phalloidin) and dividing this by the total cell area per image.'

Reviewer 2
Minor comments:
Point 1. Although the methods are well written and can be understood. To improve transparency, the authors should reduce the referring to other papers to describe the methods they perform and at least some kind of brief description should be included.

We have added a brief description of the methods that included references to other papers; lentiviral transduction and microfluidic devices. More details about the lentivirus transduction were added on page 15 and a short description about the fabrication of the microfluidic devices was added on page 15/16.

Point 2. The authors should report the real p value for their tests. Also, when the test is not significant.

To provide more transparency about all of the different comparisons that were made and their precise p value, we have compiled a table listing all the p values and which is added as Supplementary Table 1 to the manuscript.

Reviewer 3
Minor comments:
Point 3. In Figure 1A, it would be beneficial to include images from orthogonal views. Indeed, in the c-Src-CA condition, it's not clear whether the vascular ballooning observed represents a cluster of cells or an empty space between the bead and the endothelial cells. (Supp movie 1 helps, but it would be useful to add orthogonal views to the figure)

© 2024. Published by The Company of Biologists under the terms of the Creative Commons Attribution License (https://creativecommons.org/licenses/by/4.0/).

For clarity, we have added single Z plane image for cross sectional views of the bead sprouts in Figure 1A to show that the c-Src-CA cells have an empty space inside the balloon, rather than being a big cluster of cells.

Point 4. In Figure 1D, the method used to analyze sprout shape is not clear, especially for the c-Src-CA condition where the number of sprouts is close to 0. The figure legend indicates that this measurement corresponds to the shape of the sprouting area. Could the authors clarify and explain their quantification method?

The shape of the sprouting area refers to the circularity index of the vascular area, measured by tracing the perimeter of the cell area in a minimum Z-projection of brightfield images and subtracting the area of the bead. For better clarity, we have adjusted the title of Figure 1D and Figure 6D to 'Vascular area shape' and added details of the quantification method in the methods section on page 17.

Point 5. "however cells within the vessel still maintained some connections (Fig 1E')": The connections between cells are difficult to see in the images in Figure 1E'. Could the authors provide higher magnification images of the VE-cadherin staining to illustrate these connections between cells?

For improved clarity, we have added high magnification images of the VE-cadherin channel only in black and white (Figure 1E'') and indicated some of the maintained cell-cell connections in the c-Src-CA cells with black arrowheads.

Point 6. "The reduction in migration correlated with an increase in FA size c-Src-CA expressing cells.": Could the authors give more explanation?

We have adjusted phrasing to provide additional information (page 6/7) as follows:
'The reduction in migration velocity in c-Src-CA cells coincides with an increase in FA size, number and density (Fig 2A-D). This suggests that the reduction of migration velocity is due to increased cellular adhesion via FAs.'

Point 7. Could the authors widen the cell trajectory trace in Supplementary Figure 3A?

We have adjusted the trajectory traces in Supplementary Figure 3A with wider lines for improved visibility.

Point 8. it is very difficult to distinguish fibronectin fibrils on the images shown in figure 4C. it would be beneficial to change the images.

We have enlarged the zoomed areas for better visibility of the focal adhesions and fibronectin degradation underneath those areas in the c-Src-CA cells. Additionally, arrows are added to indicate fibronectin fibrils.

Point 9. "Treatment of ECs with Marimastat in a fibrin bead sprouting assay resulted in a rescue of the ballooning morphology observed in the c-Src-CA cells" Based on the images displayed in the figure and the associated quantifications, it still appears that c-Src-CA+Marimastat induces a vascular ballooning even if it is less pronounced than in the DMSO condition. Hence, it would be more accurate to describe the observed effect as a "partial rescue". In the microfabricated 3D vessel, in the figure 7A, cell-cell junctions still appear altered by c-Src-CA after the treatment with Marimastat, compared to the c-Src-WT-Marimastat, it would be more appropriate to talk about "partial rescue".

We have changed 'rescue' to 'partial rescue' when referring to results in Figure 6 and 7 (page 8).

Point 10. In Figure 6A, it seems that there is a decrease in the number of sprouts in the c-Src- DN condition compared to the control condition after the DMSO treatment, which is not observed in Figure 1, could the authors explain why?

© 2024. Published by The Company of Biologists under the terms of the Creative Commons Attribution License (https://creativecommons.org/licenses/by/4.0/).

In Figure 1C, the number of sprouts is also reduced in the c-Src-DN condition compared to c-Src-WT, but this is not significant when compared to control (see Supplementary Table 1 for p values of all comparisons). However, it is true that the number of sprouts in the c-Src-DN condition is significantly reduced compared to both control and c-Src-WT upon DMSO treatment (Fig 6C). Reduction of sprouts in c-Src-DN cells was expected due to the dysfunctional kinase domain, as mentioned on page 5 and shown in reference 30 (Shvartsman, D.E., et al., J Cell Biol, 2007. 178(4): p. 675-86.). Why DMSO treatment seems to enhance the effects of dominant negative c-Src expression on sprouting behaviour remains unclear. However, DMSO has adverse effects on sprouting shown by reduction of sprouts in both control and c-Src-WT cells (comparing untreated condition in Fig 1C with DMSO treated condition in Fig 6C). We believe that DMSO treatment is an extra challenge for cells on top of c-Src-DN expression, which therefore display reduced sprouting compared to control and c-Src-WT.

Point 11. There is no statistical paragraph in the method section.

As pointed out by reviewer 1 and 2, we have now added a general section on the statistical methods to the method section on page 18. Additional details on the tests used for each specific graph can be found in the figure legends and Supplementary Table 1.

4. Description of analyses that authors prefer not to carry out

Please include a point-by-point response explaining why some of the requested data or additional analyses might not be necessary or cannot be provided within the scope of a revision. This can be due to time or resource limitations or in case of disagreement about the necessity of such additional data given the scope of the study. Please leave empty if not applicable.

Reviewer 3

Major comments:

Point 5. It is not clear how the constitutive activation of c-Src affects both cell-cell junction and focal adhesion morphology. Did the authors study signaling pathways downstream of c-Src such as the PI3K-AKT pathway?

c-Src is well known to regulate a multitude of signalling pathways, which was definitively shown in analysis by Ferrando et al. using phosphoproteomics (Ferrando, I.M., et al., Mol Cell Proteomics, 2012. 11(8): p. 355-69.) In this manuscript, our primary emphasis is on elucidating the role of c-Src in governing cell-matrix adhesions and the degradation of the extracellular matrix. We delve into the nuanced connection between focal adhesions (FAs) and VE-cadherin through the actin framework in the discussion (see page 10). Additionally, we highlight that beyond its recognised direct targets in FAs and adherens junctions (AJs), c-Src exerts regulatory influence on these structures through its effects on the actin cytoskeleton.

The PI3K/AKT pathway is implicated in the progression of vascular malformations in Hereditary Hemorrhagic Telangiectasia (HHT), where patients exhibit rapid vasculature expansion akin to the observed effects upon introducing the c-Src-CA mutation. In HHT, PTEN inhibition triggers heightened activity of VEGFA/VEGFR2 and subsequent AKT kinase activation. Although we have conducted preliminary analysis revealing elevated phospho-AKT (*Revision Figure 8*), we contend that an in-depth examination of each signaling pathway perturbed downstream of c-Src- CA is beyond the current scope of this manuscript. Our future studies will specifically address this, providing a meticulous exploration of c-Src activity in HHT and its intricate interaction with the AKT pathway.

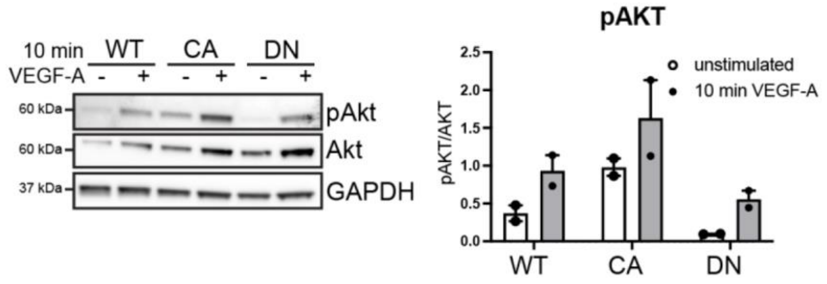

© 2024. Published by The Company of Biologists under the terms of the Creative Commons Attribution License (https://creativecommons.org/licenses/by/4.0/).

Revision Figure 8. c-Src activity can alter AKT signalling. *(A) HUVEC were transduced with mutant virus and stimulated with VEGF-A. Preliminary results suggests that c-Src-CA cells display increased pAKT.*
Minor comments:
Point 1: General comment: The authors have predominantly presented composite images with overlapping staining, making it challenging to differentiate between different labels. It would be beneficial if the authors could provide individual channel images along with a merge.

Given the large numbers of multi-channel composite images, we believe it is not feasible to show each individual channel of every merged image in the manuscript. We have included individual channel images where we believe is appropriate. For example, p-paxillin Y118 (Figure 2), Fibronectin (Figure 4). We are happy to provide individual channel images for any image, where specifically requested, such as in Figure 1E'' where VE-cadherin channel was added.

Point-by-point response to reviewers.

Manuscript number: RC-2023-02176
Corresponding authors: Lilian Schimmel, Emma Gordon

General statements

We appreciate the reviewers thoughtful feedback and thank them for their valuable suggestions to improve the manuscript. We have endeavoured to respond to all their comments, with the majority of the suggestions incorporated in the manuscript. Specific comments are addressed below.

Reviewer comments

Reviewer #1
Major comments:

Point 1. p5. Fig 1: The sentence that the dominant negative completely abrogated 'this' phenotype implies that the dominant negative was put into the same cells as the constitutively active mutation. 'Abrogated' means it stops the phenotype, and the phenotype in the sentence prior was constitutively active. It is more accurate to say that the dominant negative was not distinguishable from wild type, which is what the statistics show. No double transfection (DN-CA) was performed.

We have changed the wording in the manuscript accordingly to 'The c-Src-DN mutation showed no phenotype distinguishable from Ctrl (Fig 1A-E).' on page 5.

Point 2. p.5. Fig 1: the phenotype of the CA cells is fascinating. They expand far beyond their normal territory, but they are held together in a lacy bubble. To me, this looks like a different phenotype from the ballooning that might occur in an arteriovenous malformation in vivo, as in vivo malformations are continuously covered by cells. I understand why the authors might use the term ballooning but given that the cells expand without continuously touching each other, I do not think this is the correct term. Would blebbing, or radial migration in a lace-like discontinuous pattern describe it better?

We have changed the phrasing from 'ballooning morphology' to 'rapid radial migration in a lace-like discontinuous pattern' on page 5. For brevity, this has been referred to as 'ballooning' for the remainder of the manuscript, as noted on page 5.

Point 3. p. 5. The authors do not describe any relationship to notch signaling. But notch signaling is the mechanism by which a sprout is selected. The CA phenotype shows no selection, and every sprout can continue migration. Did the authors check for any relationship between notch signaling c-Src activation?

© 2024. Published by The Company of Biologists under the terms of the Creative Commons Attribution License (https://creativecommons.org/licenses/by/4.0/).

In unpublished results examining the impact of the loss of endothelial c-Src on Notch signaling, we did not observed any change in DLL4 expression in the sprouting retina on postnatal day 5 (***Revision Figure 1A***). Furthermore, no change in tip cell number was observed in mice with a loss of endothelial c-Src, suggesting c-Src depletion does not impact Notch activity (Schimmel et al., Development, 2020, Figure 1M). We have undertaken multiple additional experiments to address the relationship between Notch signalling and c-Src activation. Immunofluorescent staining on migrating control and c-Src-CA cells in a scratch assay did not reveal DLL4 enrichment at the sprouting front (***Revision Figure 1B***). We assessed activation of the Notch pathway after 10 minutes of VEGF stimulation in control and c-Src-mutant cells by qPCR for downstream effector proteins RBPJ, HEY1 and HEY2, which did not reveal any specific trends (***Revision Figure 1C***). Finally, we assessed DLL4 expression by Western blot of c-Src mutant cells, where we did not detect any significant changes. The data has been added to Supp Fig 1F-G and was added to the results section on page 5.

The effects of c-Src activation on Notch signalling is interesting and may provide an additional mechanism as to why the c-Src-CA cells induce vascular ballooning and not an increase in the number of sprouts. However, in our wide range of experimental investigation we have not detected changes in Notch activation. Therefore, it is unlikely the phenotype we observe in c-Src-CA cells is due to alterations in Notch signalling.

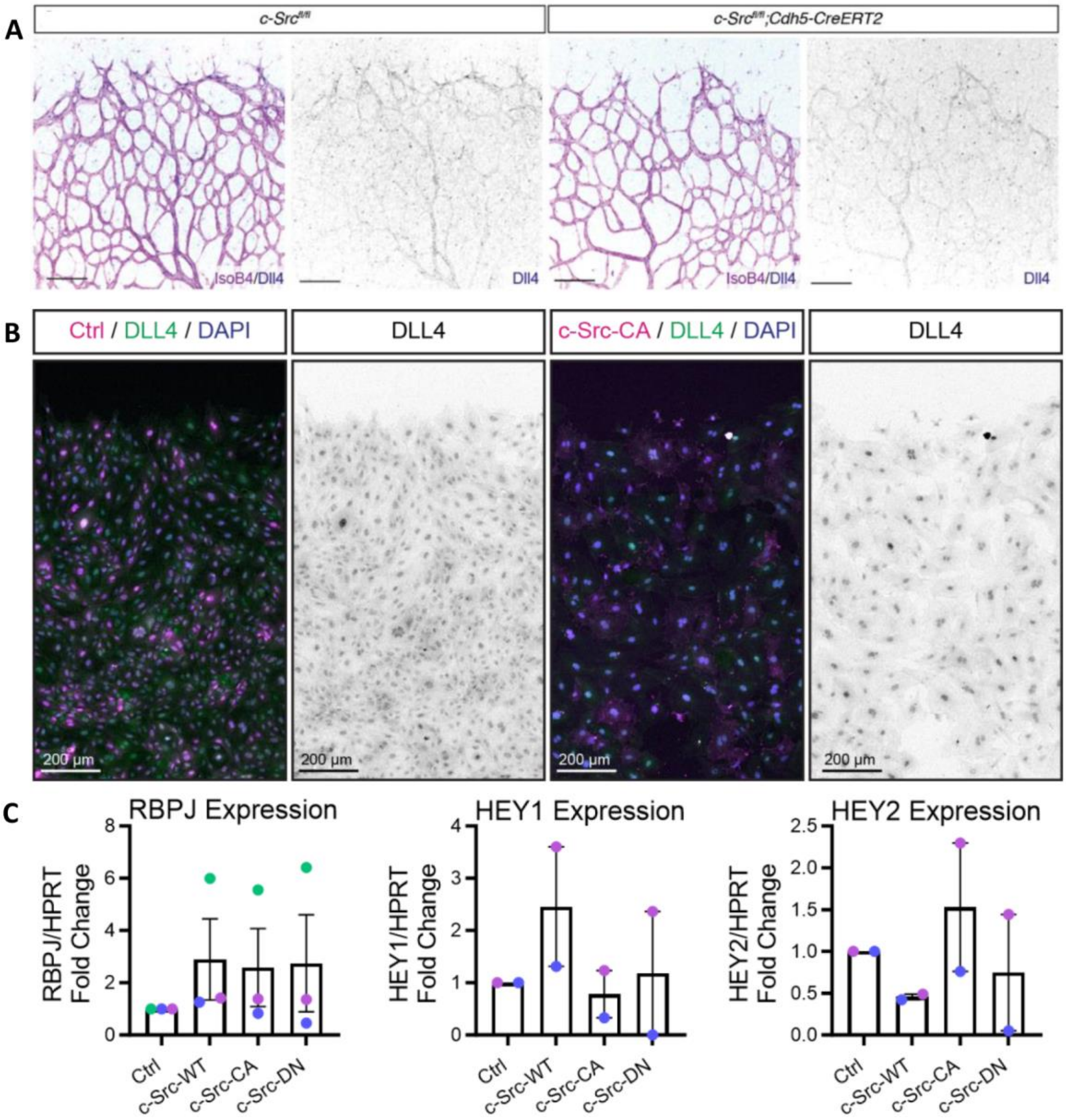

© 2024. Published by The Company of Biologists under the terms of the Creative Commons Attribution License (https://creativecommons.org/licenses/by/4.0/).

Revision Figure 1. c-Src does not regulate Notch signalling. (A) *p5 mouse retinas with a loss of endothelial cell c-Src (c-Src$^{fl/fl}$;Cdh5-CreERT2) do not display altered DLL4 expression at the vascular front compared to control littermates (c-Src$^{fl/fl}$).* ***(B)*** *HUVEC were transduced with mScarlet-tagged c-Src mutants (magenta) were scratched and stimulated with VEGF-A for 6 hr before fixation. Immunofluorescent staining was performed for DLL4 (green and grey individual channel) and nuclei (DAPI; blue).* ***(C)*** *HUVEC were transduced with mScarlet-tagged c-Src mutants and stimulated with VEGF-A for 10 min before harvesting for qPCR. No significant changes in transcription of Notch pathway-related genes RBPJ, HEY1, and HEY2 was observed. n = 2-3 independent experiments.*

Point 4. The statistical methods are not described in the methods (GraphPad?). These need to be added. Are only significant comparisons plotted? In Fig 6 and 7 only pairwise statistics are shown. If all significant comparisons are plotted, then this means that the comparison between the rescued CA and the treated or untreated control is not significant. This can be thought of as a partial rescue towards a wild type, but it is definitely not a full rescue. None of the statistical comparisons in Figure 6 or 7 show significant comparisons to wildtype. This needs more discussion.

We have now added additional clarification on statistical methods. Details on the statistical tests for each figure are mentioned in the figure legends. A general section on the statistical methods has been added to the methods section on page 18.

Only significant comparisons are displayed in the graphs, but as mentioned by reviewer 2 (minor point 2), we have added additional information for transparency. Each of the different comparisons that were made, and their precise p value, have been compiled a table which has been added as Supplementary Table 1 to the manuscript.
In Figures 6 and 7, we focused exclusively on pairwise comparisons to evaluate the effect of Marimastat treatment. As detailed in Supplementary Table 1, statistical significance is still observed when comparing cells treated with Marimastat in the c-Src-CA group to those in the Ctrl/c-Src-WT group, suggesting a partial rescue. To maintain clarity in the presentation, we included only these pairwise comparisons in the figures. However, we discuss the partial rescue in the results section, noting the significant differences between the Marimastat-treated c-Src-CA and Ctrl/c-Src-WT cells, and refer to Supplementary Table 1 for the specific p-values. It is also important to note that complete rescue is not possible in c-Src-CA treated cells as they are initially seeded without Marimastat for 24h in bead sprouting assay and 4 h in 3D microvessels and begin to show ballooning before treatment is applied.

Point 5. Mmp activity is inferred, but not measured. This is a limitation as the assumption is that marimastat acting through the expected pathway.

Marimastat is one of the most commonly used broad spectrum MMP inhibitors, with potent activity against major MMPs, including MMP1, MMP3, MMP2, MMP9, MMP7 and MMP14. This is outlined in the existing reference (Rasmussen and McCann, 1997). We have adjusted phrasing to clarify the potency of Marimastat and have emphasised this is an MMP targeting drug which has been widely utilised in oncology clinical trials (page 8).

Minor concerns:

Point 1. Fig 5D. The presentation of the data in this graph is difficult to understand. It is trying to show the proportion of mScarlet in sprouts or balloons a percentage of all the scarlet cells. It would be better to have all cells represented in one bar, distributed between sprout and balloon in that one bar. i.e., for the control and dominant negative, the bars would be all black and then for the CA it would be all white. The zero data points are confusing. A proportions graph should be investigated here.

We have changed the graph in Figure 5D, which now represents the % of the outgrowth area, sprouts for Ctrl, c-Src-WT and c-Src-DN and balloon for c-Src-CA, that are mScarlet positive. This results in all black bars for Ctrl, c-Src-WT and c-Src-DN and an all white bar for c-Src-CA, as the reviewer predicted.

Point 2. The methods for vessel coverage for quantification in figs 1 and 7 are missing.

© 2024. Published by The Company of Biologists under the terms of the Creative Commons Attribution License (https://creativecommons.org/licenses/by/4.0/).

We have added details of how quantification of vessel coverage in Figure 1 and 7 was performed to the methods section on page 18 as follow: ‘Microfluidic vessel coverage was measured by tracing any holes in the vessel wall (inverse of cell area marked by phalloidin) and dividing this by the total cell area per image.’

Reviewer #2

Major comments:

Point 1. The authors have only used one type of vein endothelial cells from one single donor but they conclude that is effect is general for all endothelial cells. Endothelial cells are very heterogeneous, not only depending on their function and localization, vein, artery or capillary, but also between different organs and in disease (PMID: 22315715, PMID: 28775214, PMID: 31944177, PMID: 33514719).

The authors, should either repeat some of the key experiments in other type of endothelial cells, maybe arterial or microvasculature cells which are commercially available or at least state that the observations presented in this manuscript apply to HUVECs and discuss whether this would also apply for other cell types.

We agree it would be highly beneficial to assess whether c-Src-CA induces vascular expansion in other endothelial cell types. We have successfully replicated the ballooning phenotype induced by c-Src-CA mutant in human aortic endothelial cells (HAEC) using 3D microvessels. Results reveal that constitutive activation of c-Src in arterial ECs induces the same ballooning phenotype as that observed in venous ECs (Supp Fig 1H-I). These results are discussed on page 6 of the manuscript. We have also shown that MMP inhibition, via broad spectrum inhibitor marimastat, partially rescues the ballooning of c-Src-CA HAECs in 3D microvessels. This data is presented in Supp Fig 7 and discussed on page 9.

Minor comments:

Point 1. Although the methods are well written and can be understood. To improve transparency, the authors should reduce the referring to other papers to describe the methods they perform and at least some kind of brief description should be included.

We have added a brief description of the methods used for lentiviral transduction and generation of microfluidic devices. Details on lentivirus transduction has been added on page 15 and a short description of the fabrication of the microfluidic devices has been added on page 16.

Point 2. The authors should report the real p value for their tests. Also, when the test is not significant.

To provide additional transparency about all of the different comparisons that were made and their precise p value, we have compiled a table listing all the p values (Supplementary Table 1).

Reviewer #3

Major comments:

Point 1. "This was further supported by our observation that there were no changes in proliferation in c- Src mutant cells grown in a 2D monolayer". Figure 1A appears to have increased number of cells in the c- Src-CA condition compared to the control condition. Could the authors quantify the number of cells/area as they did for their 3D vessel model? This would reinforce the idea that the ballooning phenotype they observe is not due to differences in proliferation.

We have quantified the number of cells per bead in the 3D bead sprouting experiments, as shown in Figure 1. The results, added to Figure 1E, indicate a higher number of ECs in beads containing c-Src-CA. However, our data from the 3D microvessel experiments (Fig 1F, H) did not show a significant increase in cell number. It's important to note that the bead sprouting assay involves a culture period of 7 days, whereas the microvessels are cultured for only 3 days. This longer duration in the bead sprouting assay might explain the increased cell numbers observed with c-Src-CA beads compared to the microvessels.

To investigate the possible effects of c-Src-CA mutant on cell proliferation, we have performed additional proliferation analysis as requested by reviewer 3 (major points 2 and 3).

Point 2. Would be strengthened with analysis of another proliferation marker, such as EdU label, which is incorporated only during S phase of the cell cycle. Comparing ki67 staining and EdU staining would provide more insights. Also, using their 3D vessel model for this analysis would increase its relevance.

© 2024. Published by The Company of Biologists under the terms of the Creative Commons Attribution License (https://creativecommons.org/licenses/by/4.0/).

We have performed EdU staining in 2D to compare with our existing Ki-67 staining, which showed a significant decrease in proliferation of c-Src mutant cells (Supp Fig 1L-M). However, we agree that showing proliferation in a 3D setting would be highly beneficial. To this end, we have performed proliferation analysis of 3D microvessels using both Ki-67 and EdU as proliferation markers. Ki-67 and EdU analysis revealed there are no significant changes in proliferation of the c-Src mutants compared to Control (Supp Fig 2A-C). Discussion of the EdU and Ki-67 results has been added to the results section on page 6.

Point 3. In Figure 1E', cells expressing the constitutively active form of cSrc appear to detach, giving the impression of cell death. Have the authors tested the viability/apoptosis of c-Src-CA cells, particularly in their 3D model?

We agree that showing cell death in our model, especially in a 3D setting, would be highly beneficial. Therefore, we have analysed cell death in 3D vessels using Cleaved Caspase 3 (CC-3). The results revealed no significant changes in cell death of c-Src mutants compared to control when grown in a 3D microvessel for 3 days (Supp Fig 2I-J). These results have been discussed on page 6 of the manuscript.

Point 4. "Therefore, reduction of endothelial cell-cell contacts in c-Src-CA cells may be due to elevated VE-cadherin phosphorylation and subsequent internalisation", "As reduction in cell-cell junction integrity has been shown to increase migratory capacity and sprouting angiogenesis [38], our data suggest that a balanced control of both cell-matrix and cell-cell junctions is essential for mediating migration."
In general, it's not clear how constitutively active cSrc affects focal adhesions and cell-cell adhesion and how this is responsible for their ballooning phenotype. The role of the phosphorylation of the VE- Cadherin and cell-cell junctions in this process is not clear either. Further analysis of cell-cell junctions and focal adhesions (co-staining of phosphorylated paxillin and VE-Cadherin) and focal adhesions/fibronectin (like in figure 4C) in the context of cell migration (scratch wound assay) would provide important information to strengthen this notion of balanced control of both cell-matrix and cell- cell junctions.

We have performed experiments on actively migrating ECs in 2D, co-stained for p-paxillin and fibronectin, to address the role of balanced cell-matrix adhesion in the context of migration. A traditional scratch assay would damage the fibronectin coating, therefore we used ibidi cell culture inserts in FN coated glass bottom dishes. Upon removal of the insert, cells were allowed to migrate for 3 hours before fixation and immunofluorescent staining. We performed analysis as per Figure 4C-D, which revealed that there is localised fibronectin degradation at the focal adhesion sites at the leading edge of migrating ECs. This proves that also in the context of cell migration, balanced cell-matrix adhesion is established via local ECM degradation. The new data is added to Supp Figure 5C-D and results are discussed on page 8.

Point 5. It is not clear how the constitutive activation of c-Src affects both cell-cell junction and focal adhesion morphology. Did the authors study signaling pathways downstream of c-Src such as the PI3K- AKT pathway?

c-Src is well known to regulate a multitude of signalling pathways, which was definitively shown in analysis by Ferrando et al. using phosphoproteomics (Ferrando, I.M., et al., Mol Cell Proteomics, 2012. 11(8): p. 355-69.) In this manuscript, our primary emphasis is on elucidating the role of c-Src in governing cell-matrix adhesions and the degradation of the extracellular matrix. We delve into the nuanced connection between focal adhesions (FAs) and VE-cadherin through the actin framework in the discussion (see page 10). Additionally, we highlight that beyond its recognised direct targets in FAs and adherens junctions (AJs), c-Src exerts regulatory influence on these structures through its effects on the actin cytoskeleton.

The PI3K/AKT pathway is implicated in the progression of vascular malformations in Hereditary Hemorrhagic Telangiectasia (HHT), where patients exhibit rapid vasculature expansion akin to the observed effects upon introducing the c-Src-CA mutation. In HHT, PTEN inhibition triggers heightened activity of VEGFA/VEGFR2 and subsequent AKT kinase activation. Although we have conducted preliminary analysis revealing elevated phospho-AKT (*Revision Figure 3*), we contend

© 2024. Published by The Company of Biologists under the terms of the Creative Commons Attribution License (https://creativecommons.org/licenses/by/4.0/).

that an in-depth examination of each signalling pathway perturbed downstream of c-Src-CA is beyond the scope of this manuscript. Our future studies will specifically address this, providing a meticulous exploration of c-Src activity in HHT and its intricate interaction with the AKT pathway.

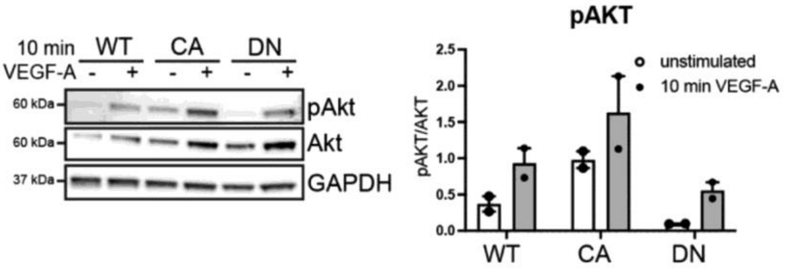

***Revision Figure 3**. **c-Src activity can alter AKT signalling**. HUVEC were transduced with c-Src mutant virus and stimulated with VEGF-A for 10 min (100 ng/ml). Preliminary results suggests that c-Src-CA cells display increased pAKT. n=2 independent experiments.*

Point 6. "Taken together, these results reveal that proteases produced by c-Src-CA cells are locally secreted at FAs but are membrane bound."
The claim that proteases are membrane-bound is not convincingly demonstrated.
Could the authors assess whether the constitutive form of cSrc activates the expression of specific genes encoding MMPs by qPCR? Or is it more a matter of the effect of c-Src on the transport of MMPs by microtubules?

We would like to clarify the content of Figure 5, which presents two distinct sets of experiments supporting the assertion that the proteases under investigation are membrane-bound. Firstly, the transfer of conditioned medium from c-Src mutant cells demonstrated no degradation of fibronectin fibrils. Secondly, in the bead sprouting assay, a mixed culture of untransduced and c-Src-CA expressing cells was utilised. The results revealed that only c-Src-CA cells formed balloons, while untransduced cells sprouted normally right next, to or sometimes even through, a balloon.

Recognising the need for a more in-depth understanding, we acknowledge the importance of analysing specific MMP gene expression. To this end, we have performed qPCR analysis for MMP7, MMP9 and MT1-MMP (MMP14). We did not detect any significant difference for either MMP7, MMP9 or MT1- MMP in c-Src-CA cells compared to control or the other c-Src mutants. This suggest that c-Src-CA most likely does not alter MMP gene expression, but rather enhances transport and secretion. However, there is a wide variation between experimental replicates, which warrants caution about drawing conclusions on the regulation of MMP gene expression by c-Src. Therefore, while results have been provided here (***Revision Figure 4A***), we have not included them in the manuscript.

In order to shed light on whether expression of specific MMPs are induced upon c-Src activation, we also performed a human MMP antibody array, which tests the concentration of secreted (supernatant) and intracellular (cell lysate) MMP2 and MMP9. No significant differences upon transduction with c-Src mutants was observed (***Revision Figure 4B***). Since we asserted that the proteases responsible for the ballooning phenotype in the c-Src-CA cells are membrane-bound, we attempted immunofluorescent staining for membrane bound MT1-MMP (MMP14). However, we were not able to reliably validate the antibody for IF staining (***Revision Figure 4C***). In summary, for this manuscript we have not been able to conclude which specific MMP may be responsible for the ballooning phenotype. This will be the focus of future studies.

© 2024. Published by The Company of Biologists under the terms of the Creative Commons Attribution License (https://creativecommons.org/licenses/by/4.0/).

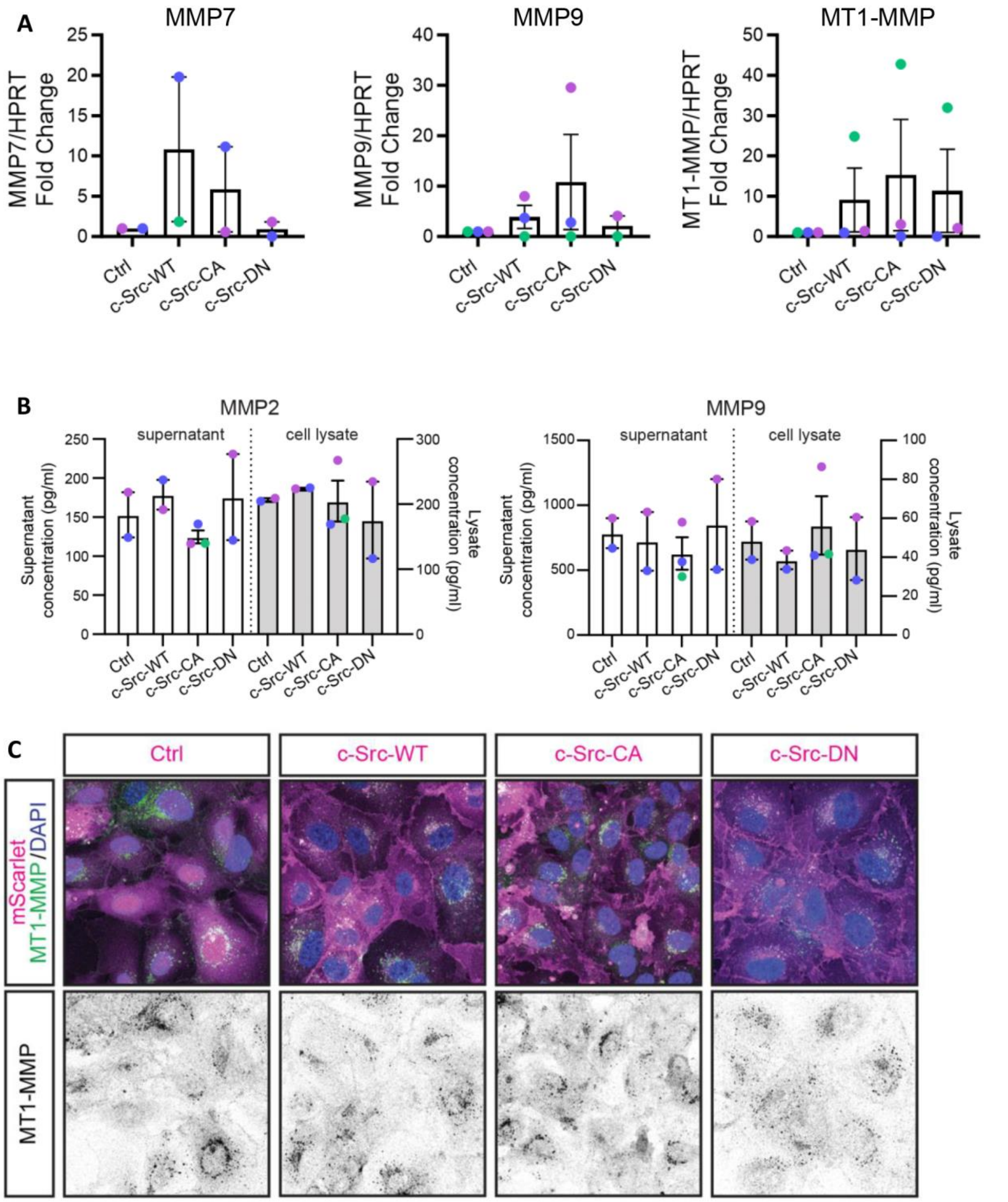

***Revision Figure 4. Investigation of MMP expression in c-Src mutant cells**. **(A)** HUVEC were transduced with mScarlet-tagged c-Src mutants and harvested for qPCR. There was no significant change in transcription of MMP7, MMP9 or MT1-MMP(MMP14). n = 2-3 independent experiments. **(B)** HUVEC were transduced with mScarlet-tagged c-Src mutants and both supernatant and cell lysates were harvested and analysed using a human MMP antibody array (Abcam). There was no significant change in secretion (supernatant) or production (cell lysate) of MMP2 or MMP9. n = 2 (Ctrl, c-Src-WT, c-Src-DN) or 3 (c-Src-CA) independently collected samples run on 1 array. **(C)** HUVEC transduced with mScarlet-tagged c-Src mutants (magenta). Immunofluorescent staining was performed for MT1-MMP (green and grey individual channel) and nuclei (DAPI; blue).*

Minor comments:

Point 1. General comment: The authors have predominantly presented composite images with

© 2024. Published by The Company of Biologists under the terms of the Creative Commons Attribution License (https://creativecommons.org/licenses/by/4.0/).

overlapping staining, making it challenging to differentiate between different labels. It would be beneficial if the authors could provide individual channel images along with a merge.

Given the large numbers of multi-channel composite images, it is not feasible to show each individual channel of every merged image in the manuscript. We have included individual channel images where we believe is appropriate. For example, for p-paxillin Y118 (Figure 2) and fibronectin (Figure 4).
Additionally, we have now added individual channel images in Figure 1F'' for the VE-cadherin channel, Figure 5A for the fibronectin channel, and Supp Figure 2A, C and I for the Ki-67, EdU and Cleaved Caspase 3 channels.

Point 2. The lab already showed in a previous study that mice lacking c-Src specifically in endothelial cells have reduced blood vessel sprouting, leading to the expectation that the constitutively active form of cSrc would increase sprout number in the sprouting assay. Could the authors explain why the constitutively active form of cSrc induces this vascular ballooning and not an increase in the number of sprouts?

As suggested by Reviewer 1 (point 3) and discussed above, Notch signalling plays a fundamental role in selecting tip cells and might be impacted by expression of constitutive active form of c-Src. Therefore, we have undertaken additional experiments cells to assess Notch signalling upon c-Src activation. The data has been added to Supp Fig 1F-G and above, and the potential changes in Notch signalling upon c- Src-CA expression have been discussed on page 5.

Point 3. In Figure 1A, it would be beneficial to include images from orthogonal views. Indeed, in the c- Src-CA condition, it's not clear whether the vascular ballooning observed represents a cluster of cells or an empty space between the bead and the endothelial cells. (Supp movie 1 helps, but it would be useful to add orthogonal views to the figure)
For clarity, we have added single Z-plane images for cross sectional views of the bead sprouts in Figure 1A, inset panel. These images reveal that the c-Src-CA cells have an empty space inside the balloon, rather than being a big cluster of cells.

Point 4. In Figure 1D, the method used to analyze sprout shape is not clear, especially for the c-Src-CA condition where the number of sprouts is close to 0. The figure legend indicates that this measurement corresponds to the shape of the sprouting area. Could the authors clarify and explain their quantification method?

The shape of the sprouting area refers to the circularity index of the vascular area, measured by tracing the perimeter of the cell area in a minimum Z-projection of brightfield images and subtracting the area of the bead. For improved clarity, we have adjusted the title of Figure 1D and Figure 6D to 'Vascular area shape' and added details of the quantification method in the methods section on page 18.
Point 5. "however cells within the vessel still maintained come connections (Fig 1E')": The connections between cells are difficult to see in the images in Figure 1E'. Could the authors provide higher magnification images of the VE-cadherin staining to illustrate these connections between cells?

For improved clarity, we have added high magnification images of the VE-cadherin channel only in black and white (Figure 1F'') and indicated some of the maintained cell-cell connections in the c-Src-CA cells with black arrowheads.

Point 6. "The reduction in migration correlated with an increase in FA size c-Src-CA expressing cells.": Could the authors give more explanation?

We have adjusted phrasing to provide additional information (page 7) as follows:
'The reduction in migration velocity in c-Src-CA cells coincides with an increase in FA size, number and density (Fig 2A-D). This suggests that the reduction of migration velocity is due to increased cellular adhesion via FAs.'
Point 7. Could the authors widen the cell trajectory trace in Supplementary Figure 3A?

We have adjusted the thickness of the trajectory lines in the figure (now Supplementary Figure 4A)

© 2024. Published by The Company of Biologists under the terms of the Creative Commons Attribution License (https://creativecommons.org/licenses/by/4.0/).

for improved visibility.
Point 8. It is very difficult to distinguish fibronectin fibrils on the images shown in figure 4C. it would be beneficial to change the images.

We have enlarged the zoomed areas for better visibility of the focal adhesions and fibronectin degradation underneath those areas in the c-Src-CA cells. Additionally, arrows have been added to indicate fibronectin fibrils in Figure 4C.

Point 9. "Treatment of ECs with Marimastat in a fibrin bead sprouting assay resulted in a rescue of the ballooning morphology observed in the c-Src-CA cells"
Based on the images displayed in the figure and the associated quantifications, it still appears that c-Src- CA+Marimastat induces a vascular ballooning even if it is less pronounced than in the DMSO condition. Hence, it would be more accurate to describe the observed effect as a "partial rescue". In the microfabricated 3D vessel, in the figure 7A, cell-cell junctions still appear altered by c-Src-CA after the treatment with Marimastat, compared to the c-Src-WT-Marimastat, it would be more appropriate to talk about "partial rescue".
We have changed 'rescue' to 'partial rescue' when referring to results in Figure 6 and 7 (page 9).

Point 10. In Figure 6A, it seems that there is a decrease in the number of sprouts in the c-Src-DN condition compared to the control condition after the DMSO treatment, which is not observed in Figure 1, could the authors explain why?

In Figure 1C, the number of sprouts is also reduced in the c-Src-DN condition compared to c-Src-WT, but this is not significant when compared to control (see Supplementary Table 1 for p values of all comparisons). However, it is true that the number of sprouts in the c-Src-DN condition is significantly reduced compared to both control and c-Src-WT upon DMSO treatment (Fig 6C). Reduction of sprouts in c-Src-DN cells was expected due to the dysfunctional kinase domain, as mentioned on page 5 and shown in reference 30 (Shvartsman, D.E., et al., J Cell Biol, 2007. 178(4): p. 675-86.). Why DMSO treatment seems to enhance the effects of dominant negative c-Src expression on sprouting behaviour remains unclear. However, DMSO has adverse effects on sprouting generally, shown by reduction of sprouts in both control and c-Src-WT cells (comparing untreated condition in Fig 1C with DMSO treated condition in Fig 6C). We therefore conclude that DMSO treatment has adverse effects on sprouting in all settings.

Point 11. There is no statistical paragraph in the method section.

As pointed out by reviewer 1 and 2, we have now added a general section on the statistical methods to the method section on page 18. Additional details on the tests used for each specific graph can be found in the figure legends and Supplementary Table 1.

Second decision letter

MS ID#: JOCES/2024/262101

MS TITLE: c-Src induced vascular malformations require localised matrix degradation at focal adhesions

AUTHORS: Patricia Essebier, Mikaela Keyser, Teodor Yordanov, Brittany Hill, Alexander Yu, Ivar Noordstra, Alpha S. Yap, Samantha J Stehbens, Anne Lagendijk, Lilian Schimmel, and Emma Gordon

Thank you for sending your manuscript to Journal of Cell Science through Review Commons.

I am happy to tell you that your manuscript has been accepted for publication in Journal of Cell Science, pending standard ethics checks.

© 2024. Published by The Company of Biologists under the terms of the Creative Commons Attribution License (https://creativecommons.org/licenses/by/4.0/).
